# Supplementary material for: The sources and transmission routes of microbial populations throughout a meat processing facility
Source: NPJ Biofilms Microbiomes. 2020 Jul 10;6:26. doi: 10.1038/s41522-020-0136-z (PMC7351959; doi:10.1038/s41522-020-0136-z)
Supplement: Supplementary file 1 — Supplementary Information [file 41522_2020_136_MOESM1_ESM.pdf]

Supplementary Information: Zwirzitz et al.

The sources and transmission routes of microbial populations throughout a meat processing facility

Supplementary Table 1-5

Supplementary Figures 1-7

Supplementary Note 1

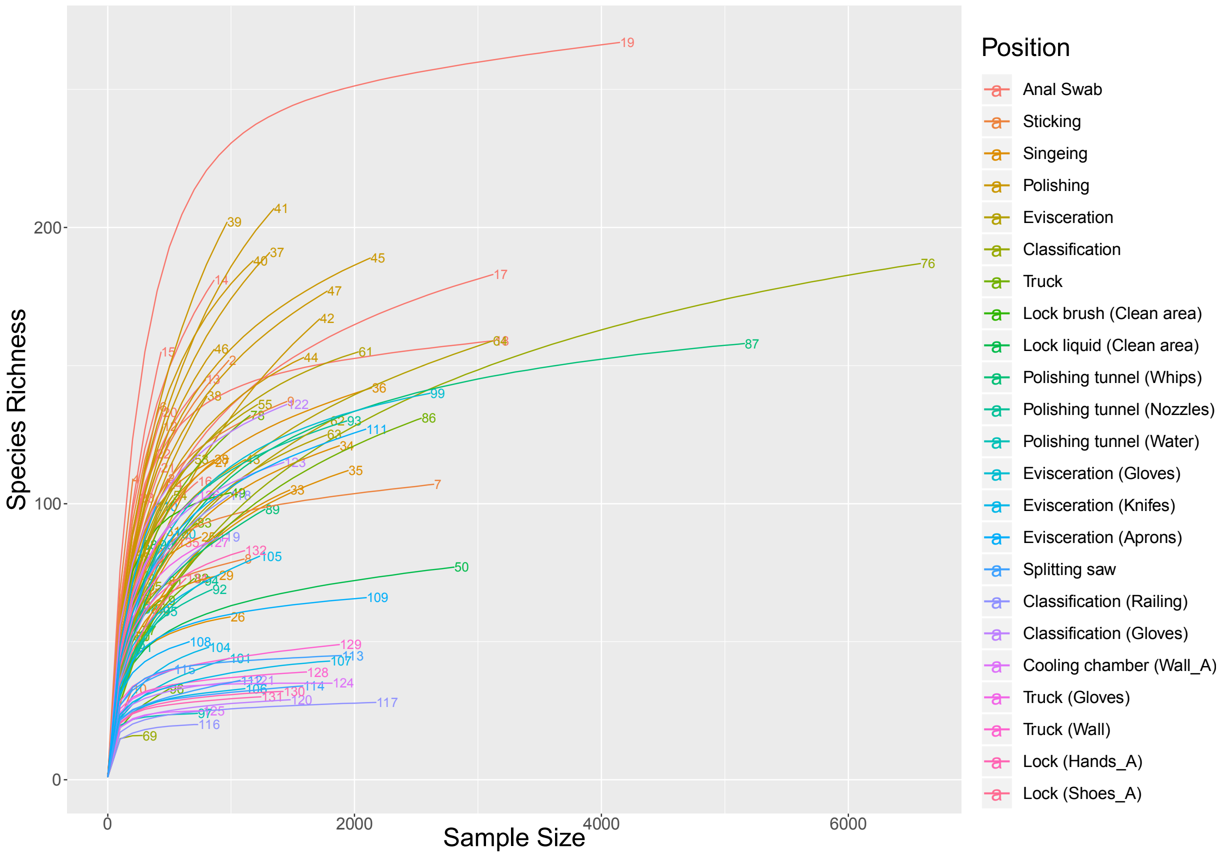

Supplementary Figure 1: *Rarefaction curve of individual 16S rRNA gene libraries*

Supplementary Table 1: *Prevalence of genera comprising top 50 ASVs*

Genus	Prevalence	No. of ASVs
Anoxybacillus	72	59
Bacillus_L	33	9
Bacillus_S	72	29
Caldimonas	48	2
Chryseobacterium	85	57
Chryseobacterium_D	67	15
Curvibacter_A	15	1
Cutibacterium	37	8
Enhydrobacter	92	47
Family_Bacillaceae_D	41	2
Family_Burkholderiaceae	75	19
Family_Weeksellaceae	65	16
Family_Xanthomonadaceae	67	18
Flavobacterium_A	77	28
Helicobacter_F	5	11
Lactobacillus	41	5
Lactococcus	21	7
Luteimonas	52	8
Luteimonas_A	45	9
Lysinibacillus	40	6
Microbacterium	77	10
Moraxella	69	17
Ochrobactrum_A	58	3
Paracoccus	87	46
Vitreoscilla	52	16
W16RD	3	5

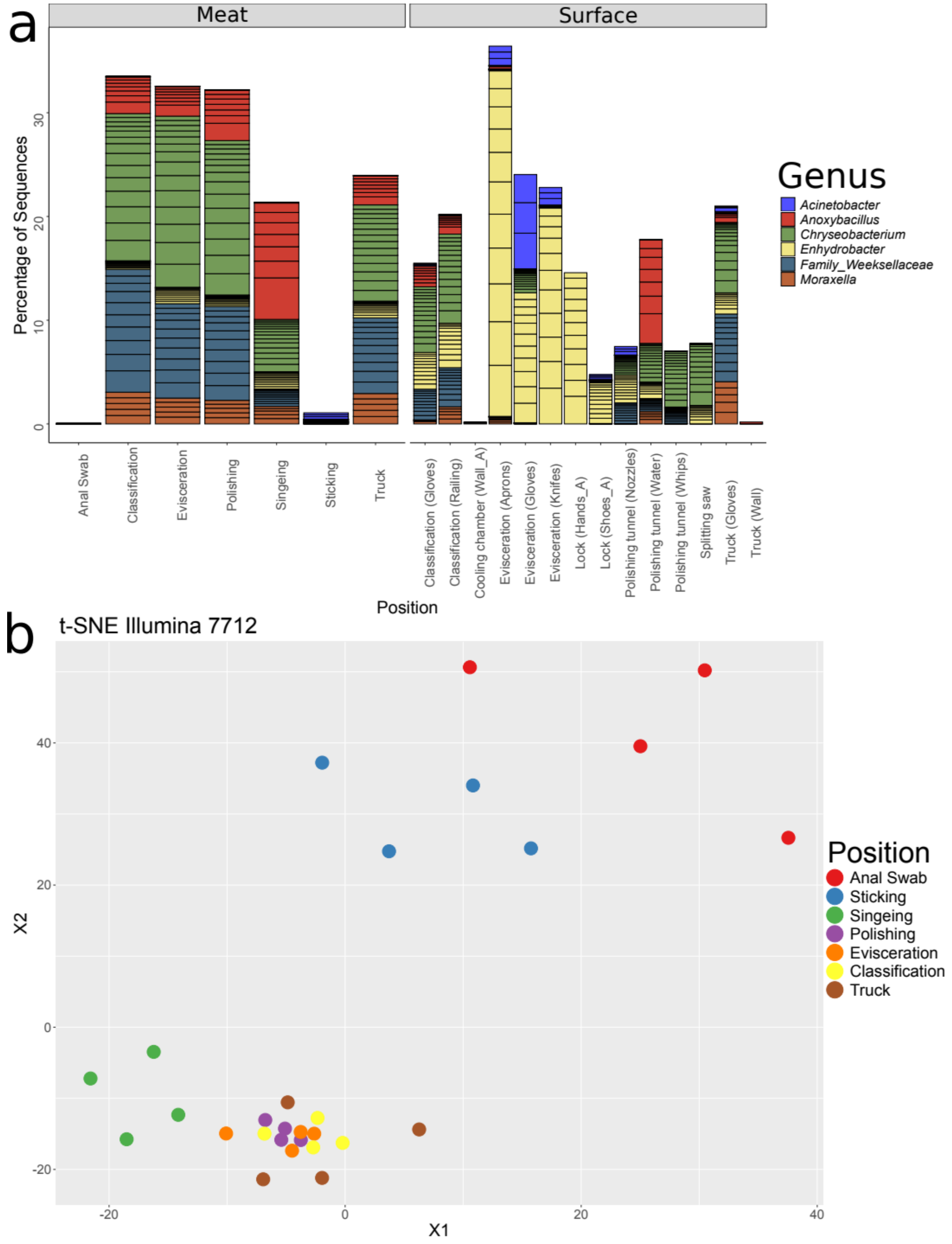

Supplementary Figure 2: *A) Genus-level classification of the 50 most abundant ASVs of the Illumina dataset parted by type (Meat or Surface). Data represents average of ASV counts from replicate libraries for each category. Individual ASVs are separated by a black line within the bar graph. ASVs assigned to candidate genera that do not have a name assignment yet are indicated with "Family_". B) t-SNE plot of Bray-Curtis distances based on 16S rRNA gene libraries of the Illumina dataset rarefied to 7,712 sequences per sample. Each point represents values from individual libraries with colors expressing meat samples from different positions along the processing line.*

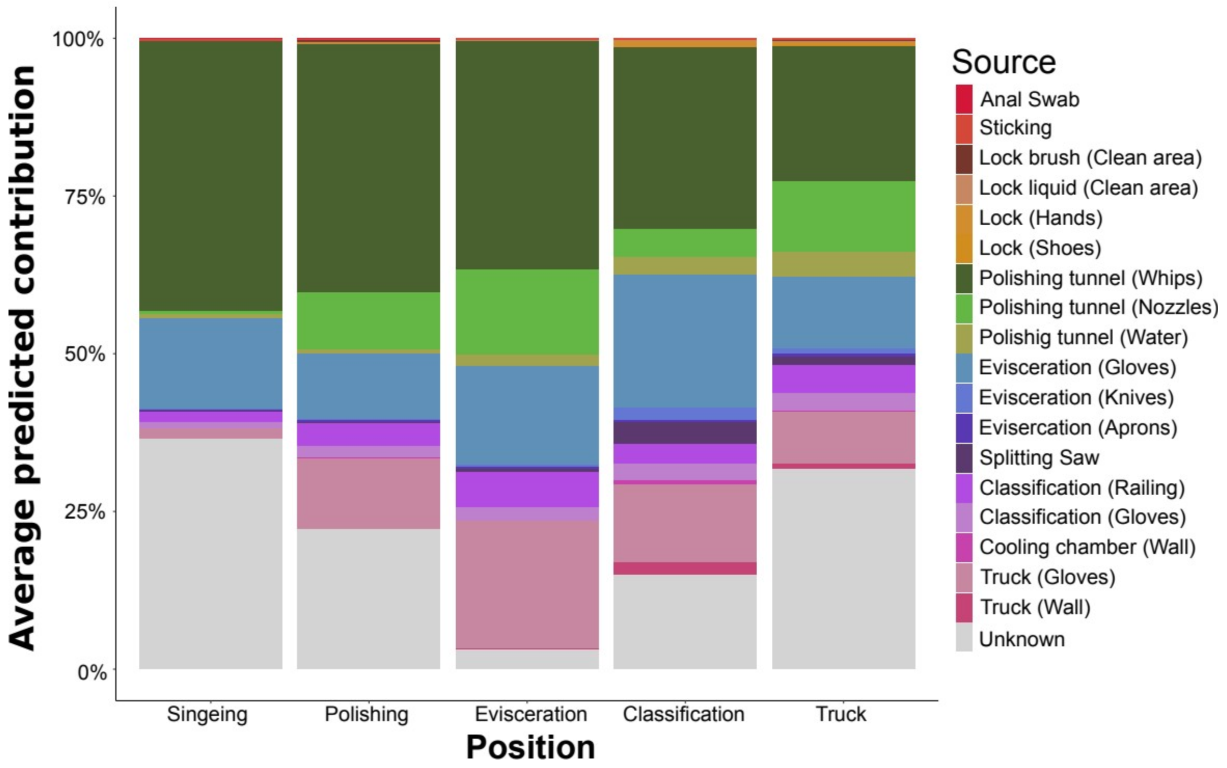

Supplementary Figure 3: *Results of SourceTracker analysis showing the predicted average contributions of different sources to individual meat-associated bacterial communities at all sampling positions.*

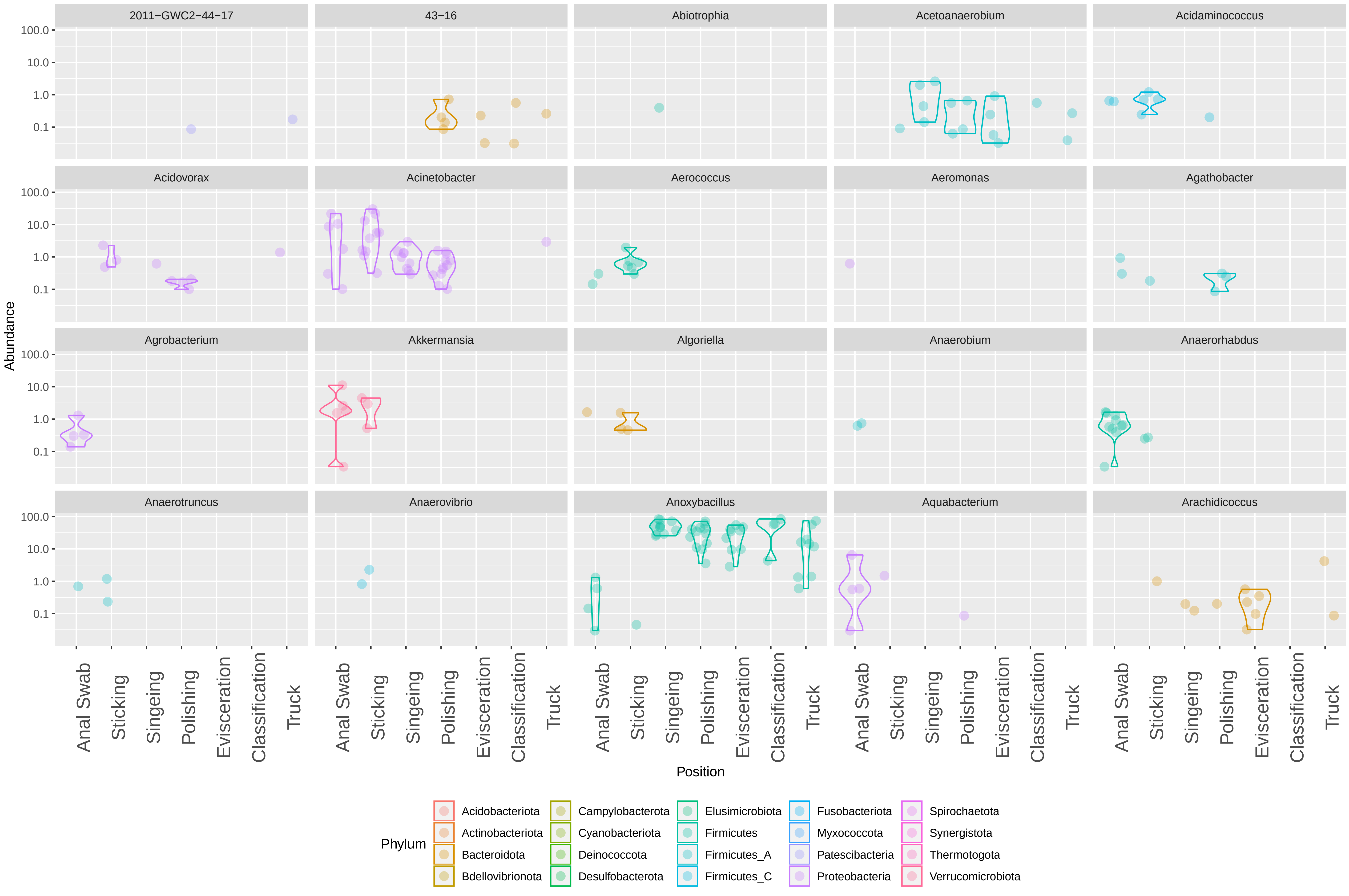
2011−GWC2−44−17
43−16
Abiotrophia
Acetoanaerobium
Acidaminococcus
Acidovorax
Acinetobacter
Aerococcus
Aeromonas
Agathobacter
Agrobacterium
Akkermansia
Algoriella
Anaerobium
Anaerorhabdus
Anaerotruncus
Anaerovibrio
Anoxybacillus
Aquabacterium
Arachidicoccus
100.0
10.0
1.0
0.1
Abundance
Anal Swab
Sticking
Singeing
Polishing
Evisceration
Classification
Truck
Position
Phylum
Acidobacteriota
Actinobacteriota
Bacteroidota
Bdellovibrionota
Campylobacterota
Cyanobacteriota
Deinococcota
Desulfobacterota
Elusimicrobiota
Firmicutes
Firmicutes_A
Firmicutes_C
Fusobacteriota
Myxococcota
Patescibacteria
Proteobacteria
Spirochaetota
Synergistota
Thermotogota
Verrucomicrobiota

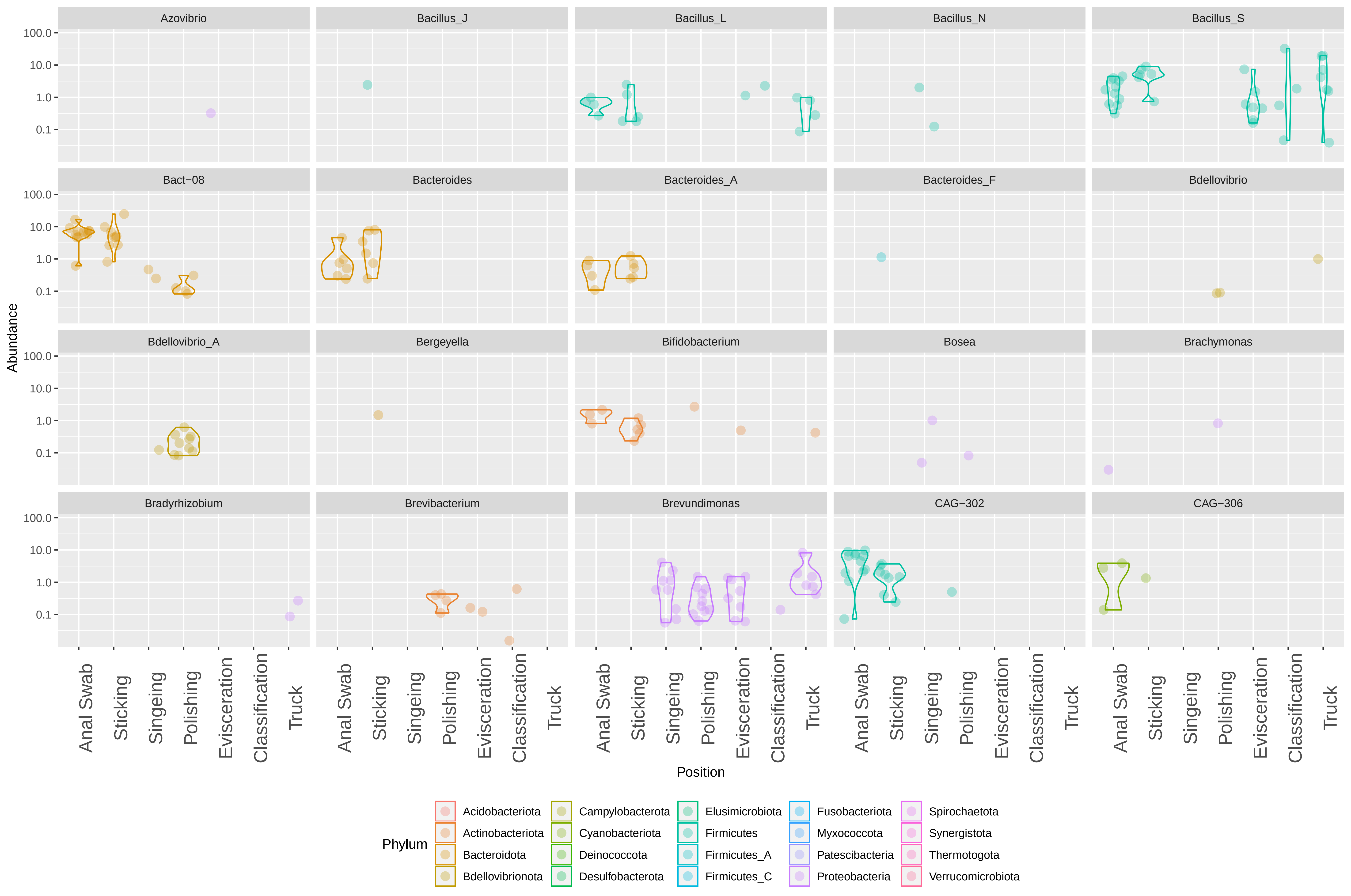
Azovibrio
Bacillus_J
Bacillus_L
Bacillus_N
Bacillus_S
Bact−08
Bacteroides
Bacteroides_A
Bacteroides_F
Bdellovibrio
Bdellovibrio_A
Bergeyella
Bifidobacterium
Bosea
Brachymonas
Bradyrhizobium
Brevibacterium
Brevundimonas
CAG−302
CAG−306
Abundance
100.0
10.0
1.0
0.1
Anal Swab
Sticking
Singeing
Polishing
Evisceration
Classification
Truck
Position
Phylum
Acidobacteriota
Actinobacteriota
Bacteroidota
Bdellovibrionota
Campylobacterota
Cyanobacteriota
Deinococcota
Desulfobacterota
Elusimicrobiota
Firmicutes
Firmicutes_A
Firmicutes_C
Fusobacteriota
Myxococcota
Patescibacteria
Proteobacteria
Spirochaetota
Synergistota
Thermotogota
Verrucomicrobiota

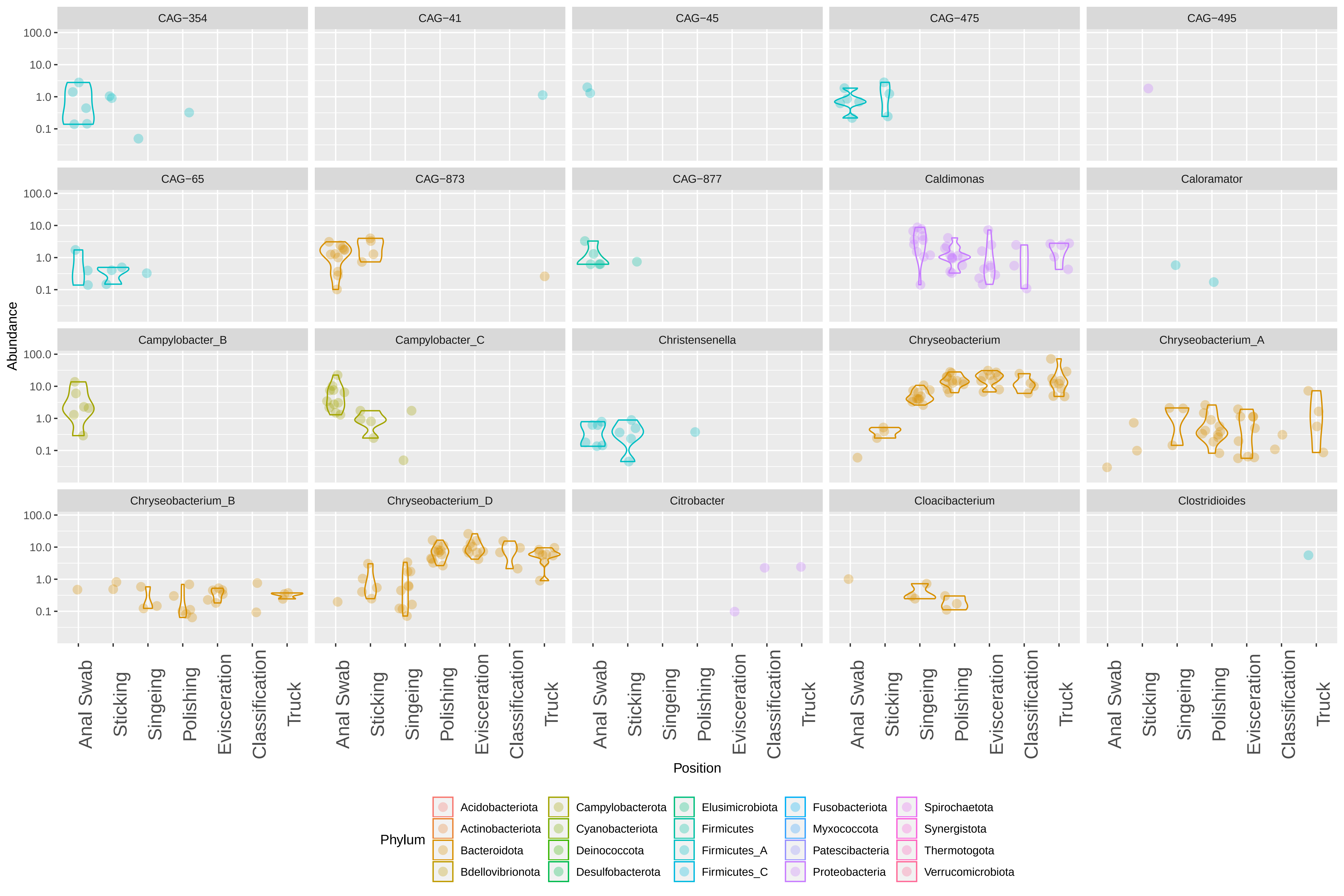
CAG−354
CAG−41
CAG−45
CAG−475
CAG−495
CAG−65
CAG−873
CAG−877
Caldimonas
Caloramator
Campylobacter_B
Campylobacter_C
Christensenella
Chryseobacterium
Chryseobacterium_A
Chryseobacterium_B
Chryseobacterium_D
Citrobacter
Cloacibacterium
Clostridioides
Abundance
100.0
10.0
1.0
0.1
Anal Swab
Sticking
Singeing
Polishing
Evisceration
Classification
Truck
Position
Phylum
Acidobacteriota
Actinobacteriota
Bacteroidota
Bdellovibrionota
Campylobacterota
Cyanobacteriota
Deinococcota
Desulfobacterota
Elusimicrobiota
Firmicutes
Firmicutes_A
Firmicutes_C
Fusobacteriota
Myxococcota
Patescibacteria
Proteobacteria
Spirochaetota
Synergistota
Thermotogota
Verrucomicrobiota

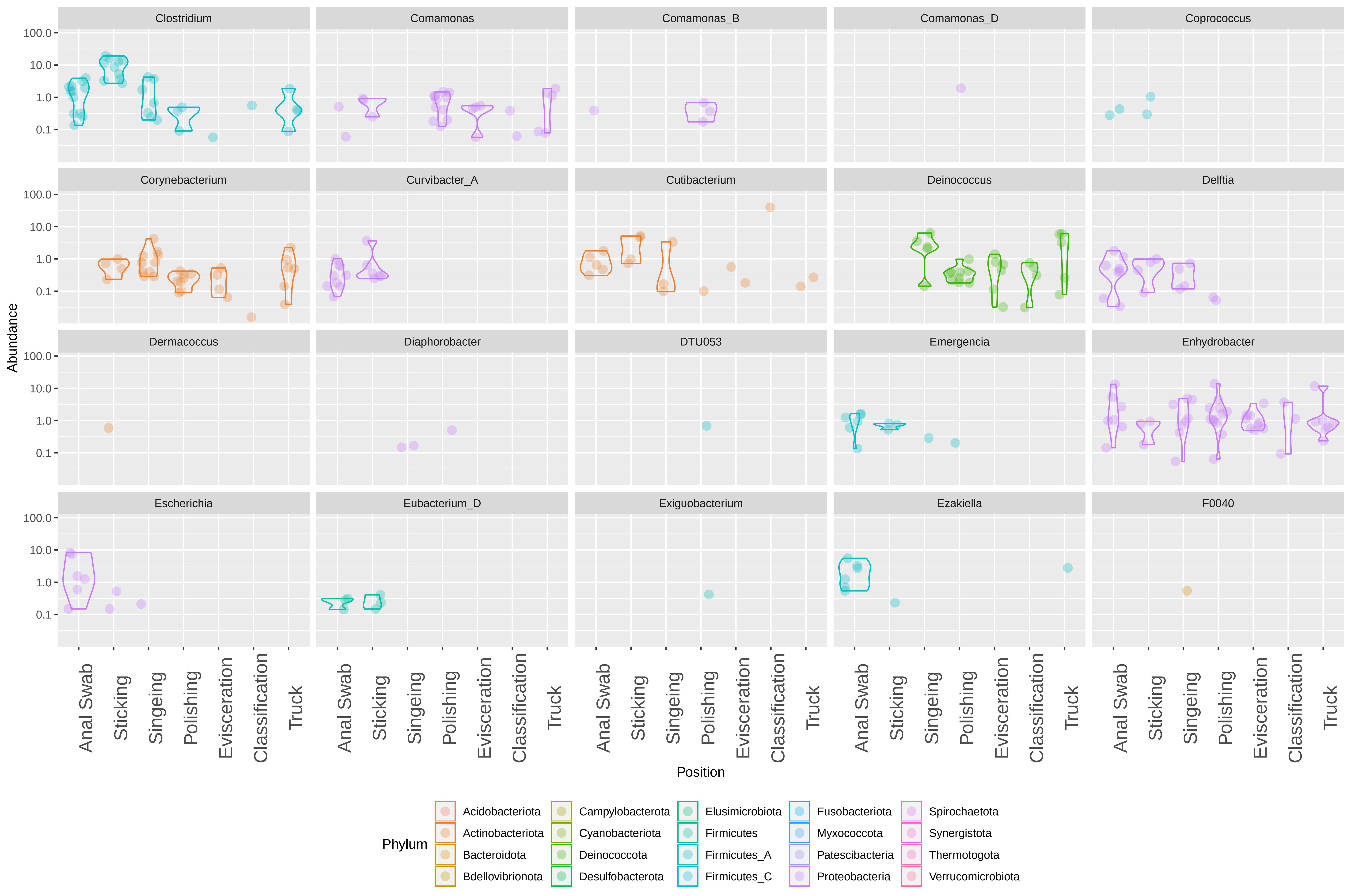
Clostridium
Comamonas
Comamonas_B
Comamonas_D
Coprococcus
Corynebacterium
Curvibacter_A
Cutibacterium
Deinococcus
Delftia
Dermacoccus
Diaphorobacter
DTU053
Emergencia
Enhydrobacter
Escherichia
Eubacterium_D
Exiguobacterium
Ezakiella
F0040
100.0
10.0
1.0
0.1
Abundance
Anal Swab
Sticking
Singeing
Polishing
Evisceration
Classification
Truck
Position
Phylum
Acidobacteriota
Actinobacteriota
Bacteroidota
Bdellovibrionota
Campylobacterota
Cyanobacteriota
Deinococcota
Desulfobacterota
Elusimicrobiota
Firmicutes
Firmicutes_A
Firmicutes_C
Fusobacteriota
Myxococcota
Patescibacteria
Proteobacteria
Spirochaetota
Synergistota
Thermotogota
Verrucomicrobiota

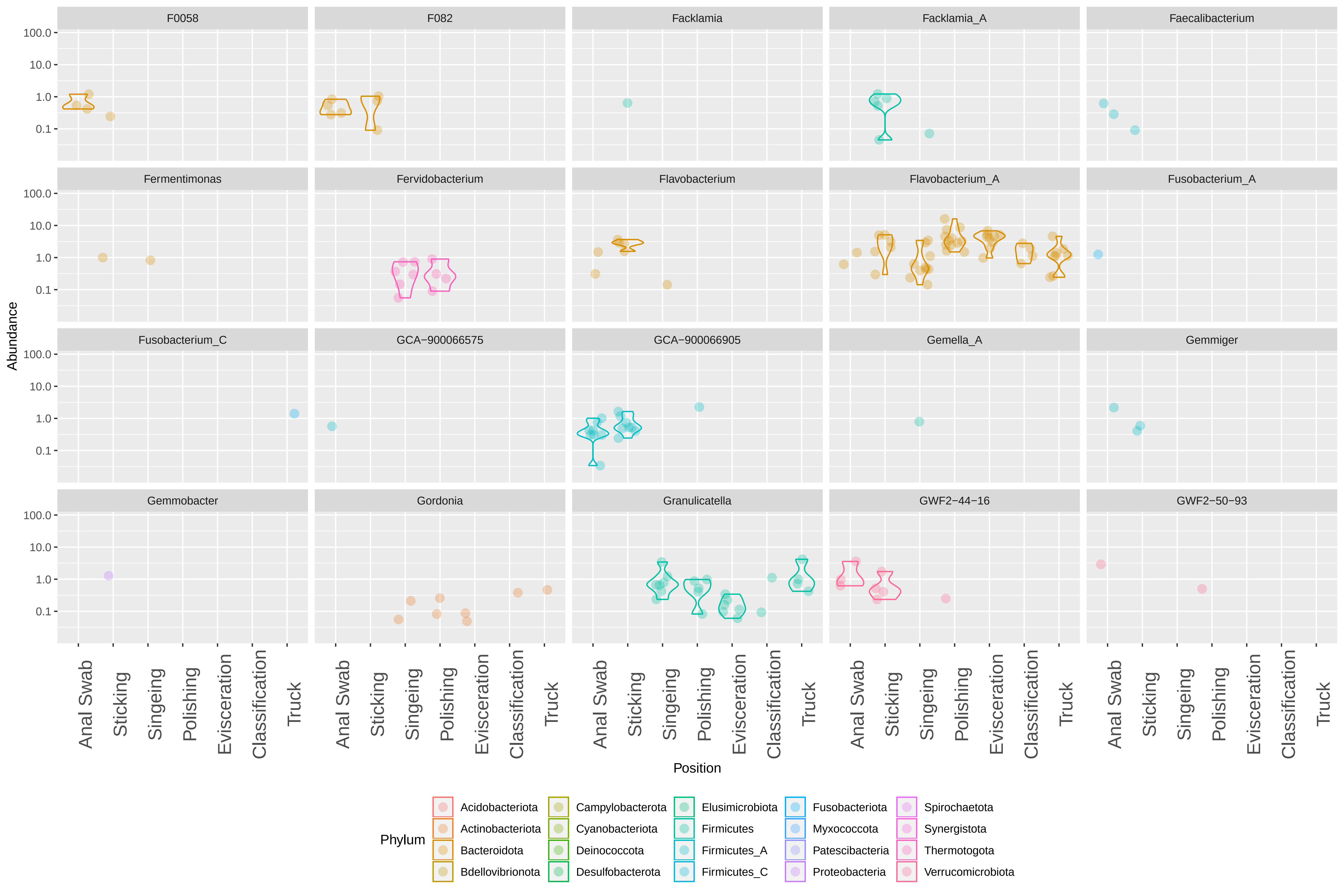
F0058
F082
Facklamia
Facklamia_A
Faecalibacterium
Fermentimonas
Fervidobacterium
Flavobacterium
Flavobacterium_A
Fusobacterium_A
Fusobacterium_C
GCA−900066575
GCA−900066905
Gemella_A
Gemmiger
Gemmobacter
Gordonia
Granulicatella
GWF2−44−16
GWF2−50−93
Abundance
100.0
10.0
1.0
0.1
Anal Swab
Sticking
Singeing
Polishing
Evisceration
Classification
Truck
Position
Phylum
Acidobacteriota
Actinobacteriota
Bacteroidota
Bdellovibrionota
Campylobacterota
Cyanobacteriota
Deinococcota
Desulfobacterota
Elusimicrobiota
Firmicutes
Firmicutes_A
Firmicutes_C
Fusobacteriota
Myxococcota
Patescibacteria
Proteobacteria
Spirochaetota
Synergistota
Thermotogota
Verrucomicrobiota

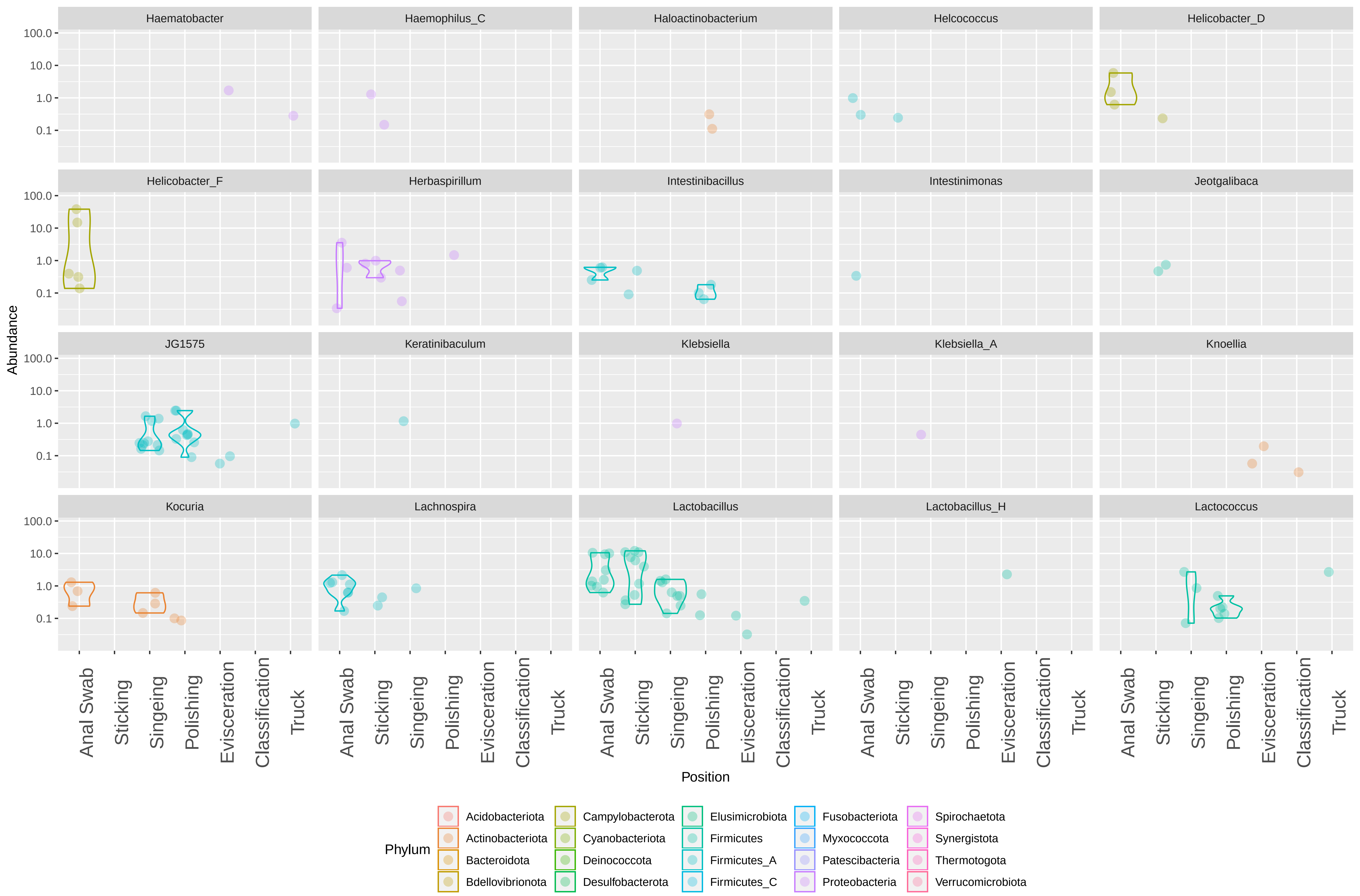
Haematobacter
Haemophilus_C
Haloactinobacterium
Helcococcus
Helicobacter_D
Helicobacter_F
Herbaspirillum
Intestinibacillus
Intestinimonas
Jeotgalibaca
JG1575
Keratinibaculum
Klebsiella
Klebsiella_A
Knoellia
Kocuria
Lachnospira
Lactobacillus
Lactobacillus_H
Lactococcus
Abundance
100.0
10.0
1.0
0.1
Anal Swab
Sticking
Singeing
Polishing
Evisceration
Classification
Truck
Position
Phylum
Acidobacteriota
Actinobacteriota
Bacteroidota
Bdellovibrionota
Campylobacterota
Cyanobacteriota
Deinococcota
Desulfobacterota
Elusimicrobiota
Firmicutes
Firmicutes_A
Firmicutes_C
Fusobacteriota
Myxococcota
Patescibacteria
Proteobacteria
Spirochaetota
Synergistota
Thermotogota
Verrucomicrobiota

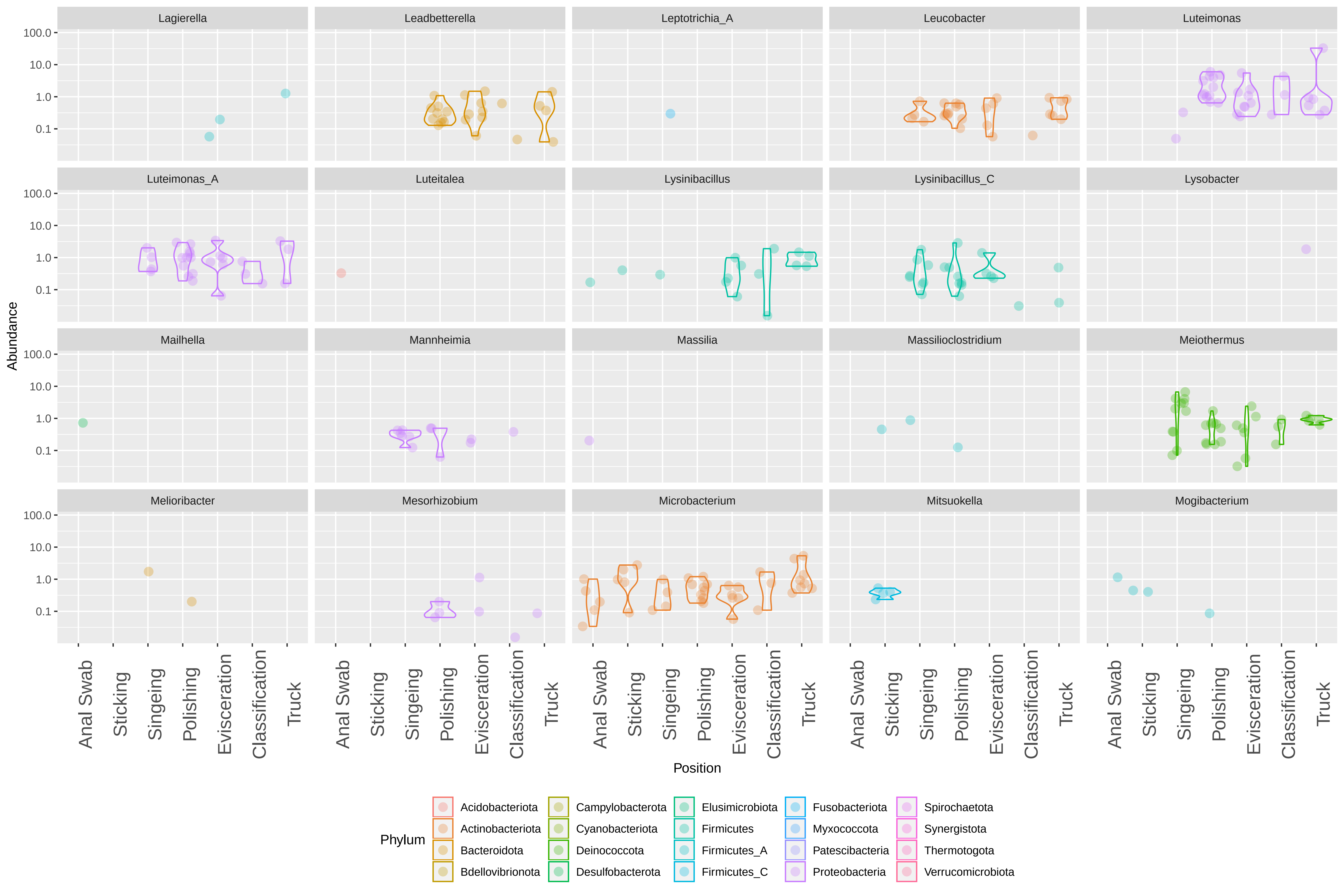

Lagierella
Leadbetterella
Leptotrichia_A
Leucobacter
Luteimonas
Luteimonas_A
Luteitalea
Lysinibacillus
Lysinibacillus_C
Lysobacter
Mailhella
Mannheimia
Massilia
Massilioclostridium
Meiothermus
Melioribacter
Mesorhizobium
Microbacterium
Mitsuokella
Mogibacterium
Abundance
100.0
10.0
1.0
0.1
Anal Swab
Sticking
Singeing
Polishing
Evisceration
Classification
Truck
Position
Phylum
Acidobacteriota
Actinobacteriota
Bacteroidota
Bdellovibrionota
Campylobacterota
Cyanobacteriota
Deinococcota
Desulfobacterota
Elusimicrobiota
Firmicutes
Firmicutes_A
Firmicutes_C
Fusobacteriota
Myxococcota
Patescibacteria
Proteobacteria
Spirochaetota
Synergistota
Thermotogota
Verrucomicrobiota

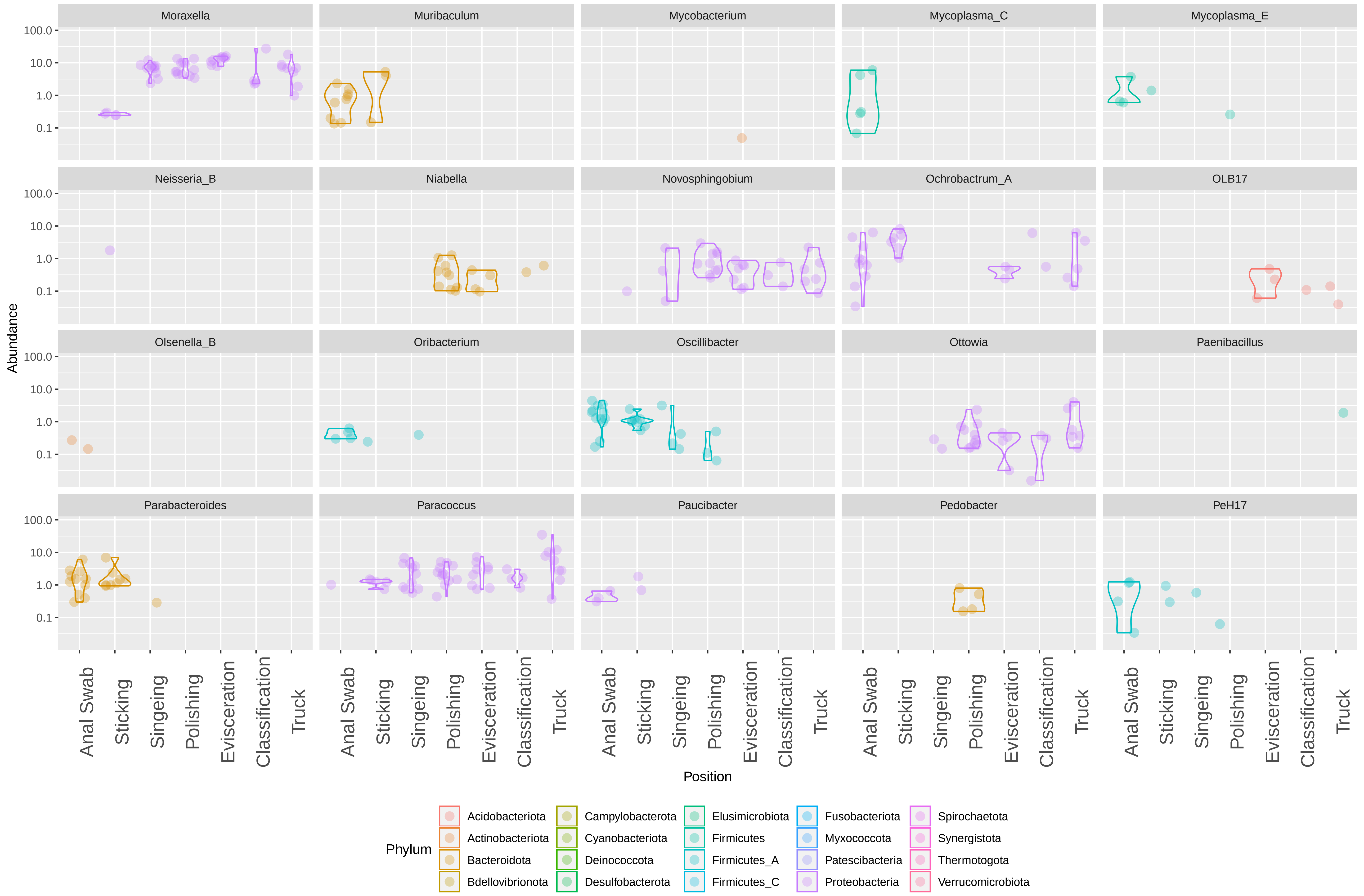

Moraxella
Muribaculum
Mycobacterium
Mycoplasma_C
Mycoplasma_E
Neisseria_B
Niabella
Novosphingobium
Ochrobactrum_A
OLB17
Olsenella_B
Oribacterium
Oscillibacter
Ottowia
Paenibacillus
Parabacteroides
Paracoccus
Paucibacter
Pedobacter
PeH17
Abundance
100.0
10.0
1.0
0.1
Anal Swab
Sticking
Singeing
Polishing
Evisceration
Classification
Truck
Position
Phylum
Acidobacteriota
Actinobacteriota
Bacteroidota
Bdellovibrionota
Campylobacterota
Cyanobacteriota
Deinococcota
Desulfobacterota
Elusimicrobiota
Firmicutes
Firmicutes_A
Firmicutes_C
Fusobacteriota
Myxococcota
Patescibacteria
Proteobacteria
Spirochaetota
Synergistota
Thermotogota
Verrucomicrobiota

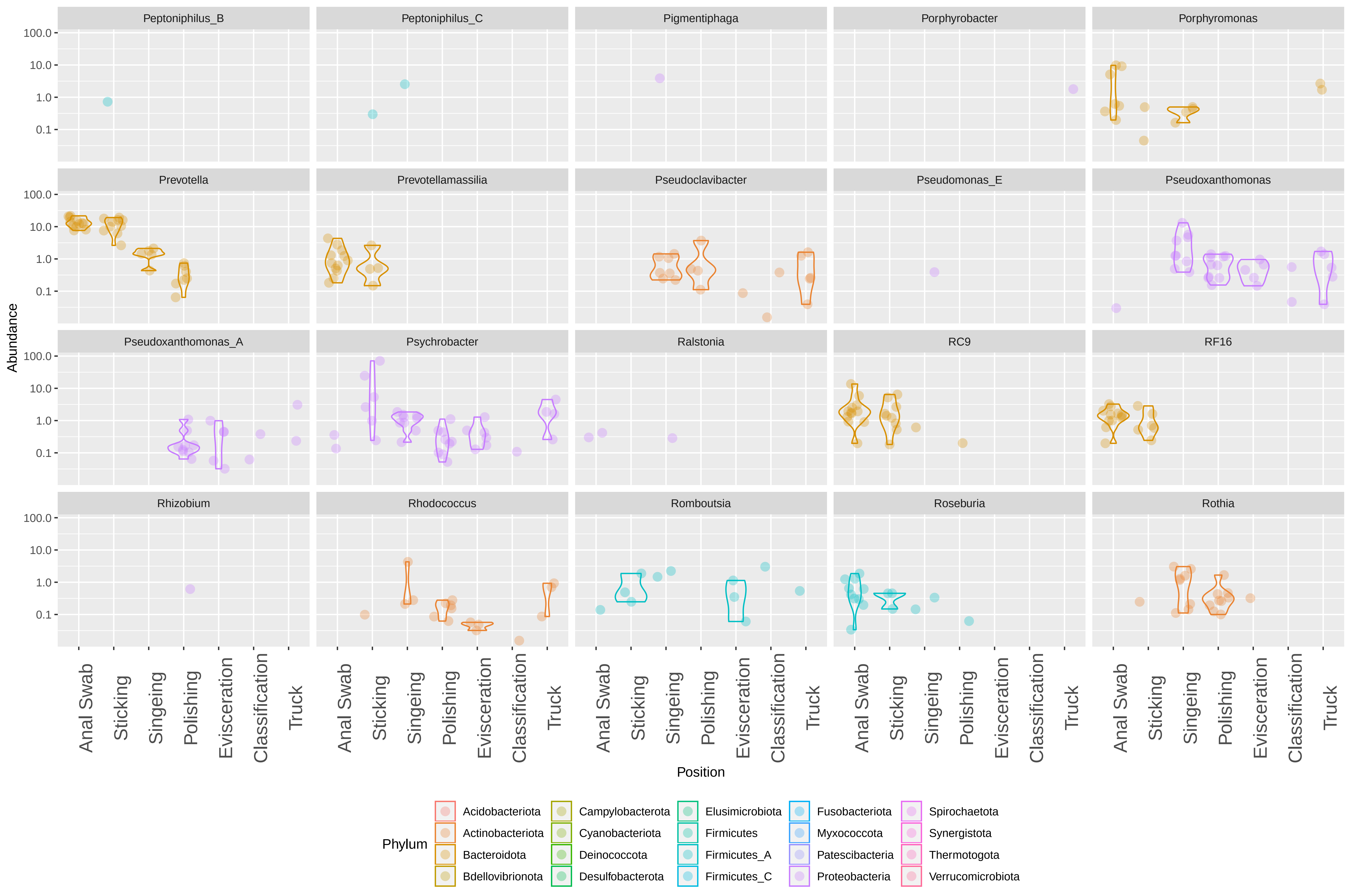

Peptoniphilus_B
Peptoniphilus_C
Pigmentiphaga
Porphyrobacter
Porphyromonas
Prevotella
Prevotellamassilia
Pseudoclavibacter
Pseudomonas_E
Pseudoxanthomonas
Pseudoxanthomonas_A
Psychrobacter
Ralstonia
RC9
RF16
Rhizobium
Rhodococcus
Romboutsia
Roseburia
Rothia
Abundance
100.0
10.0
1.0
0.1
Anal Swab
Sticking
Singeing
Polishing
Evisceration
Classification
Truck
Position
Phylum
Acidobacteriota
Actinobacteriota
Bacteroidota
Bdellovibrionota
Campylobacterota
Cyanobacteriota
Deinococcota
Desulfobacterota
Elusimicrobiota
Firmicutes
Firmicutes_A
Firmicutes_C
Fusobacteriota
Myxococcota
Patescibacteria
Proteobacteria
Spirochaetota
Synergistota
Thermotogota
Verrucomicrobiota

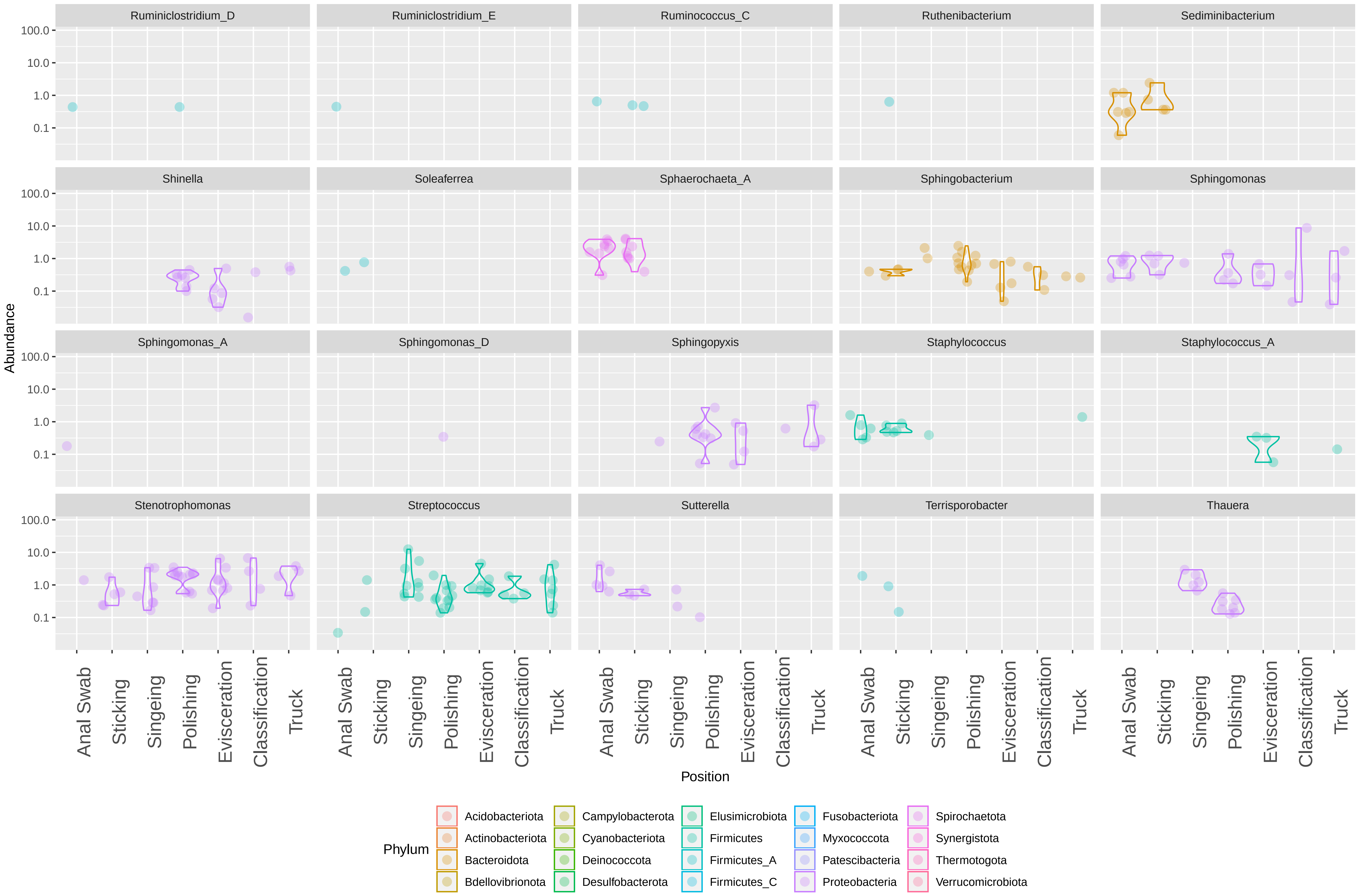
Ruminiclostridium_D
Ruminiclostridium_E
Ruminococcus_C
Ruthenibacterium
Sediminibacterium
Shinella
Soleaferrea
Sphaerochaeta_A
Sphingobacterium
Sphingomonas
Sphingomonas_A
Sphingomonas_D
Sphingopyxis
Staphylococcus
Staphylococcus_A
Stenotrophomonas
Streptococcus
Sutterella
Terrisporobacter
Thauera
Abundance
100.0
10.0
1.0
0.1
Anal Swab
Sticking
Singeing
Polishing
Evisceration
Classification
Truck
Position
Phylum
Acidobacteriota
Actinobacteriota
Bacteroidota
Bdellovibrionota
Campylobacterota
Cyanobacteriota
Deinococcota
Desulfobacterota
Elusimicrobiota
Firmicutes
Firmicutes_A
Firmicutes_C
Fusobacteriota
Myxococcota
Patescibacteria
Proteobacteria
Spirochaetota
Synergistota
Thermotogota
Verrucomicrobiota

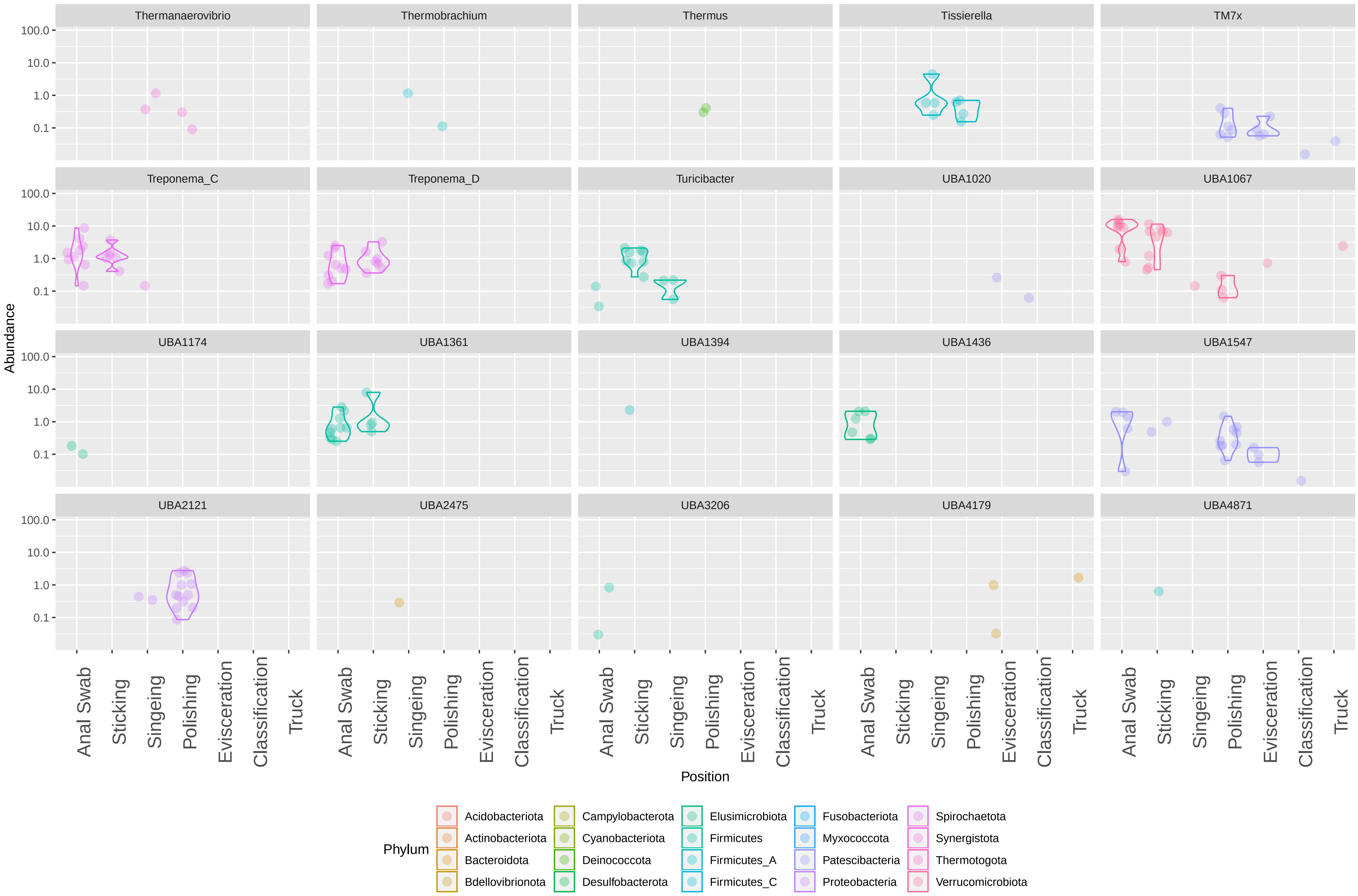
Thermanaerovibrio
Thermobrachium
Thermus
Tissierella
TM7x
Treponema_C
Treponema_D
Turicibacter
UBA1020
UBA1067
UBA1174
UBA1361
UBA1394
UBA1436
UBA1547
UBA2121
UBA2475
UBA3206
UBA4179
UBA4871
Abundance
100.0
10.0
1.0
0.1
Anal Swab
Sticking
Singeing
Polishing
Evisceration
Classification
Truck
Position
Phylum
Acidobacteriota
Actinobacteriota
Bacteroidota
Bdellovibrionota
Campylobacterota
Cyanobacteriota
Deinococcota
Desulfobacterota
Elusimicrobiota
Firmicutes
Firmicutes_A
Firmicutes_C
Fusobacteriota
Myxococcota
Patescibacteria
Proteobacteria
Spirochaetota
Synergistota
Thermotogota
Verrucomicrobiota

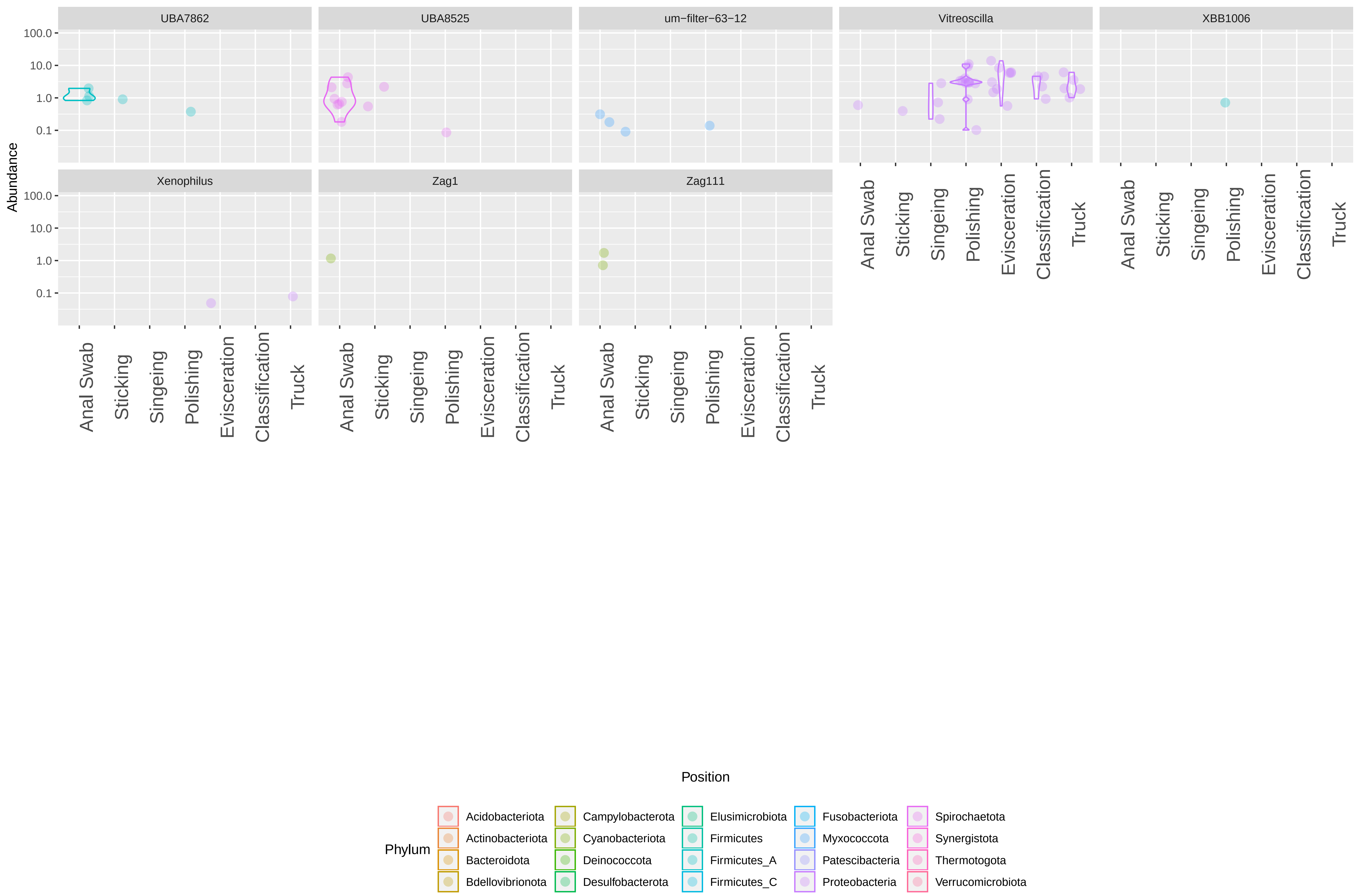
UBA7862
UBA8525
um−filter−63−12
Vitreoscilla
XBB1006
Xenophilus
Zag1
Zag111
Abundance
100.0
10.0
1.0
0.1
Anal Swab
Sticking
Singeing
Polishing
Evisceration
Classification
Truck
Position
Phylum
Acidobacteriota
Actinobacteriota
Bacteroidota
Bdellovibrionota
Campylobacterota
Cyanobacteriota
Deinococcota
Desulfobacterota
Elusimicrobiota
Firmicutes
Firmicutes_A
Firmicutes_C
Fusobacteriota
Myxococcota
Patescibacteria
Proteobacteria
Spirochaetota
Synergistota
Thermotogota
Verrucomicrobiota

Supplementary figure 4: *Relative abundances of all detected genera across all meat positions.*

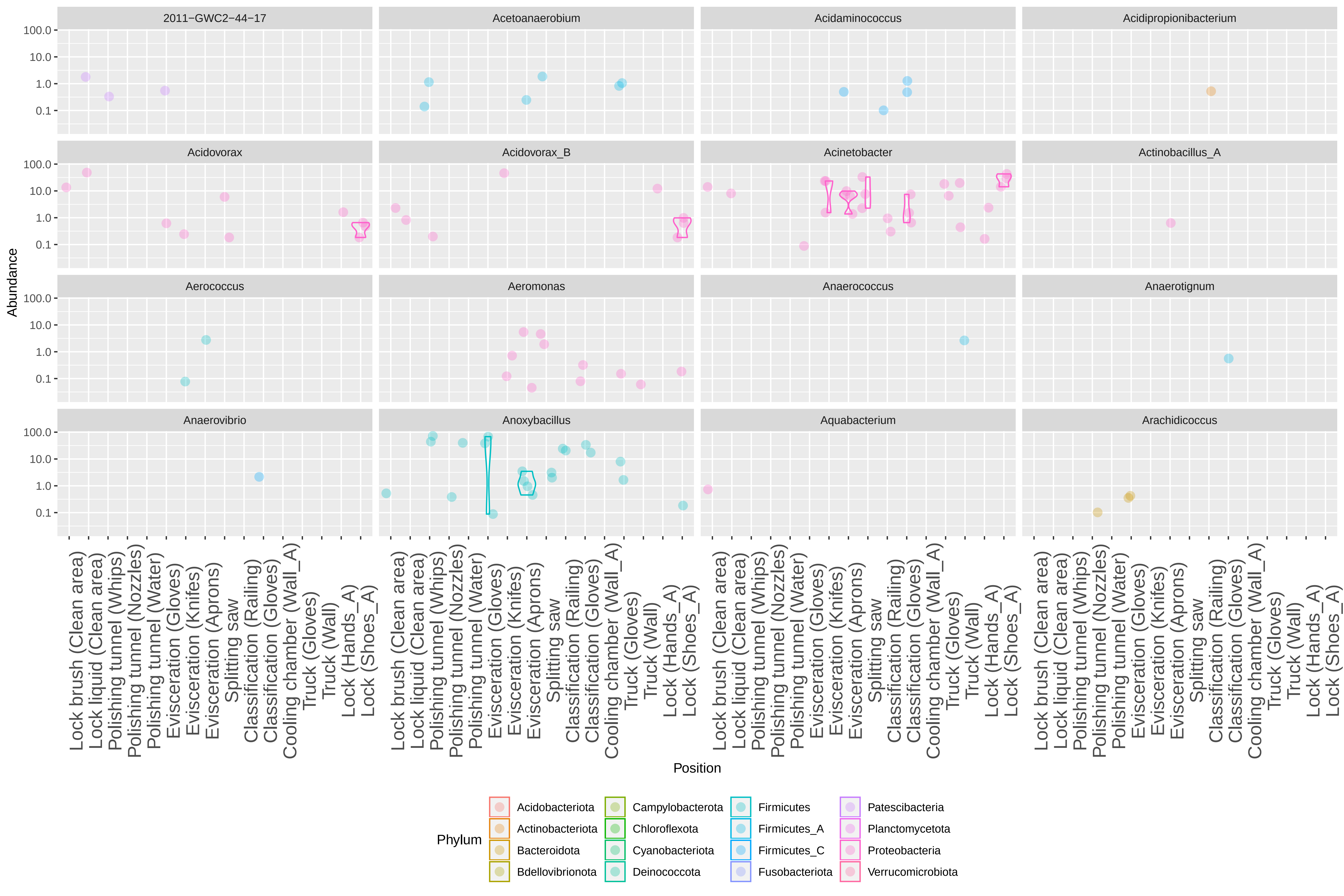
2011−GWC2−44−17
Acetoanaerobium
Acidaminococcus
Acidipropionibacterium
Acidovorax
Acidovorax_B
Acinetobacter
Actinobacillus_A
Aerococcus
Aeromonas
Anaerococcus
Anaerotignum
Anaerovibrio
Anoxybacillus
Aquabacterium
Arachidicoccus
Abundance
100.0
10.0
1.0
0.1
Position
Lock brush (Clean area)
Lock liquid (Clean area)
Polishing tunnel (Whips)
Polishing tunnel (Nozzles)
Polishing tunnel (Water)
Evisceration (Gloves)
Evisceration (Knifes)
Evisceration (Aprons)
Splitting saw
Classification (Railing)
Classification (Gloves)
Cooling chamber (Wall_A)
Truck (Gloves)
Truck (Wall)
Lock (Hands_A)
Lock (Shoes_A)
Phylum
Acidobacteriota
Actinobacteriota
Bacteroidota
Bdellovibrionota
Campylobacterota
Chloroflexota
Cyanobacteriota
Deinococcota
Firmicutes
Firmicutes_A
Firmicutes_C
Fusobacteriota
Patescibacteria
Planctomycetota
Proteobacteria
Verrucomicrobiota

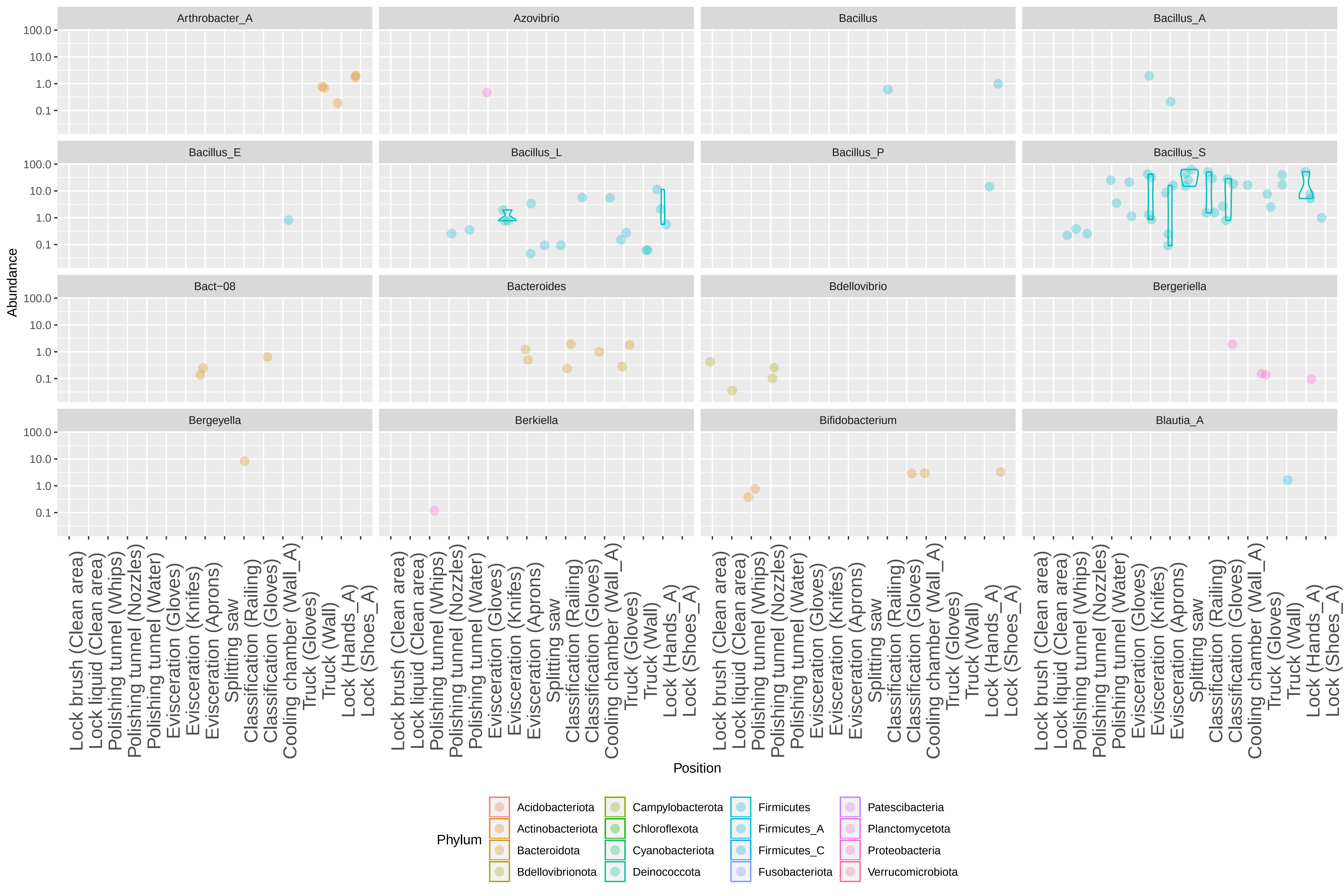

Arthrobacter_A
Azovibrio
Bacillus
Bacillus_A
Bacillus_E
Bacillus_L
Bacillus_P
Bacillus_S
Bact−08
Bacteroides
Bdellovibrio
Bergeriella
Bergeyella
Berkiella
Bifidobacterium
Blautia_A
100.0
10.0
1.0
0.1
Abundance
Lock brush (Clean area)
Lock liquid (Clean area)
Polishing tunnel (Whips)
Polishing tunnel (Nozzles)
Polishing tunnel (Water)
Evisceration (Gloves)
Evisceration (Knifes)
Evisceration (Aprons)
Splitting saw
Classification (Railing)
Classification (Gloves)
Cooling chamber (Wall_A)
Truck (Gloves)
Truck (Wall)
Lock (Hands_A)
Lock (Shoes_A)
Position
Phylum
Acidobacteriota
Actinobacteriota
Bacteroidota
Bdellovibrionota
Campylobacterota
Chloroflexota
Cyanobacteriota
Deinococcota
Firmicutes
Firmicutes_A
Firmicutes_C
Fusobacteriota
Patescibacteria
Planctomycetota
Proteobacteria
Verrucomicrobiota

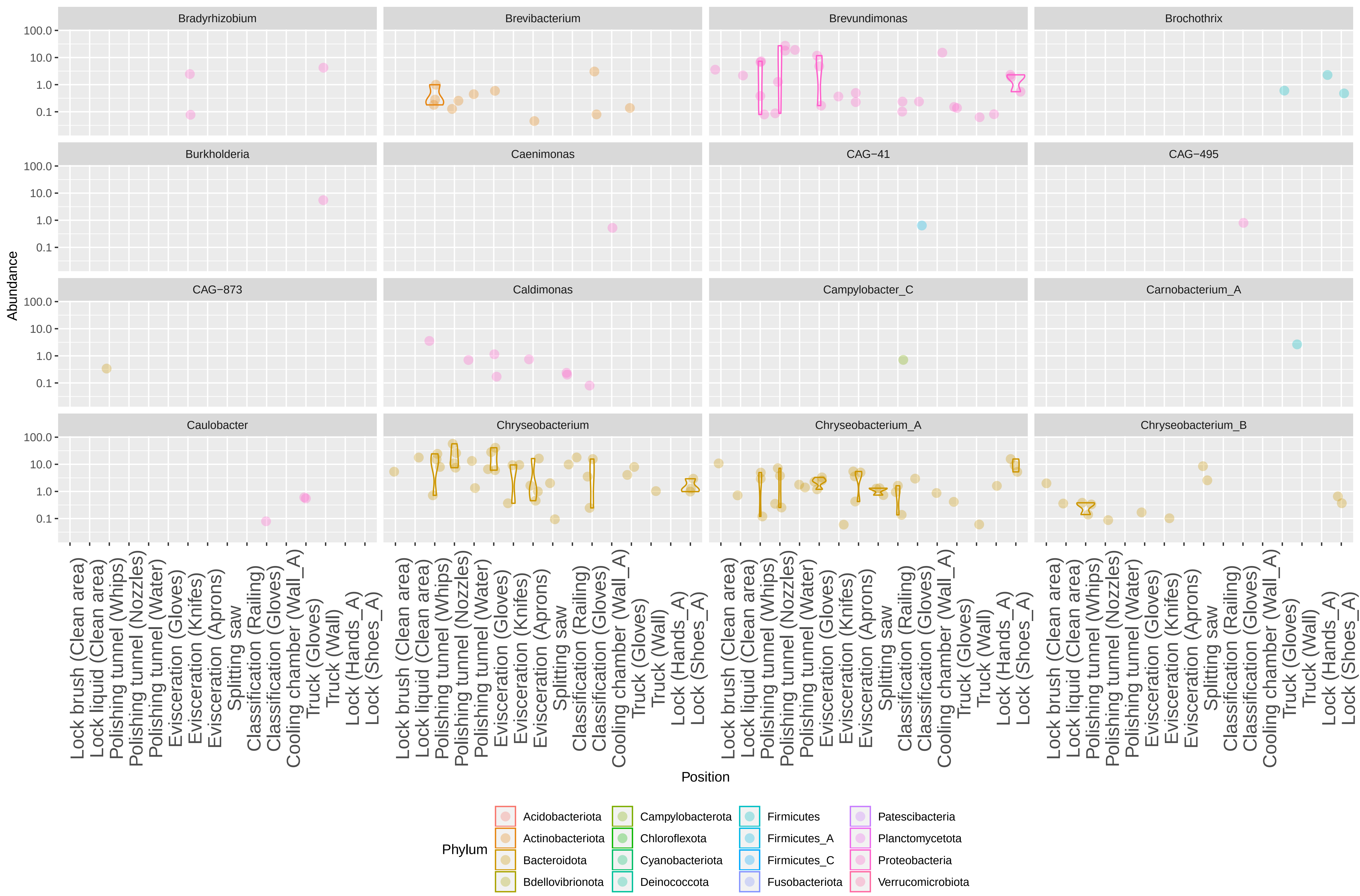

Bradyrhizobium
Brevibacterium
Brevundimonas
Brochothrix
Burkholderia
Caenimonas
CAG−41
CAG−495
CAG−873
Caldimonas
Campylobacter_C
Carnobacterium_A
Caulobacter
Chryseobacterium
Chryseobacterium_A
Chryseobacterium_B
100.0
10.0
1.0
0.1
Abundance
Lock brush (Clean area)
Lock liquid (Clean area)
Polishing tunnel (Whips)
Polishing tunnel (Nozzles)
Polishing tunnel (Water)
Evisceration (Gloves)
Evisceration (Knifes)
Evisceration (Aprons)
Splitting saw
Classification (Railing)
Classification (Gloves)
Cooling chamber (Wall_A)
Truck (Gloves)
Truck (Wall)
Lock (Hands_A)
Lock (Shoes_A)
Position
Phylum
Acidobacteriota
Actinobacteriota
Bacteroidota
Bdellovibrionota
Campylobacterota
Chloroflexota
Cyanobacteriota
Deinococcota
Firmicutes
Firmicutes_A
Firmicutes_C
Fusobacteriota
Patescibacteria
Planctomycetota
Proteobacteria
Verrucomicrobiota

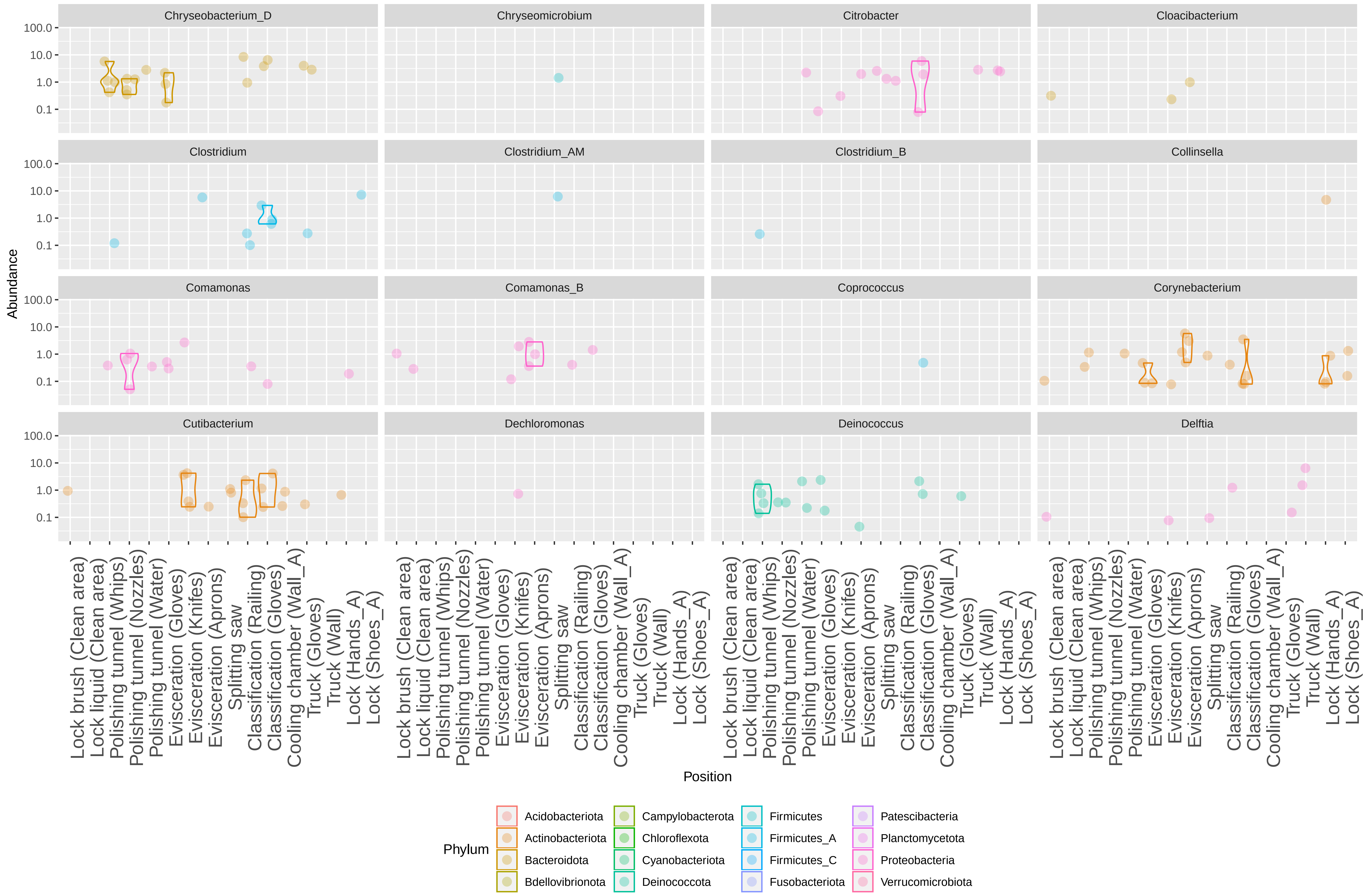

Chryseobacterium_D
Chryseomicrobium
Citrobacter
Cloacibacterium
Clostridium
Clostridium_AM
Clostridium_B
Collinsella
Comamonas
Comamonas_B
Coprococcus
Corynebacterium
Cutibacterium
Dechloromonas
Deinococcus
Delftia
100.0
10.0
1.0
0.1
Abundance
Lock brush (Clean area)
Lock liquid (Clean area)
Polishing tunnel (Whips)
Polishing tunnel (Nozzles)
Polishing tunnel (Water)
Evisceration (Gloves)
Evisceration (Knifes)
Evisceration (Aprons)
Splitting saw
Classification (Railing)
Classification (Gloves)
Cooling chamber (Wall_A)
Truck (Gloves)
Truck (Wall)
Lock (Hands_A)
Lock (Shoes_A)
Position
Phylum
Acidobacteriota
Actinobacteriota
Bacteroidota
Bdellovibrionota
Campylobacterota
Chloroflexota
Cyanobacteriota
Deinococcota
Firmicutes
Firmicutes_A
Firmicutes_C
Fusobacteriota
Patescibacteria
Planctomycetota
Proteobacteria
Verrucomicrobiota

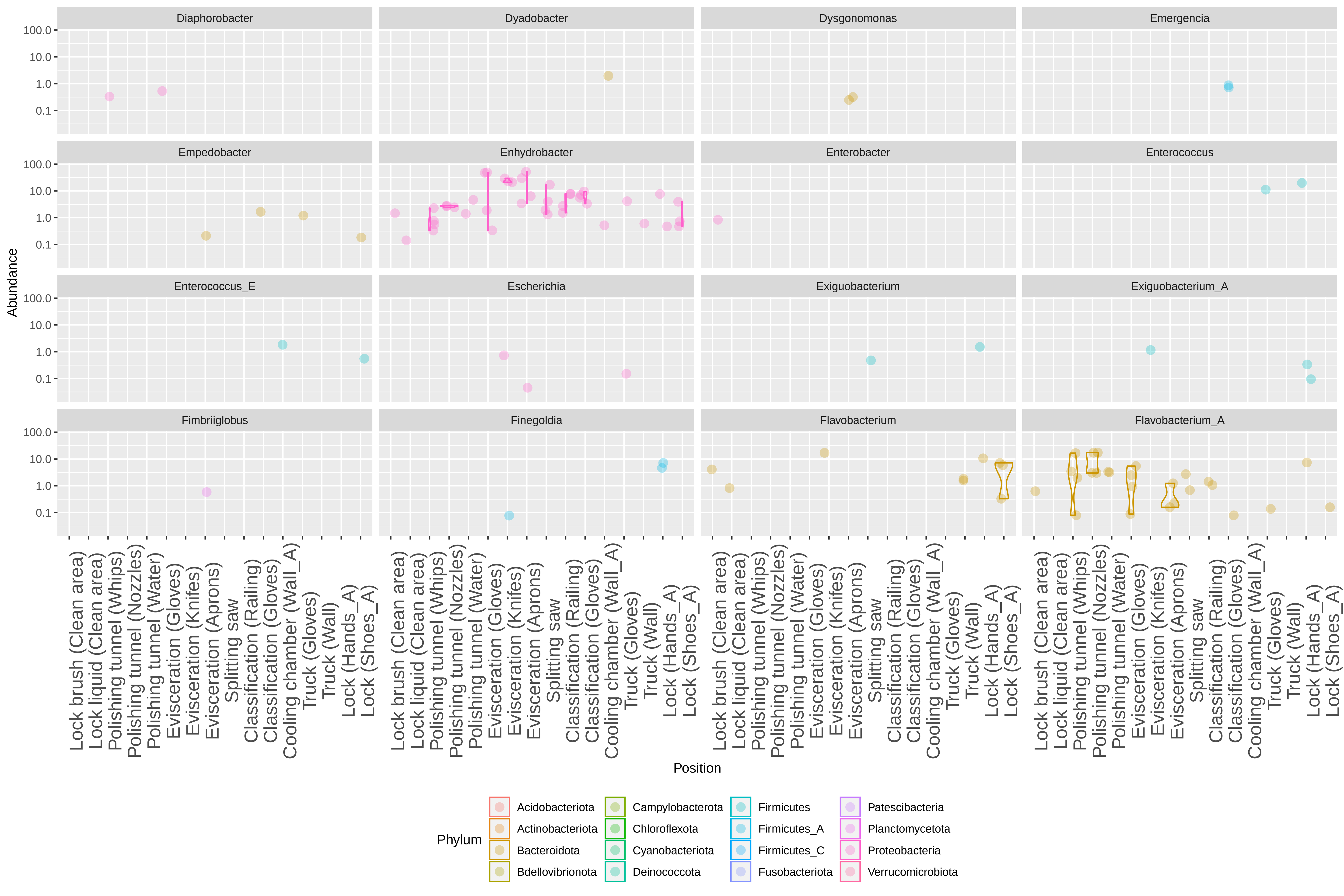

Diaphorobacter
Dyadobacter
Dysgonomonas
Emergencia
Empedobacter
Enhydrobacter
Enterobacter
Enterococcus
Enterococcus_E
Escherichia
Exiguobacterium
Exiguobacterium_A
Fimbriiglobus
Finegoldia
Flavobacterium
Flavobacterium_A
Abundance
100.0
10.0
1.0
0.1
Lock brush (Clean area)
Lock liquid (Clean area)
Polishing tunnel (Whips)
Polishing tunnel (Nozzles)
Polishing tunnel (Water)
Evisceration (Gloves)
Evisceration (Knifes)
Evisceration (Aprons)
Splitting saw
Classification (Railing)
Classification (Gloves)
Cooling chamber (Wall_A)
Truck (Gloves)
Truck (Wall)
Lock (Hands_A)
Lock (Shoes_A)
Position
Phylum
Acidobacteriota
Actinobacteriota
Bacteroidota
Bdellovibrionota
Campylobacterota
Chloroflexota
Cyanobacteriota
Deinococcota
Firmicutes
Firmicutes_A
Firmicutes_C
Fusobacteriota
Patescibacteria
Planctomycetota
Proteobacteria
Verrucomicrobiota

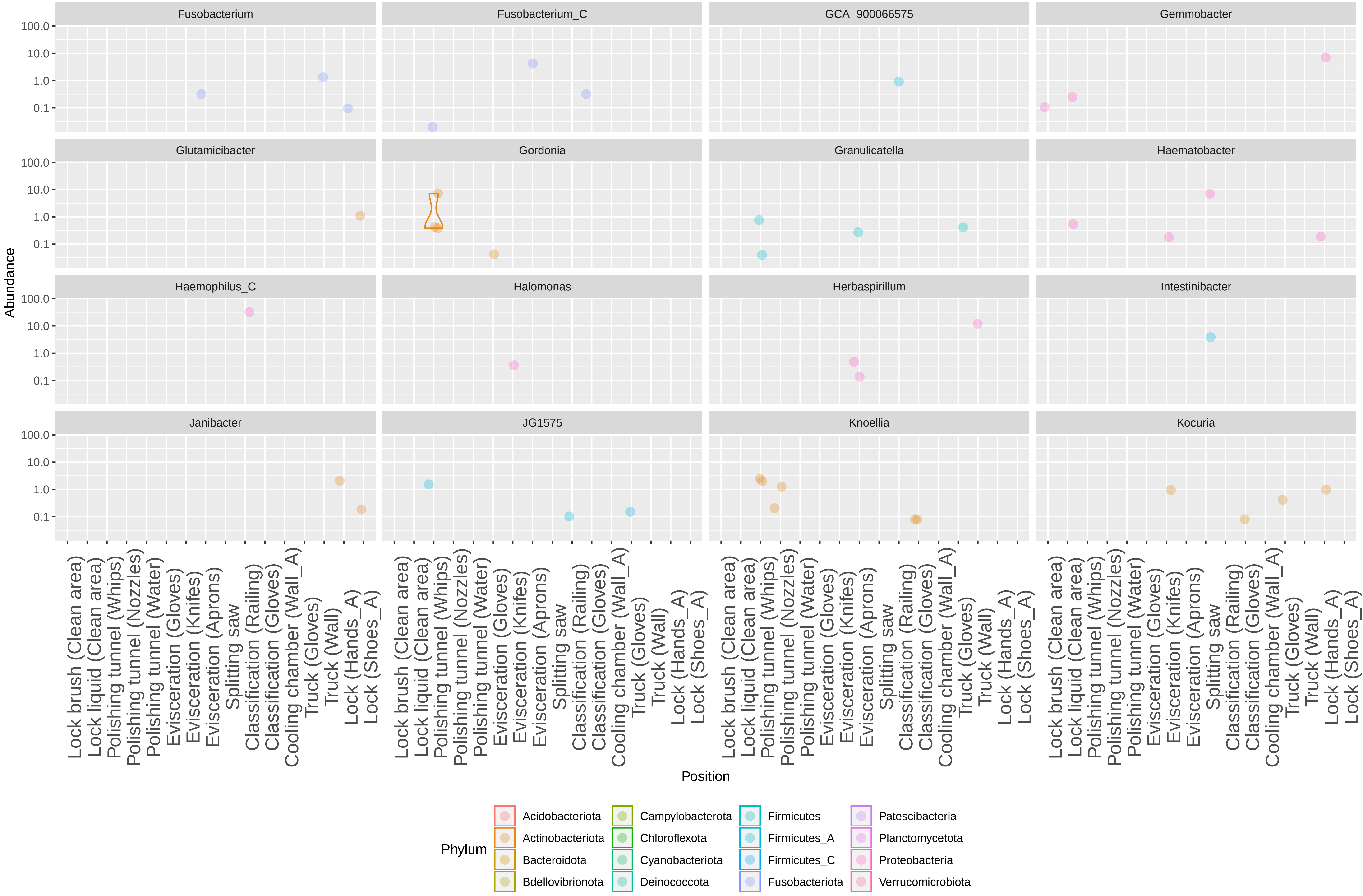

Fusobacterium
Fusobacterium_C
GCA−900066575
Gemmobacter
Glutamicibacter
Gordonia
Granulicatella
Haematobacter
Haemophilus_C
Halomonas
Herbaspirillum
Intestinibacter
Janibacter
JG1575
Knoellia
Kocuria
Abundance
100.0
10.0
1.0
0.1
Position
Lock brush (Clean area)
Lock liquid (Clean area)
Polishing tunnel (Whips)
Polishing tunnel (Nozzles)
Polishing tunnel (Water)
Evisceration (Gloves)
Evisceration (Knifes)
Evisceration (Aprons)
Splitting saw
Classification (Railing)
Classification (Gloves)
Cooling chamber (Wall_A)
Truck (Gloves)
Truck (Wall)
Lock (Hands_A)
Lock (Shoes_A)
Phylum
Acidobacteriota
Actinobacteriota
Bacteroidota
Bdellovibrionota
Campylobacterota
Chloroflexota
Cyanobacteriota
Deinococcota
Firmicutes
Firmicutes_A
Firmicutes_C
Fusobacteriota
Patescibacteria
Planctomycetota
Proteobacteria
Verrucomicrobiota

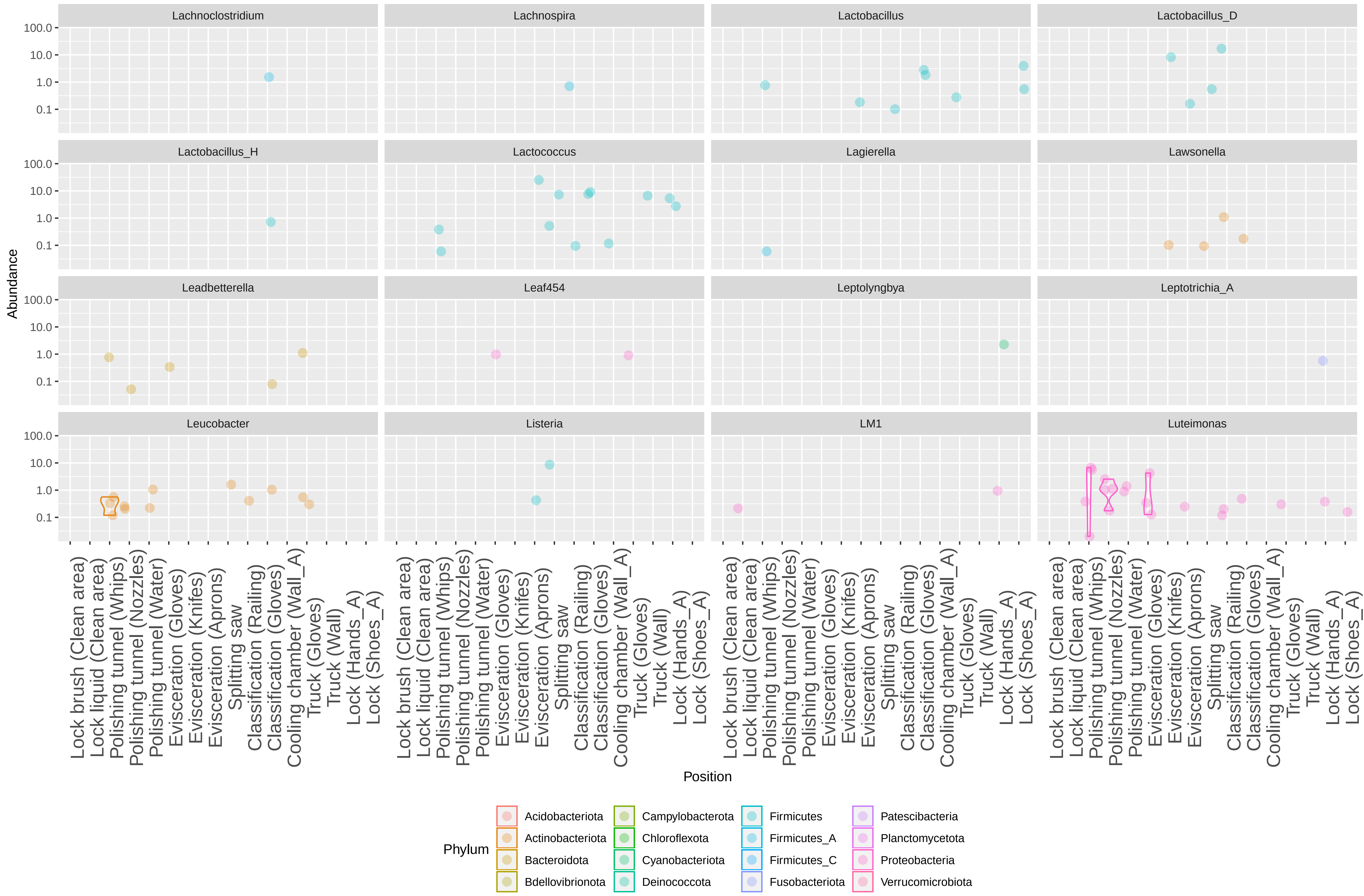

Lachnoclostridium
Lachnospira
Lactobacillus
Lactobacillus_D
Lactobacillus_H
Lactococcus
Lagierella
Lawsonella
Leadbetterella
Leaf454
Leptolyngbya
Leptotrichia_A
Leucobacter
Listeria
LM1
Luteimonas
Abundance
100.0
10.0
1.0
0.1
Position
Lock brush (Clean area)
Lock liquid (Clean area)
Polishing tunnel (Whips)
Polishing tunnel (Nozzles)
Polishing tunnel (Water)
Evisceration (Gloves)
Evisceration (Knifes)
Evisceration (Aprons)
Splitting saw
Classification (Railing)
Classification (Gloves)
Cooling chamber (Wall_A)
Truck (Gloves)
Truck (Wall)
Lock (Hands_A)
Lock (Shoes_A)
Phylum
Acidobacteriota
Actinobacteriota
Bacteroidota
Bdellovibrionota
Campylobacterota
Chloroflexota
Cyanobacteriota
Deinococcota
Firmicutes
Firmicutes_A
Firmicutes_C
Fusobacteriota
Patescibacteria
Planctomycetota
Proteobacteria
Verrucomicrobiota

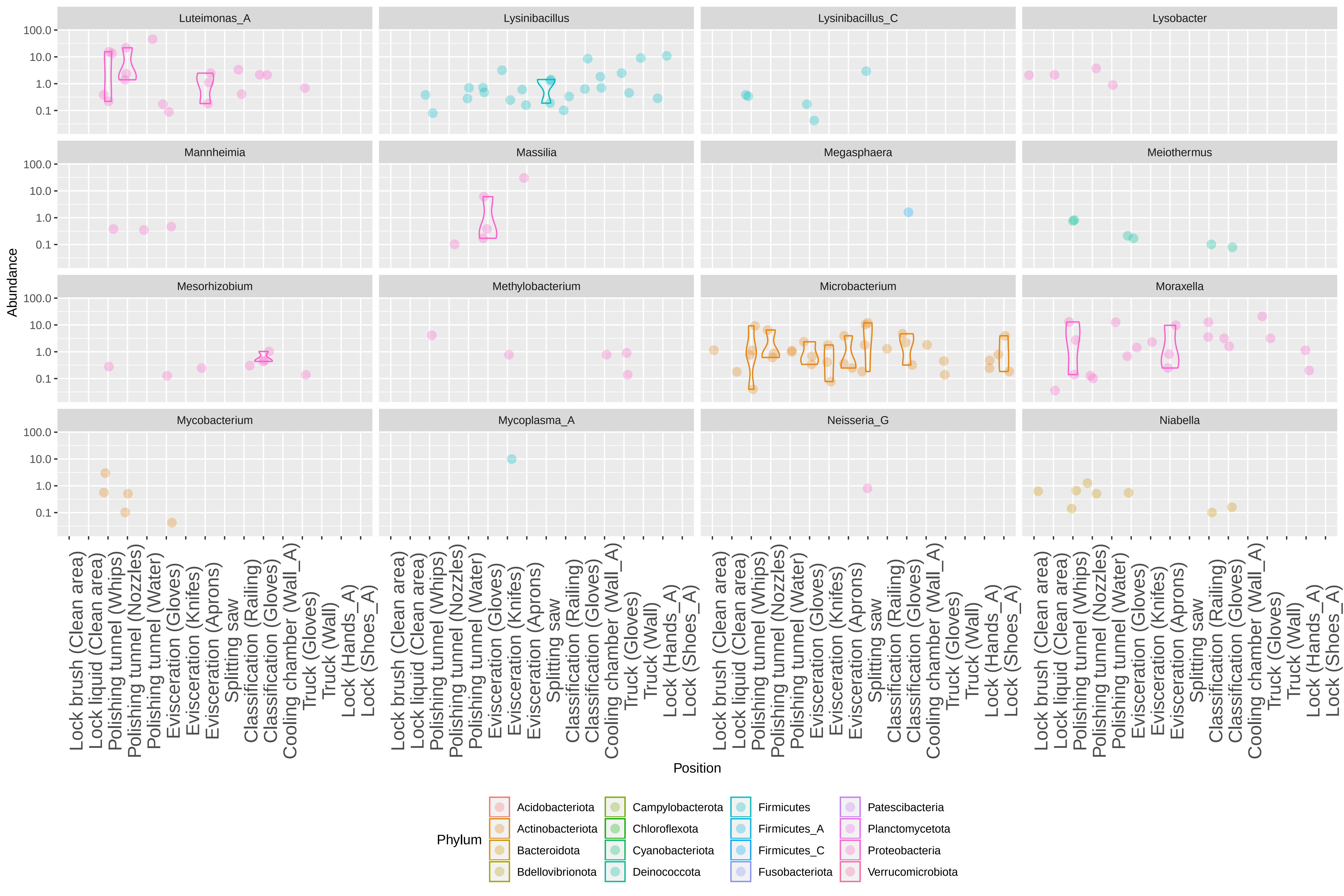
Luteimonas_A
Lysinibacillus
Lysinibacillus_C
Lysobacter
Mannheimia
Massilia
Megasphaera
Meiothermus
Mesorhizobium
Methylobacterium
Microbacterium
Moraxella
Mycobacterium
Mycoplasma_A
Neisseria_G
Niabella
Abundance
100.0
10.0
1.0
0.1
Lock brush (Clean area)
Lock liquid (Clean area)
Polishing tunnel (Whips)
Polishing tunnel (Nozzles)
Polishing tunnel (Water)
Evisceration (Gloves)
Evisceration (Knifes)
Evisceration (Aprons)
Splitting saw
Classification (Railing)
Classification (Gloves)
Cooling chamber (Wall_A)
Truck (Gloves)
Truck (Wall)
Lock (Hands_A)
Lock (Shoes_A)
Position
Phylum
Acidobacteriota
Actinobacteriota
Bacteroidota
Bdellovibrionota
Campylobacterota
Chloroflexota
Cyanobacteriota
Deinococcota
Firmicutes
Firmicutes_A
Firmicutes_C
Fusobacteriota
Patescibacteria
Planctomycetota
Proteobacteria
Verrucomicrobiota

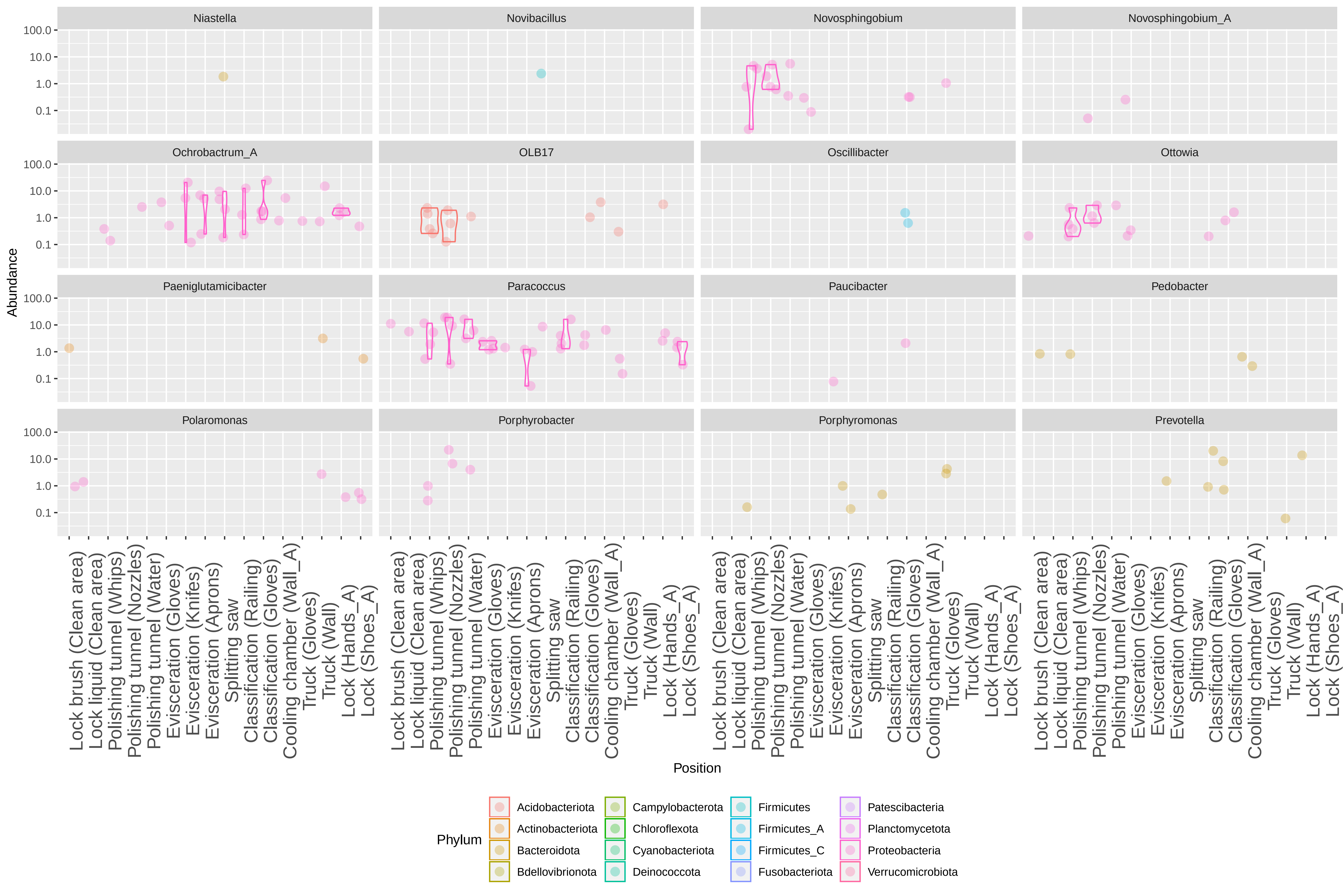

Niastella
Novibacillus
Novosphingobium
Novosphingobium_A
Ochrobactrum_A
OLB17
Oscillibacter
Ottowia
Paeniglutamicibacter
Paracoccus
Paucibacter
Pedobacter
Polaromonas
Porphyrobacter
Porphyromonas
Prevotella
Abundance
100.0
10.0
1.0
0.1
Position
Lock brush (Clean area)
Lock liquid (Clean area)
Polishing tunnel (Whips)
Polishing tunnel (Nozzles)
Polishing tunnel (Water)
Evisceration (Gloves)
Evisceration (Knifes)
Evisceration (Aprons)
Splitting saw
Classification (Railing)
Classification (Gloves)
Cooling chamber (Wall_A)
Truck (Gloves)
Truck (Wall)
Lock (Hands_A)
Lock (Shoes_A)
Phylum
Acidobacteriota
Actinobacteriota
Bacteroidota
Bdellovibrionota
Campylobacterota
Chloroflexota
Cyanobacteriota
Deinococcota
Firmicutes
Firmicutes_A
Firmicutes_C
Fusobacteriota
Patescibacteria
Planctomycetota
Proteobacteria
Verrucomicrobiota

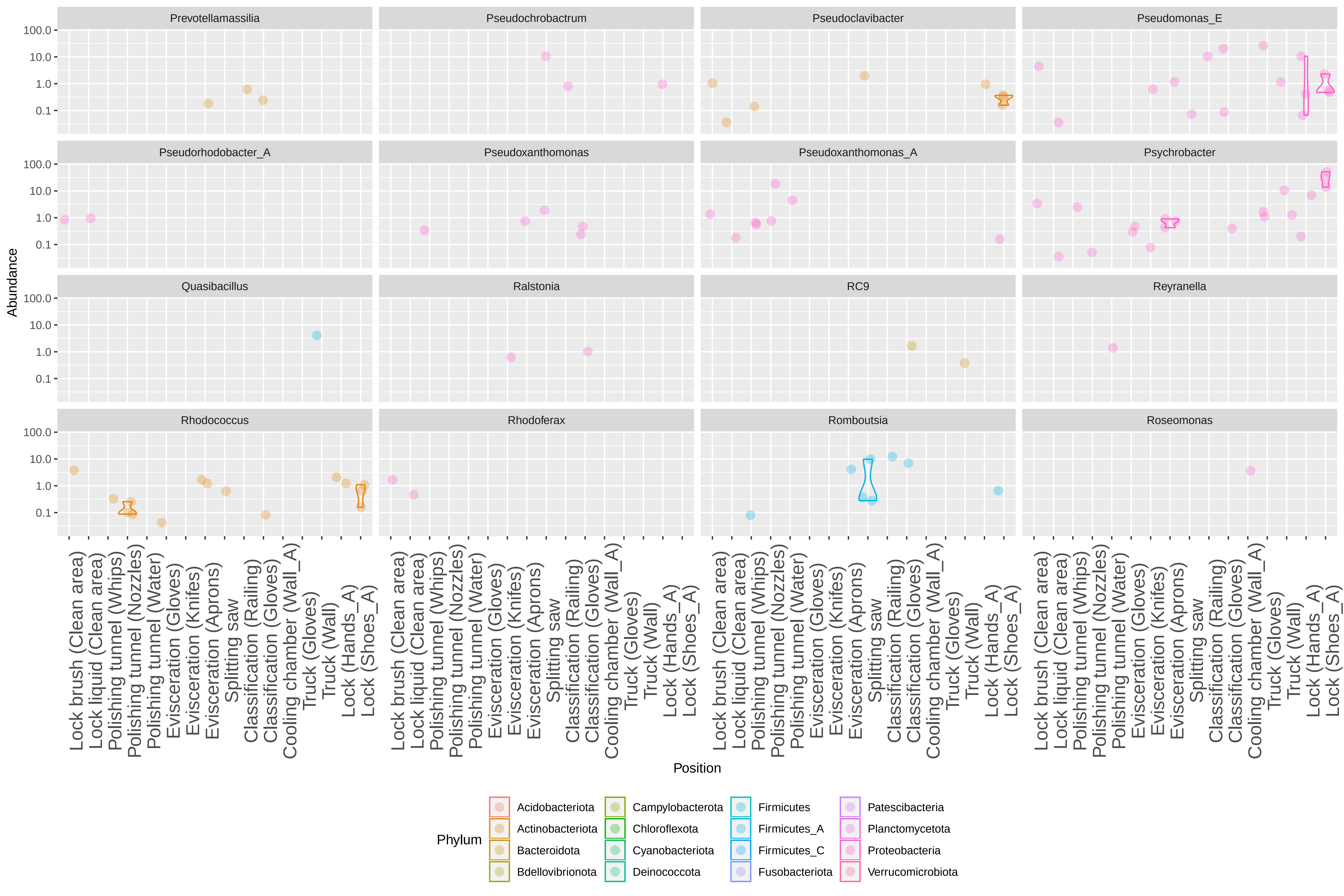

Prevotellamassilia
Pseudochrobactrum
Pseudoclavibacter
Pseudomonas_E
Pseudorhodobacter_A
Pseudoxanthomonas
Pseudoxanthomonas_A
Psychrobacter
Quasibacillus
Ralstonia
RC9
Reyranella
Rhodococcus
Rhodoferax
Romboutsia
Roseomonas
Abundance
100.0
10.0
1.0
0.1
Lock brush (Clean area)
Lock liquid (Clean area)
Polishing tunnel (Whips)
Polishing tunnel (Nozzles)
Polishing tunnel (Water)
Evisceration (Gloves)
Evisceration (Knifes)
Evisceration (Aprons)
Splitting saw
Classification (Railing)
Classification (Gloves)
Cooling chamber (Wall_A)
Truck (Gloves)
Truck (Wall)
Lock (Hands_A)
Lock (Shoes_A)
Position
Phylum
Acidobacteriota
Actinobacteriota
Bacteroidota
Bdellovibrionota
Campylobacterota
Chloroflexota
Cyanobacteriota
Deinococcota
Firmicutes
Firmicutes_A
Firmicutes_C
Fusobacteriota
Patescibacteria
Planctomycetota
Proteobacteria
Verrucomicrobiota

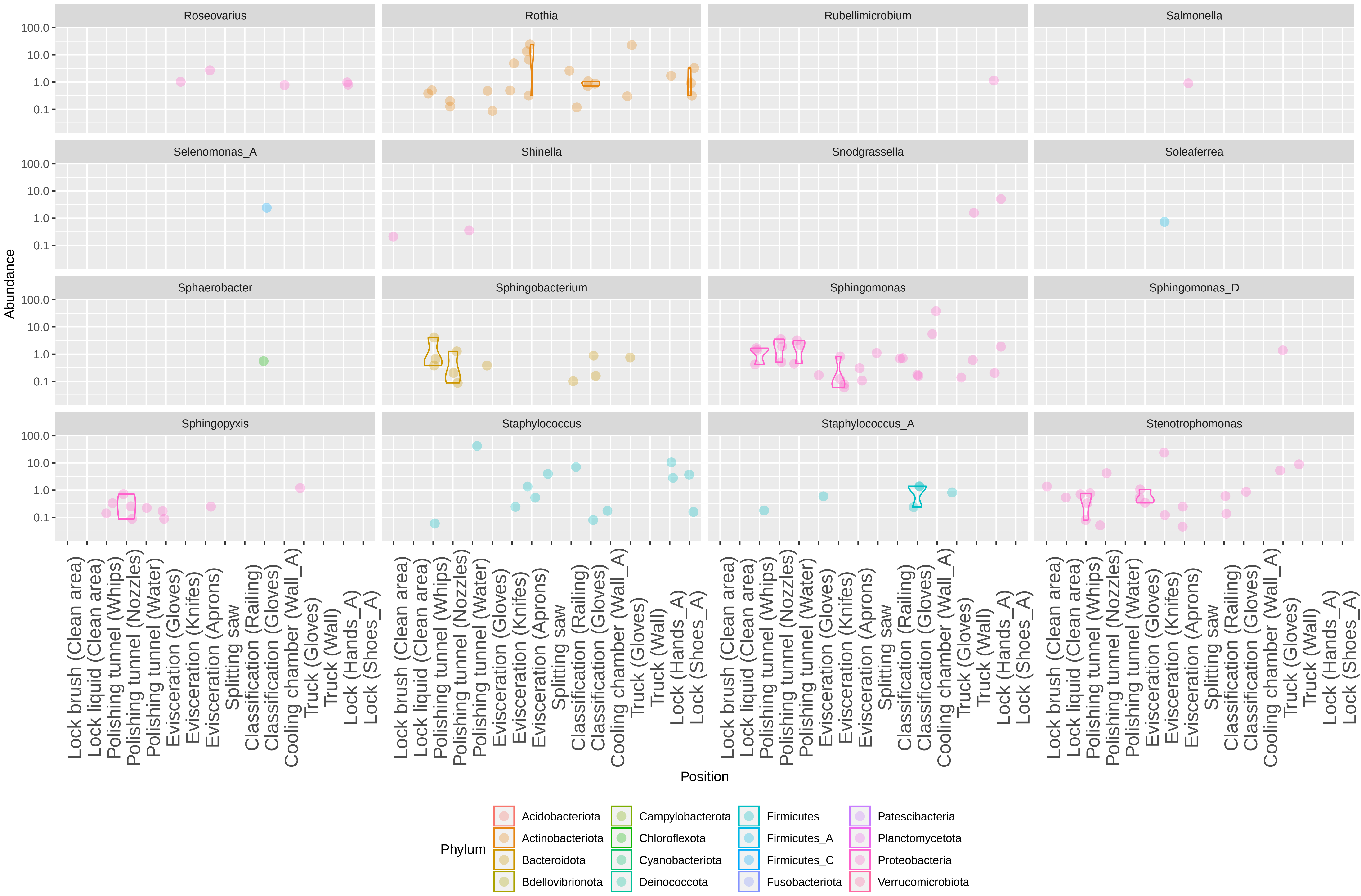
Roseovarius
Rothia
Rubellimicrobium
Salmonella
Selenomonas_A
Shinella
Snodgrassella
Soleaferrea
Sphaerobacter
Sphingobacterium
Sphingomonas
Sphingomonas_D
Sphingopyxis
Staphylococcus
Staphylococcus_A
Stenotrophomonas
Abundance
100.0
10.0
1.0
0.1
Position
Lock brush (Clean area)
Lock liquid (Clean area)
Polishing tunnel (Whips)
Polishing tunnel (Nozzles)
Polishing tunnel (Water)
Evisceration (Gloves)
Evisceration (Knifes)
Evisceration (Aprons)
Splitting saw
Classification (Railing)
Classification (Gloves)
Cooling chamber (Wall_A)
Truck (Gloves)
Truck (Wall)
Lock (Hands_A)
Lock (Shoes_A)
Phylum
Acidobacteriota
Actinobacteriota
Bacteroidota
Bdellovibrionota
Campylobacterota
Chloroflexota
Cyanobacteriota
Deinococcota
Firmicutes
Firmicutes_A
Firmicutes_C
Fusobacteriota
Patescibacteria
Planctomycetota
Proteobacteria
Verrucomicrobiota

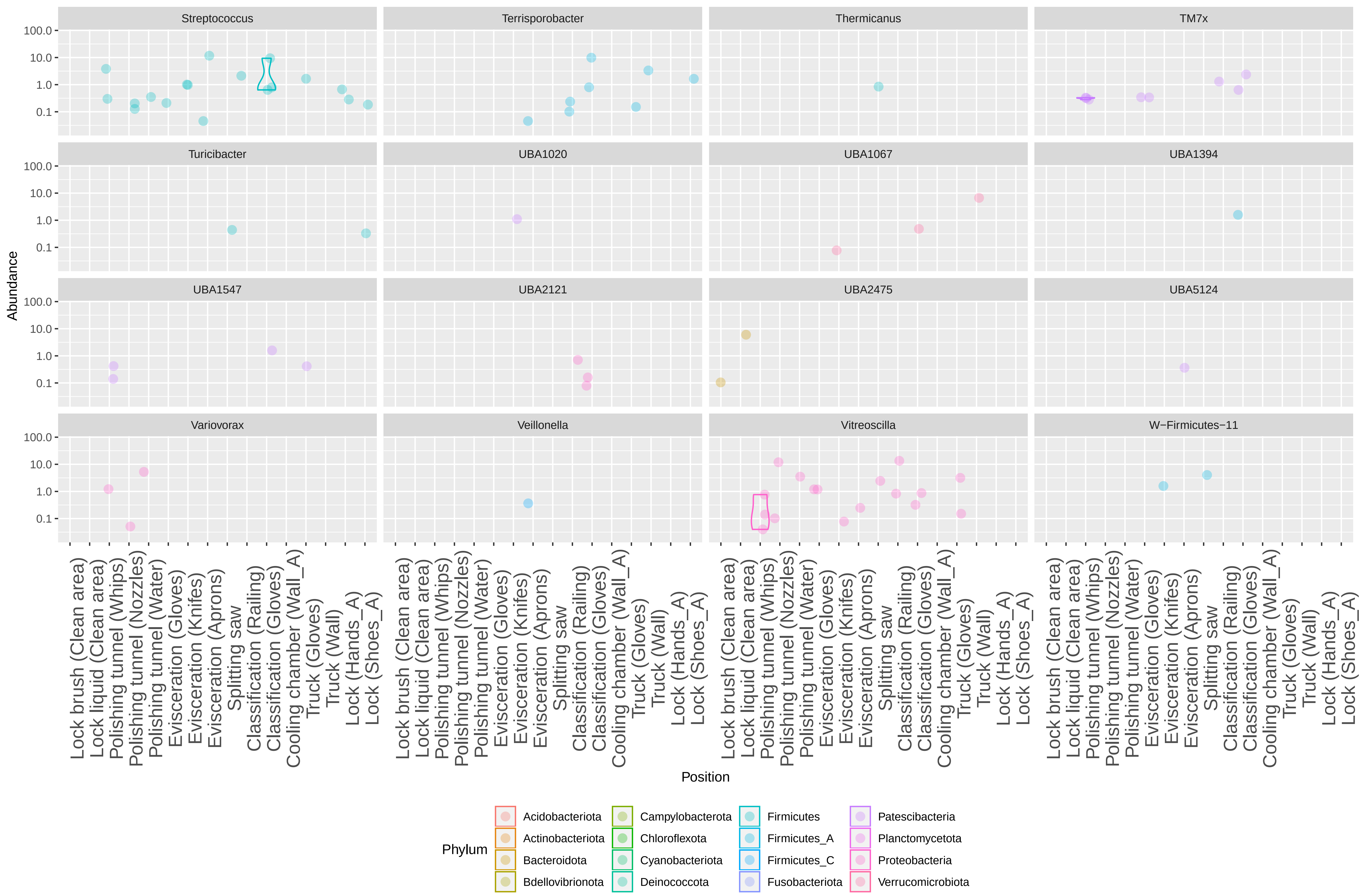

Streptococcus
Terrisporobacter
Thermicanus
TM7x
Turicibacter
UBA1020
UBA1067
UBA1394
UBA1547
UBA2121
UBA2475
UBA5124
Variovorax
Veillonella
Vitreoscilla
W−Firmicutes−11
100.0
10.0
1.0
0.1
Abundance
Lock brush (Clean area)
Lock liquid (Clean area)
Polishing tunnel (Whips)
Polishing tunnel (Nozzles)
Polishing tunnel (Water)
Evisceration (Gloves)
Evisceration (Knifes)
Evisceration (Aprons)
Splitting saw
Classification (Railing)
Classification (Gloves)
Cooling chamber (Wall_A)
Truck (Gloves)
Truck (Wall)
Lock (Hands_A)
Lock (Shoes_A)
Position
Phylum
Acidobacteriota
Actinobacteriota
Bacteroidota
Bdellovibrionota
Campylobacterota
Chloroflexota
Cyanobacteriota
Deinococcota
Firmicutes
Firmicutes_A
Firmicutes_C
Fusobacteriota
Patescibacteria
Planctomycetota
Proteobacteria
Verrucomicrobiota

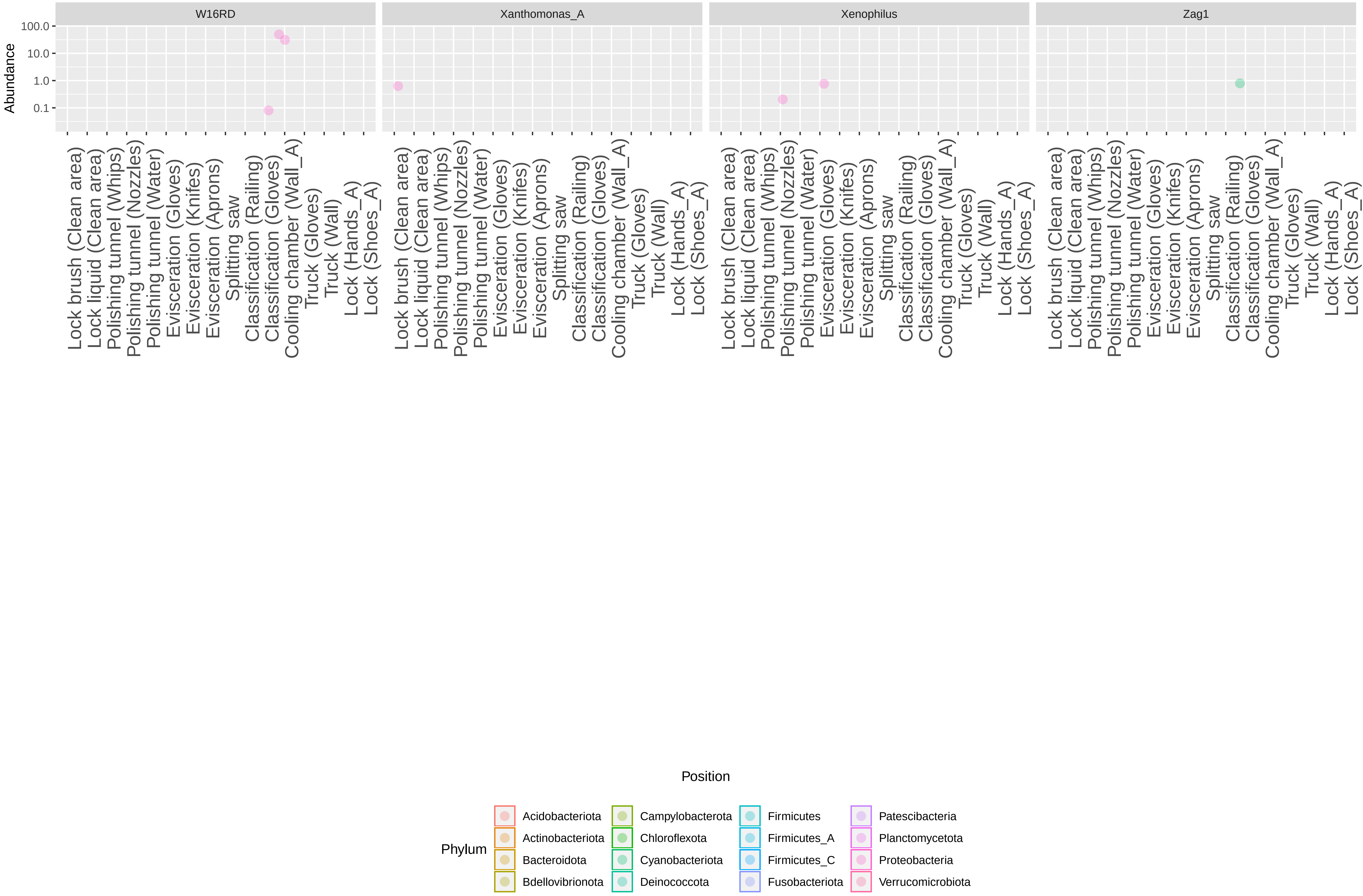

W16RD
Xanthomonas_A
Xenophilus
Zag1
Abundance
100.0
10.0
1.0
0.1
Lock brush (Clean area)
Lock liquid (Clean area)
Polishing tunnel (Whips)
Polishing tunnel (Nozzles)
Polishing tunnel (Water)
Evisceration (Gloves)
Evisceration (Knifes)
Evisceration (Aprons)
Splitting saw
Classification (Railing)
Classification (Gloves)
Cooling chamber (Wall_A)
Truck (Gloves)
Truck (Wall)
Lock (Hands_A)
Lock (Shoes_A)
Position
Phylum
Acidobacteriota
Actinobacteriota
Bacteroidota
Bdellovibrionota
Campylobacterota
Chloroflexota
Cyanobacteriota
Deinococcota
Firmicutes
Firmicutes_A
Firmicutes_C
Fusobacteriota
Patescibacteria
Planctomycetota
Proteobacteria
Verrucomicrobiota

Supplementary figure 5: *Relative abundances of all detected genera across all surface positions.*

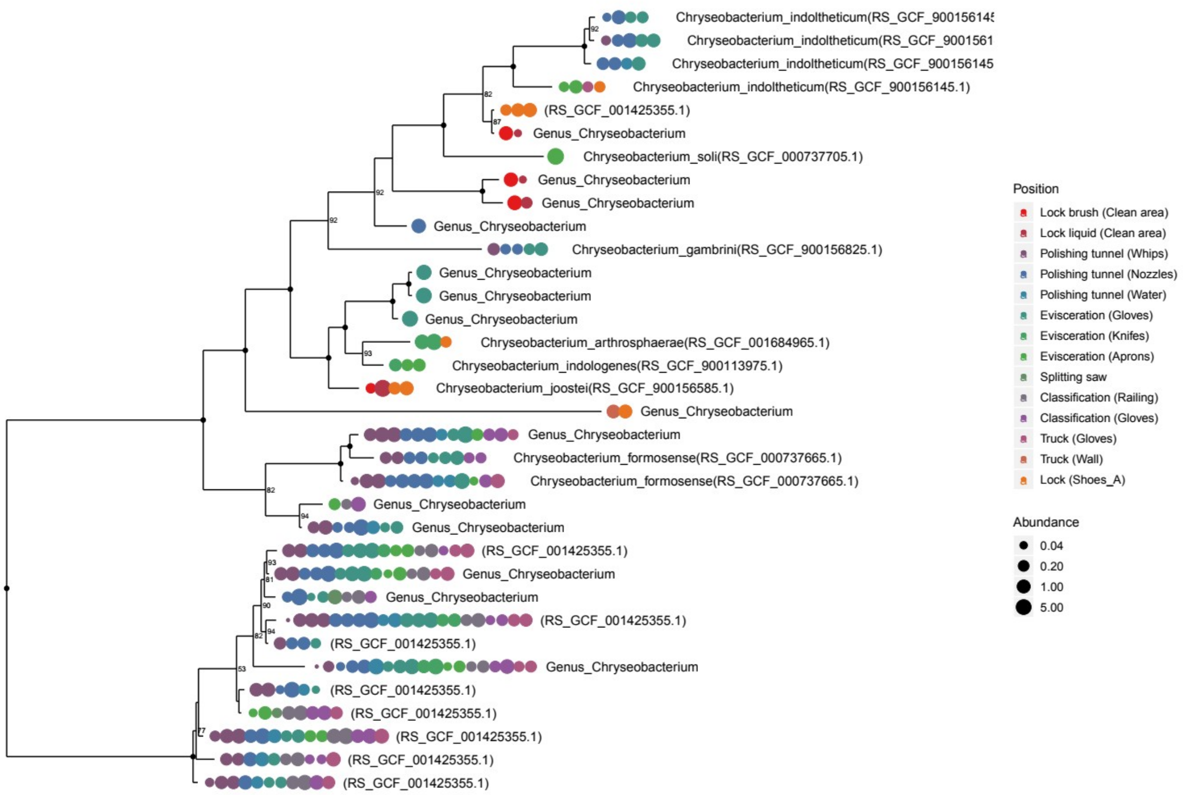

Supplementary Figure 6: *Phylogenetic tree of 16S rRNA gene sequences associated to the genus Chryseobacterium. The size of the circles at the tips of the tree indicate the relative abundance at the respective position illustrated by the colours.*

Supplementary Table 2: *Mean squared differences, mean hit ratios, mean unknown classification rates and mean differences of unknown classification rates compared to the original dataset, stratified by the different dataset size. N.B. All presented means in the table were calculated as the difference between the randomly generated datasets and the original dataset.*

	**Goodness of fit**	**Goodness of classification**		
Dataset size	Mean squared differences x10^-4 (95% CI)	Mean hit ratio % (95% CI)	Mean unknown classification rate % (95% CI)	Mean difference of unknown classification rate % (95% CI)
200	32.7 (26.5 - 38.9)	53.6 (51.5 - 55.7)	45.4 (43.8 - 47.1)	18.2 (17.2 - 19.1)
500	7.9 (5.1 - 10.7)	77.5 (76.2 - 78.7)	36.4 (32.5 - 40.3)	9.1 (8.3 - 9.9)
1000	2.7 (1.8 - 3.5)	85.7 (83.1 - 88.3)	31 (27.1 - 34.9)	3.8 (2.9 - 4.6)
5000	1.4 (1.0 - 1.9)	89.2 (87.5 - 91.0)	29 (25.1 - 32.9)	1.8 (1.2 - 2.5)
7712	1.6 (0.8 – 2.5)	89.8 (86.8 – 92.8)	28.4 (24.7 – 32.1)	1.5 (1.0 - 1.9)
original dataset	0	100	27.2 (17.5 - 37.0)	0

Supplementary Table 3: *Results of the Dunn tests after a significant Kruskal-Wallis rank sum test for mean squared differences, hit ratio and unknown classification rate, whereby the original dataset was used as reference. Significant p-values are highlighted in bold.*

Dataset size		200	500	1000	5000
500	*mean squared differences*	**0.0000**			
	hit ratio	**0.0003**			
	unknown classification	**0.0007**			
1000	*mean squared differences*	**0.0000**	0.0508		
	hit ratio	**0.0000**	0.0629		
	unknown classification	**0.0000**	0.0658		
5000	*mean squared differences*	**0.0000**	**0.0034**	0.1583	
	hit ratio	**0.0000**	**0.0079**	0.2083	
	unknown classification	**0.0000**	**0.0034**	0.1167	
7712	*mean squared differences*	**0.0000**	**0.0010**	0.0859	0.3385
	hit ratio	**0.0000**	**0.0049**	0.1683	0.4139
	unknown classification	**0.0000**	**0.0011**	0.0735	0.3528

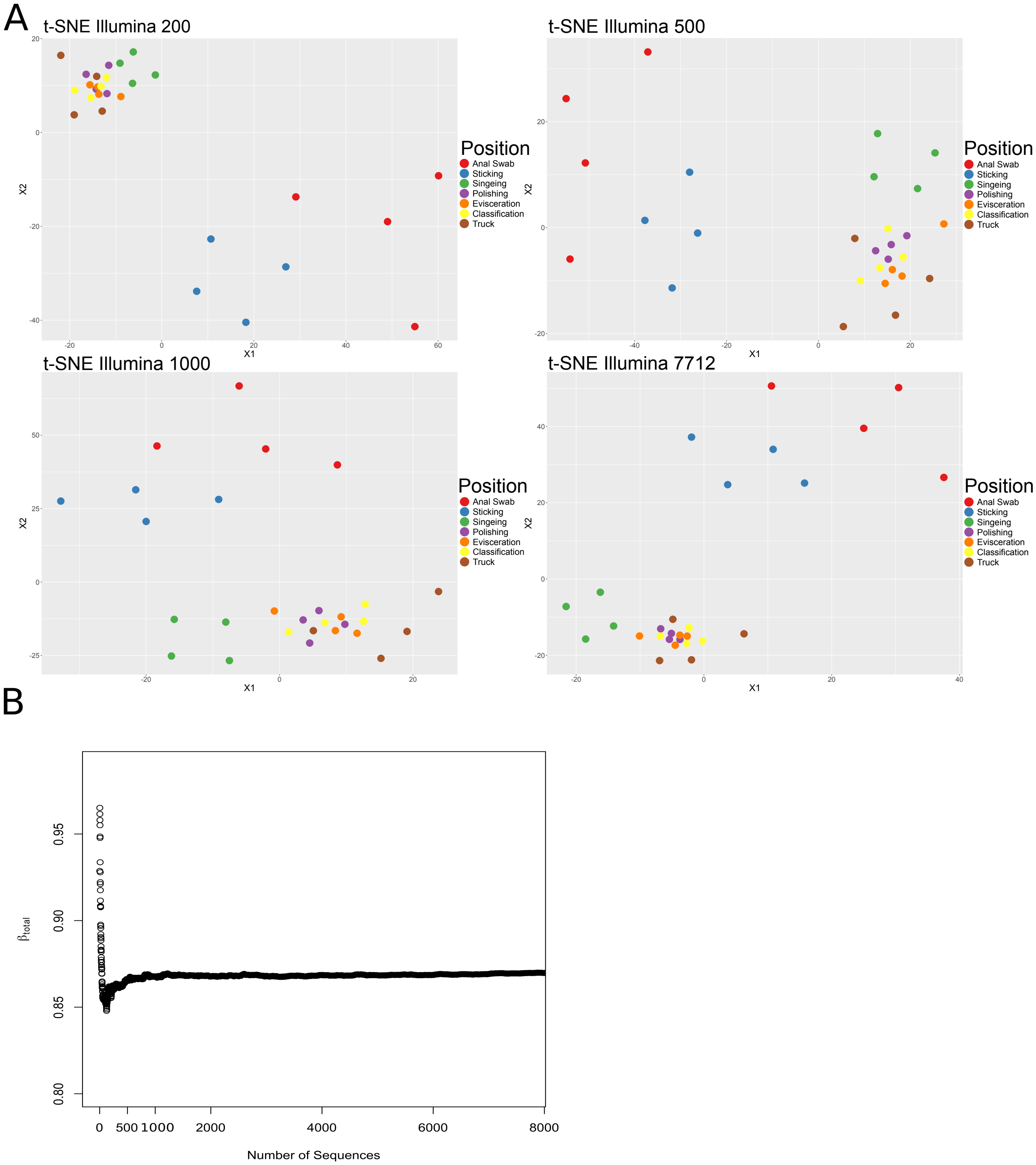

Supplementary Figure 7: *A) t-SNE plots of Bray-Curtis distances based on 16S rRNA gene libraries of the Illumina datasets rarefied to 200, 500, 1,000, and 7,712 sequences per sample. Each point represents values from individual libraries with colors expressing meat samples from different positions along the processing line. B) Accumulation curve for the beta diversity values provided by the function beta.accum of the R package BAT.*

Supplementary Table 4: *Detailed list of samples taken at the slaughterhouse and type of analysis performed*

Detailed list of samples taken at the slaughterhouse and type of analysis performed

Meat

SampleID	Sampling position	Farmer	Plate counts	qPCR	Pacbio	Illumina
GTOE 1	Sticking	Farmer A	x	x	x	
GTOE 2	Sticking	Farmer A	x	x	x	
GTOE 3	Sticking	Farmer A	x	x	x	x
GTOE 4	Sticking	Farmer A	x	x	x	x
GTOE 5	Sticking	Farmer B	x	x	x	x
GTOE 6	Sticking	Farmer B	x	x	x	
GTOE 7	Sticking	Farmer B	x	x	x	
GTOE 8	Sticking	Farmer B	x	x	x	
GTOE 9	Sticking	Farmer C	x	x	x	x
GTOE 10	Sticking	Farmer C	x	x	x	
GTOE 11	Sticking	Farmer C	x	x	x	
GTOE 12	Sticking	Farmer C	x	x	x	
K 1	Anal Swab	Farmer A	x	x	x	
K 2	Anal Swab	Farmer A	x	x	x	
K 3	Anal Swab	Farmer A	x	x	x	x
K 4	Anal Swab	Farmer A	x	x	x	x
K 5	Anal Swab	Farmer B	x	x	x	x
K 6	Anal Swab	Farmer B	x	x	x	
K 7	Anal Swab	Farmer B	x	x	x	
K 8	Anal Swab	Farmer B	x	x	x	
K 9	Anal Swab	Farmer C	x	x	x	x
K 10	Anal Swab	Farmer C	x	x	x	
K 11	Anal Swab	Farmer C	x	x	x	
K 12	Anal Swab	Farmer C	x	x	x	
GF 1	After singeing	Farmer A	x	x	x	
GF 2	After singeing	Farmer A	x	x	x	
GF 3	After singeing	Farmer A	x	x	x	x
GF 4	After singeing	Farmer A	x	x	x	x
GF 5	After singeing	Farmer B	x	x	x	x
GF 6	After singeing	Farmer B	x	x	x	
GF 7	After singeing	Farmer B	x	x	x	
GF 8	After singeing	Farmer B	x	x	x	
GF 9	After singeing	Farmer C	x	x	x	x
GF 10	After singeing	Farmer C	x	x	x	
GF 11	After singeing	Farmer C	x	x	x	
GF 12	After singeing	Farmer C	x	x	x	
GW 1	After polishing	Farmer A	x	x	x	
GW 2	After polishing	Farmer A	x	x	x	
GW 3	After polishing	Farmer A	x	x	x	x
GW 4	After polishing	Farmer A	x	x	x	x
GW 5	After polishing	Farmer B	x	x	x	x
GW 6	After polishing	Farmer B	x	x	x	
GW 7	After polishing	Farmer B	x	x	x	
GW 8	After polishing	Farmer B	x	x	x	
GW 9	After polishing	Farmer C	x	x	x	x
GW 10	After polishing	Farmer C	x	x	x	
GW 11	After polishing	Farmer C	x	x	x	
GW 12	After polishing	Farmer C	x	x	x	
GE 1	After Evisceration	Farmer A	x	x	x	
GE 2	After Evisceration	Farmer A	x	x	x	
GE 3	After Evisceration	Farmer A	x	x	x	x
GE 4	After Evisceration	Farmer A	x	x	x	x
GE 5	After Evisceration	Farmer B	x	x	x	x
GE 6	After Evisceration	Farmer B	x	x	x	
GE 7	After Evisceration	Farmer B	x	x	x	
GE 8	After Evisceration	Farmer B	x	x	x	
GE 9	After Evisceration	Farmer C	x	x	x	x
GE 10	After Evisceration	Farmer C	x	x	x	
GE 11	After Evisceration	Farmer C	x	x	x	
GE 12	After Evisceration	Farmer C	x	x	x	
GK 1	After Classification	Farmer A	x	x	x	
GK 2	After Classification	Farmer A	x	x	x	
GK 3	After Classification	Farmer A	x	x	x	x
GK 4	After Classification	Farmer A	x	x	x	x
GK 5	After Classification	Farmer B	x	x	x	x
GK 6	After Classification	Farmer B	x	x	x	
GK 7	After Classification	Farmer B	x	x	x	
GK 8	After Classification	Farmer B	x	x	x	
GK 9	After Classification	Farmer C	x	x	x	x
GK 10	After Classification	Farmer C	x	x	x	
GK 11	After Classification	Farmer C	x	x	x	
GK 12	After Classification	Farmer C	x	x	x	
GT 1*	Transporter	Farmer A				
GT 2	Transporter	Farmer A	x	x	x	
GT 3	Transporter	Farmer A	x	x	x	x
GT 4	Transporter	Farmer A	x	x	x	x
GT 5	Transporter	Farmer B	x	x	x	x
GT 6	Transporter	Farmer B	x	x	x	
GT 7	Transporter	Farmer B	x	x	x	
GT 8	Transporter	Farmer B	x	x	x	
GT 9	Transporter	Farmer C	x	x	x	x
GT 10*	Transporter	Farmer C				
GT 11	Transporter	Farmer C	x	x	x	
GT 12	Transporter	Farmer C	x	x	x	

Surface

SampleID	Sampling position	Plate counts	qPCR	Pacbio	Illumina
UG 1a	Polishing tunnel whips	x	x	x	x
UG 1b	Polishing tunnel whips	x	x	x	
UG 1c	Polishing tunnel whips	x	x	x	x
UG 1d	Polishing tunnel whips	x	x	x	
UG 2a	Polishing tunnel Nozzles	x	x	x	x
UG 2b	Polishing tunnel Nozzles	x	x	x	
UG 2c	Polishing tunnel Nozzles	x	x	x	x
UG 2d	Polishing tunnel Nozzles	x	x	x	
UG 3 a	Polishing tunnel water	x	x	x	x
UG 3b	Polishing tunnel water	x	x	x	
UG 3c	Polishing tunnel water	x	x	x	x
UG 3d	Polishing tunnel water	x	x	x	
UG 4a	Evisceration gloves	x	x	x	x
UG 4b	Evisceration gloves	x	x	x	
UG 4c	Evisceration gloves	x	x	x	x
UG 4d	Evisceration gloves	x	x	x	
UG 5a	Evisceration knifes	x	x	x	x
UG 5b	Evisceration knifes	x	x	x	
UG 5c	Evisceration knifes	x	x	x	x
UG 5d	Evisceration knifes	x	x	x	
UG 6a	Evisceration aprons	x	x	x	x
UG 6b	Evisceration aprons	x	x	x	
UG 6c	Evisceration aprons	x	x	x	x
UG 6d	Evisceration aprons	x	x	x	
UG 7a	Splitting Saw	x	x	x	x
UG 7b	Splitting Saw	x	x	x	
UG 7c	Splitting Saw	x	x	x	x
UG 7d	Splitting Saw	x	x	x	
UG 8a	Classification railing	x	x	x	x
UG 8b	Classification railing	x	x	x	
UG 8c	Classification railing	x	x	x	x
UG 8d	Classification railing	x	x	x	
UG 9a	Classification gloves	x	x	x	x
UG 9b	Classification gloves	x	x	x	
UG 9c	Classification gloves	x	x	x	x
UG 9d	Classification gloves	x	x	x	
UG 10a	Cooling chamber hook system	x	x		
UG 10b	Cooling chamber hook system	x	x		
UG 10c	Cooling chamber hook system	x	x		
UG 10d	Cooling chamber hook system	x	x		
UG 11a	Cooling chamber wall	x	x	x	
UG 11b	Cooling chamber wall	x	x		x
UG 11c	Cooling chamber wall	x	x	x	
UG 11d	Cooling chamber wall	x	x		
UG 12a	Transporter gloves	x	x	x	x
UG 12b	Transporter gloves	x	x	x	
UG 13a	Transporter hook system	x	x		
UG 13b	Transporter hook system	x	x		
UG 14a	Transporter wall	x	x	x	x
UG 14b	Transporter wall	x	x	x	
UG 15a	Lock hands	x	x	x	x
UG 15b	Lock hands	x	x	x	
UG 15c	Lock hands	x	x	x	
UG 15d	Lock hands	x	x		
UG 16a	Lock shoes	x	x	x	
UG 16b	Lock shoes	x	x	x	
UG 16c	Lock shoes	x	x	x	
UG 16d	Lock shoes	x	x		
UG 23a	Air filter pig hall	x	x		
UG 23b	Air filter pig hall	x	x		
UG 24a	Lock Brush (clean area)	x	x	x	x
UG 24b	Lock Brush (clean area)	x	x		
UG 25a	Evisceration hand shower	x	x		
UG 25b	Evisceration hand shower	x	x		
UG 26a	Cooling chamber railing	x	x		
UG 26b	Cooling chamber railing	x	x		
UG 27a	Splitting saw hand shower	x	x		
UG 27b	Splitting saw hand shower	x	x		
UG 28	Lock liquid (clean area)	x	x	x	x
UG 29	Evisceration sterile basin liquid	x	x	x	x
UG 30	Scalding water before recycling	x	x		
UG 31	Grid liquid (clean/dirty area)	x	x		
UG 32	Expedit railing	x	x		
UG 33	Pusher cooling chamber gloves	x	x		
UG 34	Transporter swing door	x	x		

*Could not be sampled because carcass/meat lost tag

Supplementary Table 5: *Details on the PCR setup for the 16S rRNA gene qPCR*

Total bacteria			
**Mastermix components**	**Stock concentration**	**Final concentration**	**µl per PCR tube**
Aqua dest.			9.7
10 × buffer	10 x	1 x	2.5
$MgCl_2$	50 mM	2 mM	1
FP 16S	2.5 µM	0.25 µM	2.5
RP 16S	2.5 µM	0.25 µM	2.5
EvaGreen	50 x	1 x	0.5
dNTP's	5 mM	0.2 mM	1
Platinum Taq Polymerase	5 U/µl	1.5 U	0.3
Total			20
Template (DNA)			5
Reaction volume			25

PCR step	Temperature ° C	Time	Cycles
Initialization	95	3 min	1 x
Denaturation	95	5 sec	40 x
Annealing and extension	60	20 sec	
Melting curve	95	1 min	1 x
	60	30 sec	
	95	30 sec	

Primer sequence	Amplicon size	Reference
FP: 5´-CCTACGGGAGGCAGCAG-3´<br>RP: 5´-ATTACCGCGGCTGCTGG-3´	189	Muyzer, G., de Waal, E

Supplementary Note 1: *R markdown script of the sequence processing and community analysis workflow. Also available at GitHub (github.com/FFoQS90).*

````
---
title: "The sources and transmission routes of microbial populations throughout a meat processing facility"
author: "Benjamin Zwirzitz, Franz-Ferdinand Roch, Beate Pinior"
date: "26 May 2020"
output: html_document
---

```{r setup, include=FALSE}
knitr::opts_chunk$set(echo = TRUE, eval = FALSE)
```

#Libraries
```{r Libraries}
library(dada2)
library(ShortRead)
library(Biostrings)
library(DECIPHER)
library(reshape2)
```
#**Pacbio**
##Data input
```{r Data input}
#Set path to folder in which you have your data
path1 <- "C://Benni/WP1 Pacbio reads neu/pacbio reads/Run1"
path2 <- "C://Benni/WP1 Pacbio reads neu/pacbio reads/Run2"
path3 <- "C://Benni/WP1 Pacbio reads neu/pacbio reads/Run3"

#Check if path is correct and all files are here
list.files(path1)
list.files(path2)
list.files(path3)

#Read in fastq files
fns1 <- list.files(path1, pattern=".fastq", full.names = TRUE)
fns2 <- list.files(path2, pattern=".fastq", full.names = TRUE)
fns3 <- list.files(path3, pattern=".fastq", full.names = TRUE)
```

##Trim primers and quality filter reads
```{r QC}
F27 <- "AGRGTTYGATYMTGGCTCAG" ## forward primer sequence
R1492 <- "RGYTACCTTGTTACGACTT" ## reverse primer sequence
rc <- dada2:::rc

#Remove primers and orient reads
noprimers1 <- file.path(path1, "noprimers1", basename(fns1))
noprimers2 <- file.path(path2, "noprimers2", basename(fns2))
noprimers3 <- file.path(path3, "noprimers3", basename(fns3))
````
````

~~~~
for(i in seq_along(fns1)) {
  fn1 <- fns1[[i]]; nop1 <- noprimers1[[i]]
  dada2:::removePrimers(fn1, nop1, primer.fwd=F27, primer.rev=dada2:::rc(R1492), orient=TRUE)
}

for(i in seq_along(fns2)) {
  fn2 <- fns2[[i]]; nop2 <- noprimers2[[i]]
  dada2:::removePrimers(fn2, nop2, primer.fwd=F27, primer.rev=dada2:::rc(R1492), orient=TRUE)
}

for(i in seq_along(fns3)) {
  fn3 <- fns3[[i]]; nop3 <- noprimers3[[i]]
  dada2:::removePrimers(fn3, nop3, primer.fwd=F27, primer.rev=dada2:::rc(R1492), orient=TRUE)
}

#Inspect length distribution of sequences
lens.fn1 <- lapply(noprimers1, function(fn1) nchar(getSequences(fn1)))
lens1 <- do.call(c, lens.fn1)
hist(lens1, 100)

lens.fn2 <- lapply(noprimers2, function(fn2) nchar(getSequences(fn2)))
lens2 <- do.call(c, lens.fn2)
hist(lens2, 100)

lens.fn3 <- lapply(noprimers3, function(fn3) nchar(getSequences(fn3)))
lens3 <- do.call(c, lens.fn3)
hist(lens3, 100)

#Inspect read quality profiles
plotQualityProfile(noprimers1[1:2])
plotQualityProfile(noprimers2[1:2])
plotQualityProfile(noprimers3[1:2])

#Filter reads
filts1 <- file.path(path1, "noprimers1", "filtered1", basename(fns1))
filtered1 <- filterAndTrim(noprimers1, filts1, minQ=3, minLen=1000, maxLen=1600, maxN=0,
rm.phix=FALSE, maxEE=2)

filts2 <- file.path(path2, "noprimers2", "filtered2", basename(fns2))
filtered2 <- filterAndTrim(noprimers2, filts2, minQ=3, minLen=1000, maxLen=1600, maxN=0,
rm.phix=FALSE, maxEE=2)

filts3 <- file.path(path3, "noprimers3", "filtered3", basename(fns3))
filtered3 <- filterAndTrim(noprimers3, filts3, minQ=3, minLen=1000, maxLen=1600, maxN=0,
rm.phix=FALSE, maxEE=2)
```

##Dereplicate, learn error rates, and run DADA2
```{r DADA2}
#Dereplicate identical reads
derep1 <- derepFastq(filts1, verbose=TRUE)
derep2 <- derepFastq(filts2, verbose=TRUE)
~~~~

````
derep3 <- derepFastq(filts3, verbose=TRUE)

#Learn the error rates
err1 <- learnErrors(derep1, errorEstimationFunction=PacBioErrfun, BAND_SIZE=32,
multithread=TRUE)
err2 <- learnErrors(derep2, errorEstimationFunction=PacBioErrfun, BAND_SIZE=32,
multithread=TRUE)
err3 <- learnErrors(derep3, errorEstimationFunction=PacBioErrfun, BAND_SIZE=32,
multithread=TRUE)

#visualize the estimated error rates:
plotErrors(err1)
plotErrors(err2)
plotErrors(err3)

#We are now ready to apply the core sample inference algorithm to the dereplicated data.
ddpool1 <- dada(derep1, err=err1, pool = TRUE, multithread=TRUE)
ddpool2 <- dada(derep2, err=err2, pool = TRUE, multithread=TRUE)
ddpool3 <- dada(derep3, err=err3, pool = TRUE, multithread=TRUE)

#Renaming sample names to make them the same as in metadata
sample.names1 <- sapply(strsplit(basename(fns1), "_...."), `[`, 2)
sample.names2 <- sapply(strsplit(basename(fns2), "_...."), `[`, 2)
sample.names3 <- sapply(strsplit(basename(fns3), "_...."), `[`, 2)

names(ddpool1) <- sample.names1
names(ddpool2) <- sample.names2
names(ddpool3) <- sample.names3

#Inspecting the returned dada-class object:
ddpool1
ddpool2
ddpool3
```

##Construct sequence table
```{r Construct sequence table}
st1 <- makeSequenceTable(ddpool1)
st2 <- makeSequenceTable(ddpool2)
st3 <- makeSequenceTable(ddpool3)

#Merge multiple runs
seqtab <- mergeSequenceTables(st1, st2, st3)

#Check the number of sequence variants
dim(seqtab)

#Inspect distribution of sequence lengths
meansequencelength <- as.data.frame(table(nchar(getSequences(seqtab))))

#Inspect mean sequence length
dna <- DNAStringSet(getSequences(seqtab))
````

meansequencelength <- as.data.frame(table(nchar(getSequences(dna))))
meansequencelength$Var1 <- as.numeric(as.character(meansequencelength$Var1))
meansequencelength$total <- meansequencelength$Var1*meansequencelength$Freq
sum(meansequencelength$total)/sum(meansequencelength$Freq)

```
##Remove chimeras
```{r Chimera check}
bim <- isBimeraDenovo(seqtab, minFoldParentOverAbundance=3.5)
table(bim)

sum(seqtab[,bim])/sum(seqtab)

seqtab.nochim <- removeBimeraDenovo(seqtab, method="consensus", multithread=TRUE,
verbose=TRUE)

dim(seqtab.nochim)

sum(seqtab.nochim)/sum(seqtab)
```
##Assign taxonomy
```{r Taxonomy}
#Download the silva_nr_v128_train_set.fa.gz file, and place it in the directory with the fastq files.
tax_gtdb <- assignTaxonomy(seqtab.nochim, "C://Benni/GTDB_bac-arc_ssu_r86.fa.gz",
multithread=TRUE)

#Inspect the taxonomic assignments:
taxa.print <- taxa_silva # Removing sequence rownames for display only
rownames(taxa.print) <- NULL
head(taxa.print)

saveRDS(tax_gtdb, "C://Benni/WP1 Pacbio reads neu/tax_gtdb.rds")
```
#Make phylogenetic tree
```{r}
#Generate alignment of sequences for phylogenetic tree generation
seqs <- getSequences(seqtab.nochim)
names(seqs) <- seqs # This propagates to the tip labels of the tree
alignment <- AlignSeqs(DNAStringSet(seqs), anchor=NA)

saveRDS(alignment, "C://Benni/WP1 Pacbio reads neu/alignment.rds")
writeXStringSet(alignment, file="C://Benni/WP1 Pacbio reads neu/alignment.fasta")

#For Pacbio data we have to create shorter tip labels in the alignment otherwise we could not read
the finished tree back into R because the long labels would cause R to crash.

library(seqinr)

#read in alignment
alignment = read.alignment("C://Benni/WP1 Pacbio reads neu/alignment.fasta", "fasta",
forceToLower = TRUE)

~~~
#make mapping file with new names
mapping_file <- as.data.frame(alignment$nam)
colnames(mapping_file) <- "full_id"
mapping_file$new_id <- paste("seq", seq(1, nrow(mapping_file), 1), sep ="")

#write new alignment file
write.fasta(alignment$seq, mapping_file$new_id, file.out = "C://Benni/WP1 Pacbio reads
neu/alignment_renamed.fasta")

#Download the FastTree program at: http://www.microbesonline.org/fasttree/
#Copy the fastTree executable file in your folder with the alignment. fasta file. Then run the
windows command line and go to this folder. To run fastTree type: FastTree -gtr -nt alignment.fasta
> tree_file
#The program calculates the tree and exports it to a file called tree_file. This file can then be
imported back in R.

#Import tree
library(ape)
tree <- read_tree("C://Benni/WP1 Pacbio reads neu/tree_file")
#add fullID to tip labels after import
tree$tip.label <- mapping_file[[1]][match(tree$tip.label, mapping_file[[2]])]
```
#Construct phyloseq object
```{r}
#Load metadata
sample_data <- as.data.frame(read.table('c://Benni/WP1 Pacbio reads neu/metadata.csv',
header=TRUE, sep=";",dec = ","))
#Load taxa and ESV table
seqtab.nochim <- readRDS("C://Benni/WP1 Pacbio reads neu/seqtab.nochim.rds")
tax_gtdb <- readRDS("C://Benni/WP1 Pacbio reads neu/tax_gtdb.rds")

#make levels for metadata factors according to sequential positions throughout production line
sample_data$Position <- factor(sample_data$Position, levels= c("Anal Swab", "Sticking",
"Singeing", "Polishing", "Evisceration", "Classification", "Truck","Lock brush (Clean area)", "Lock
liquid (Clean area)", "Polishing tunnel (Whips)", "Polishing tunnel (Nozzles)", "Polishing tunnel
(Water)", "Evisceration (Knife basin sterile)", "Evisceration (Gloves)", "Evisceration (Knifes)",
"Evisceration (Aprons)", "Splitting saw", "Classification (Railing)", "Classification (Gloves)",
"Cooling chamber (Wall)", "Truck (Gloves)", "Truck (Wall)", "Lock (Hands)", "Lock (Shoes)"))

#Replace dots with underscores and make sure rownames of metadata table are the same as sample
names in seqtab.
sample_data$SampleID <- gsub(".", "_", sample_data$SampleID, fixed=TRUE)
rownames(sample_data) <- sample_data[,1]
rownames(seqtab.nochim) <- gsub(".", "_", rownames(seqtab.nochim), fixed = TRUE)
rownames(seqtab.nochim) %in% sample_data$SampleID

#Combine into phyloseq object
physeq <- phyloseq(otu_table(seqtab.nochim, taxa_are_rows=FALSE),
            sample_data(sample_data),
            tax_table(tax_gtdb),phy_tree(tree))
physeq
```
~~~

````
#**Illumina**
#!!IMPORTANT!!
#Save files from the Pacbio pipeline, then clear global environment before you continue!
##Data input
```{r}
#Set path to folder in which you have your data
path <- "C://Benni/WP1_Illumina"

#Check if path is correct and all files are here
list.files(path)

#Read in forward and reverse fastq files
fnFs <- sort(list.files(path, pattern=".bamR1.fastq", full.names = TRUE))
fnRs <- sort(list.files(path, pattern=".bamR2.fastq", full.names = TRUE))

# Extract sample names, assuming filenames have format: xxx_SAMPLENAME_XXX.fastq
sample.names <- sapply(strsplit(basename(fnFs), "_"), `[`, 2)
```

##Trim primers and quality filter reads
```{r}
FWD <- "CTCTTTCCCTACACGACGCTCTTCCGATCTCCTACGGGNGGCWGCAG"  ## forward primer sequence
REV <- "CTGGAGTTCAGACGTGTGCTCTTCCGATCTGACTACHVGGGTATCTAATCC"  ## reverse primer sequence

#Function to create all orientations of the input sequence
allOrients <- function(primer) {
   require(Biostrings)
   dna <- DNAString(primer)  # The Biostrings works w/ DNAString objects rather than character vectors
   orients <- c(Forward = dna, Complement = complement(dna), Reverse = reverse(dna),
      RevComp = reverseComplement(dna))
   return(sapply(orients, toString))  # Convert back to character vector
}

#Apply function on primer sequence
FWD.orients <- allOrients(FWD)
REV.orients <- allOrients(REV)
FWD.orients
REV.orients

#The presence of ambiguous bases (Ns) in the sequencing reads makes accurate mapping of short primer sequences difficult. Next we are going to pre-filter the sequences just to remove those with Ns, but perform no other filtering.

fnFs.filtN <- file.path(path, "filtN", basename(fnFs)) # Put N-filterd files in filtN/ subdirectory
fnRs.filtN <- file.path(path, "filtN", basename(fnRs))
filterAndTrim(fnFs, fnFs.filtN, fnRs, fnRs.filtN, maxN = 0, multithread = TRUE)

#Function to count number of reads in which the primer is found
primerHits <- function(primer, fn) {
````
````

````
    nhits <- vcountPattern(primer, sread(readFastq(fn)), fixed = FALSE)
    return(sum(nhits > 0))
}

#Apply function to check for primers
rbind(FWD.ForwardReads = sapply(FWD.orients, primerHits, fn = fnFs.filtN[[1]]),
    FWD.ReverseReads = sapply(FWD.orients, primerHits, fn = fnRs.filtN[[1]]),
    REV.ForwardReads = sapply(REV.orients, primerHits, fn = fnFs.filtN[[1]]),
    REV.ReverseReads = sapply(REV.orients, primerHits, fn = fnRs.filtN[[1]]))

#Inspect read quality profiles

#Forward reads
plotQualityProfile(fnFs[1:2])
#Reverse reads
plotQualityProfile(fnRs[1:2])

#Reverse reads are usually of much worse quality than forward reads. Especially towards the end. Truncate the reads approximately where the quality score drops below 30-35.

# Place filtered files in filtered/ subdirectory
filtFs <- file.path(path, "filtered", paste0(sample.names, "_F_filt.fastq.gz"))
filtRs <- file.path(path, "filtered", paste0(sample.names, "_R_filt.fastq.gz"))

#Filter reads
out <- filterAndTrim(fnFs, filtFs, fnRs, filtRs, truncLen=c(290,200),
            maxN=0, maxEE=c(2,2), truncQ=2, rm.phix=TRUE,
            compress=TRUE, multithread=FALSE) #On Linux or Mac you can set multithread=TRUE

head(out)
```

##Dereplicate, learn error rates, and run DADA2
```{r}
#Forward reads
errF <- learnErrors(filtFs, multithread=FALSE)

#Reverse reads
errR <- learnErrors(filtRs, multithread=FALSE)

#visualize the estimated error rates
plotErrors(errF, nominalQ=TRUE)
plotErrors(errR, nominalQ=TRUE)

#Dereplicate identical reads
derepFs <- derepFastq(filtFs, verbose=TRUE)
derepRs <- derepFastq(filtRs, verbose=TRUE)

#Name the derep-class objects by the sample names
names(derepFs) <- sample.names
names(derepRs) <- sample.names
````

````
#Run dada2

#Forward reads
dadaFs <- dada(derepFs, err=errF, pool = TRUE , multithread=TRUE)

#Reverse reads
dadaRs <- dada(derepRs, err=errR, pool = TRUE , multithread=TRUE)

#Inspecting the returned dada-class object:

dadaFs[[1]]
dadaRs[[1]]
```
##Merge forward and reverse reads and construct sequence table
```{r}
mergers <- mergePairs(dadaFs, derepFs, dadaRs, derepRs, verbose=TRUE)
#Inspect the merger data.frame from the first sample
head(mergers[[1]])

#We can now construct an amplicon sequence variant table (ASV) table, a higher-resolution version of the OTU table produced by traditional methods.
seqtab <- makeSequenceTable(mergers)

#Check the number of sequence variants
dim(seqtab)

#Inspect distribution of sequence lengths
table(nchar(getSequences(seqtab)))

#Sequences that are much longer or shorter than expected may be the result of non-specific priming. You can remove non-target-length sequences with base R manipulations of the sequence table

seqtab2 <- seqtab[,nchar(colnames(seqtab)) %in% seq(440,466)] #Insert target length

#Check distribution again
table(nchar(getSequences(seqtab2)))

#Inspect mean sequence length
dna <- DNAStringSet(getSequences(seqtab.nochim))
writeXStringSet(dna,   file="C://Benni/WP1_Illumina/dna.fasta")
meansequencelength <- as.data.frame(table(nchar(getSequences(dna))))
meansequencelength$Var1 <- as.numeric(as.character(meansequencelength$Var1))
meansequencelength$total <- meansequencelength$Var1*meansequencelength$Freq
sum(meansequencelength$total)/sum(meansequencelength$Freq)
```
##Remove chimeras
```{r}
seqtab.nochim <- removeBimeraDenovo(seqtab2, method="consensus", multithread=TRUE, verbose=TRUE)

dim(seqtab.nochim)
````

sum(seqtab.nochim)/sum(seqtab2)
```
##Track reads through the pipeline
```{r}
#As a final check of our progress, we will look at the number of reads that made it through each step in the pipeline:

getN <- function(x) sum(getUniques(x))

dadaFs <- readRDS("C://Benni/WP1_Illumina/dadaFs.rds")
dadaRs <- readRDS("C://Benni/WP1_Illumina/dadaRS.rds")
out <- readRDS("C://Benni/WP1_Illumina/out.rds")
mergers <- readRDS("C://Benni/WP1_Illumina/mergers.rds")
seqtab.nochim <- readRDS("C://Benni/WP1_Illumina/seqtab.nochim.rds")

track <- cbind(out, sapply(dadaFs, getN), sapply(dadaRs, getN), sapply(mergers, getN), rowSums(seqtab.nochim))

# If processing a single sample, remove the sapply calls: e.g. replace sapply(dadaFs, getN) with getN(dadaFs)
colnames(track) <- c("input", "filtered", "denoisedF", "denoisedR", "merged", "nonchim")
rownames(track) <- sample.names
head(track)
```
##Assign taxonomy
```{r}
#Download the GTDB_bac-arc_ssu_r86.fa.gz file, and place it in the directory with the fastq files.
taxa_gtdb <- assignTaxonomy(seqtab.nochim, "C://Benni/GTDB_bac-arc_ssu_r86.fa.gz", multithread=TRUE)
#Lets inspect the taxonomic assignments:
taxa.print <- taxa_gtdb # Removing sequence rownames for display only
rownames(taxa.print) <- NULL
head(taxa.print)
```
#Make phylogenetic tree
```{r}
#Generate alignment of sequences for phylogenetic tree generation
seqs <- getSequences(seqtab.nochim)
names(seqs) <- seqs # This propagates to the tip labels of the tree
alignment <- AlignSeqs(DNAStringSet(seqs), anchor=NA)

long_names <- names(seq) ##keep the long names for later.
short_names <- substr(names(seq), 1, 5)%>% make.names(unique = TRUE)
names(seq ) <- short_names

saveRDS(alignment, "C://Benni/WP1_Illumina/alignment.rds")
writeXStringSet(alignment,   file="C://Benni/WP1_Illumina/alignment.fasta")

#Run FastTree in bash/Windows command line: FastTree -gtr -nt alignment_renamed.fasta > tree_file

#Import tree

````
library(ape)
tree <- read_tree("C://Benni/WP1_Illumina/tree_file")
```
#Export ESV Table and Sequences
```{r}
rownames(seqtab.nochim) <- sample.names
sample.names

seqs <- colnames(seqtab.nochim)
otab <- otu_table(seqtab.nochim, taxa_are_rows=FALSE)
colnames(otab) <- paste0("seq", seq(ncol(otab)))
otab = t(otab)

#Just sequences
write.table(seqs, "dada_seqs.txt",quote=FALSE)
#OTU-like table
write.table(otab, "dada_table.txt",quote=FALSE,sep="\t")
#ESV Table
write.csv(cbind(t(seqtab.nochim), taxa), "ESVtable.csv", quote=FALSE)

```
#Construct phyloseq object
```{r}
#Load metadata
sample_data <- as.data.frame(read.table('C://Benni/WP1_Illumina/metadata_Illumina.csv',
header=TRUE, sep=";"))

#Change rownames of metadata table to be the same as sample names in seqtab.nochim
sample_data$SampleID <- paste("1#", sample_data$SampleID, sep="")
rownames(sample_data) <- sample_data[,1]

#make levels for metadata factors
sample_data$Position <- factor(sample_data$Position, levels= c("Anal Swab", "Sticking",
"Singeing", "Polishing", "Evisceration", "Classification", "Truck","Lock brush (Clean area)", "Lock
liquid (Clean area)", "Polishing tunnel (Whips)", "Polishing tunnel (Nozzles)", "Polishing tunnel
(Water)", "Evisceration (Knife basin sterile)", "Evisceration (Gloves)", "Evisceration (Knifes)",
"Evisceration (Aprons)", "Splitting saw", "Classification (Railing)", "Classification (Gloves)",
"Cooling chamber (Wall)", "Truck (Gloves)", "Truck (Wall)", "Lock (Hands)", "Lock (Shoes)"))

#Load taxa and ESV table
seqtab.nochim <- readRDS("C://Benni/WP1_Illumina/seqtab.nochim.rds")
taxa_gtdb <- readRDS("C://Benni/WP1_Illumina/taxa_gtdb.rds")

#Combine into phyloseq object
physeq <- phyloseq(otu_table(seqtab.nochim, taxa_are_rows=FALSE),
            sample_data(sample_data),
            tax_table(taxa_gtdb),
            phy_tree(tree))
physeq

saveRDS(physeq,"C://Benni/WP1 Pacbio reads neu/physeq.rds")
```
````
````

````
##Community analysis
#Libraries
```{r Libraries}
#load libraries

library(phyloseq)
library(biomformat)
library(ggplot2)
library(ggthemr)
library(ggforce)
library(reshape)
library(magrittr)
library(data.table)
library(vegan)
library(plyr)
library(scales)
library(colourpicker)
library(dplyr)
library(decontam)
library(ggpubr)
library(readxl)
library(OTUtable)
library(ggsci)
library(RColorBrewer)
library(ampvis2)
library(superheat)
```

#Functions
=============

```{r Custom functions not part of R packages}
## Rarefaction curve, ggplot style
ggrare <- function(physeq, step = 10, label = NULL, color = NULL, plot = TRUE, parallel =
FALSE, se = TRUE) {
  ## Args:
  ## - physeq: phyloseq class object, from which abundance data are extracted
  ## - step: Step size for sample size in rarefaction curves
  ## - label: Default `NULL`. Character string. The name of the variable
  ##         to map to text labels on the plot. Similar to color option
  ##         but for plotting text.
  ## - color: (Optional). Default 'NULL'. Character string. The name of the
  ##         variable to map to colors in the plot. This can be a sample
  ##         variable (among the set returned by
  ##         'sample_variables(physeq)' ) or taxonomic rank (among the set
  ##         returned by 'rank_names(physeq)').
  ##
  ##         Finally, The color scheme is chosen automatically by
  ##         'link{ggplot}', but it can be modified afterward with an
  ##         additional layer using 'scale_color_manual'.
  ## - color: Default `NULL`. Character string. The name of the variable
````
````

```
##          to map to text labels on the plot. Similar to color option
##          but for plotting text.
## - plot:  Logical, should the graphic be plotted.
## - parallel: should rarefaction be parallelized (using parallel framework)
## - se:    Default TRUE. Logical. Should standard errors be computed.
## require vegan
x <- as(otu_table(physeq), "matrix")
if (taxa_are_rows(physeq)) { x <- t(x) }

## This script is adapted from vegan `rarecurve` function
tot <- rowSums(x)
S <- rowSums(x > 0)
nr <- nrow(x)

rarefun <- function(i) {
   cat(paste("rarefying sample", rownames(x)[i]), sep = "\n")
   n <- seq(1, tot[i], by = step)
   if (n[length(n)] != tot[i]) {
      n <- c(n, tot[i])
   }
   y <- rarefy(x[i, ,drop = FALSE], n, se = se)
   if (nrow(y) != 1) {
         rownames(y) <- c(".S", ".se")
      return(data.frame(t(y), Size = n, Sample = rownames(x)[i]))
   } else {
      return(data.frame(.S = y[1, ], Size = n, Sample = rownames(x)[i]))
   }
}
if (parallel) {
   out <- mclapply(seq_len(nr), rarefun, mc.preschedule = FALSE)
} else {
   out <- lapply(seq_len(nr), rarefun)
}
df <- do.call(rbind, out)

## Get sample data
if (!is.null(sample_data(physeq, FALSE))) {
   sdf <- as(sample_data(physeq), "data.frame")
   sdf$Sample <- rownames(sdf)
   data <- merge(df, sdf, by = "Sample")
   labels <- data.frame(x = tot, y = S, Sample = rownames(x))
   labels <- merge(labels, sdf, by = "Sample")
}

## Add, any custom-supplied plot-mapped variables
if( length(color) > 1 ){
   data$color <- color
   names(data)[names(data)=="color"] <- deparse(substitute(color))
   color <- deparse(substitute(color))
}
if( length(label) > 1 ){
   labels$label <- label
```

```
    names(labels)[names(labels)=="label"] <- deparse(substitute(label))
    label <- deparse(substitute(label))
  }

  p <- ggplot(data = data, aes_string(x = "Size", y = ".S", group = "Sample", color = color))
  p <- p + labs(x = "Sample Size", y = "Species Richness")
  if (!is.null(label)) {
    p <- p + geom_text(data = labels, aes_string(x = "x", y = "y", label = label, color = color),
                       size = 4, hjust = 0)
  }
  p <- p + geom_line()
  if (se) { ## add standard error if available
    p <- p + geom_ribbon(aes_string(ymin = ".S - .se", ymax = ".S + .se", color = NULL, fill =
color), alpha = 0.2)
  }
  if (plot) {
    plot(p)
  }
  invisible(p)
}

fast_melt = function(physeq){
  # supports "naked" otu_table as `physeq` input.
  otutab = as(otu_table(physeq), "matrix")
  if(!taxa_are_rows(physeq)){otutab <- t(otutab)}
  otudt = data.table(otutab, keep.rownames = TRUE)
  setnames(otudt, "rn", "taxaID")
  # Enforce character taxaID key
  otudt[, taxaIDchar := as.character(taxaID)]
  otudt[, taxaID := NULL]
  setnames(otudt, "taxaIDchar", "taxaID")
  # Melt count table
  mdt = melt.data.table(otudt,
                        id.vars = "taxaID",
                        variable.name = "SampleID",
                        value.name = "count")
  # Remove zeroes, NAs
  mdt <- mdt[count > 0][!is.na(count)]
  # Calculate relative abundance
  mdt[, RelativeAbundance := count / sum(count), by = SampleID]
  if(!is.null(tax_table(physeq, errorIfNULL = FALSE))){
    # If there is a tax_table, join with it. Otherwise, skip this join.
    taxdt = data.table(as(tax_table(physeq, errorIfNULL = TRUE), "matrix"), keep.rownames =
TRUE)
    setnames(taxdt, "rn", "taxaID")
    # Enforce character taxaID key
    taxdt[, taxaIDchar := as.character(taxaID)]
    taxdt[, taxaID := NULL]
    setnames(taxdt, "taxaIDchar", "taxaID")
    # Join with tax table
    setkey(taxdt, "taxaID")
    setkey(mdt, "taxaID")
```

```
    mdt <- taxdt[mdt]
  }
  return(mdt)
}

summarize_taxa = function(physeq, Rank, GroupBy = NULL){
  Rank <- Rank[1]
  if(!Rank %in% rank_names(physeq)){
    message("The argument to `Rank` was:\n", Rank,
            "\nBut it was not found among taxonomic ranks:\n",
            paste0(rank_names(physeq), collapse = ", "), "\n",
            "Please check the list shown above and try again.")
  }
  if(!is.null(GroupBy)){
    GroupBy <- GroupBy[1]
    if(!GroupBy %in% sample_variables(physeq)){
      message("The argument to `GroupBy` was:\n", GroupBy,
              "\nBut it was not found among sample variables:\n",
              paste0(sample_variables(physeq), collapse = ", "), "\n",
              "Please check the list shown above and try again.")
    }
  }
  # Start with fast melt
  mdt = fast_melt(physeq)
  if(!is.null(GroupBy)){
    # Add the variable indicated in `GroupBy`, if provided.
    sdt = data.table(SampleID = sample_names(physeq),
                     var1 = get_variable(physeq, GroupBy))
    setnames(sdt, "var1", GroupBy)
    # Join
    setkey(sdt, SampleID)
    setkey(mdt, SampleID)
    mdt <- sdt[mdt]
  }
   # Summarize
Nsamples = nsamples(physeq)
  summarydt = mdt[, list(meanRA = sum(RelativeAbundance)/Nsamples,
                         sdRA = sd(RelativeAbundance),
                         minRA = min(RelativeAbundance),
                         maxRA = max(RelativeAbundance)),
                  by = c(Rank, GroupBy)]
  return(summarydt)
}


#find top taxa per sample function
find.top.taxa <- function(x,taxa){
  require(phyloseq)
  top.taxa <- tax_glom(x, taxa)
  otu <- otu_table(t(top.taxa))
  if (taxa_are_rows(otu)){
    otu <- t(otu)
```

````
    }
  tax <- tax_table(top.taxa)
  j<-apply(otu,1,which.max)
  k <- j[!duplicated(j)]
  l <- data.frame(tax[k,])
  m <- data.frame(otu[,k])
  colnames(m) = l[,taxa]
  n <- colnames(m)[apply(m,1,which.max)]
  m[,taxa] <- n
  return(m)
}

#' Generate all pages with facet_wrap_paginate
#'
#' This is a wrapper for [facet_wrap_paginate()] to generate all of the pages,
#' rather than just one at a time.
#'
#' @inheritParams facet_wrap_paginate
#' @inheritDotParams facet_wrap_paginate
#' @param plot A ggplot object.
#' @return A list, where each element is one page of plots.
#' @family ggforce facets
#' @examples
#' g <- ggplot(diamonds, aes(x = cut, y = price)) + geom_boxplot()
#' gl <- gen_all_pages_fwp(g, "color", nrow = 2, ncol = 3)
#' gl[[1]]
#' @export

gen_all_pages_fwp <- function(plot, facets, ...){
    dots <- list(...)
    if (is.null(dots$nrow) || is.null(dots$ncol)){
        msg <- paste("nrow and/or ncol is NULL. Please supply values for both",
                     "arguments; otherwise, call facet_wrap directly.")
        stop(msg)
    }
    n <- n_pages(plot + facet_wrap_paginate(facets, ..., page = 1))
    plot_list <- lapply(1:n, function(i) {
        plot + facet_wrap_paginate(facets, ..., page = i)
    })
    return(plot_list)
}
```

#Initial data exploration
```{r}
physeq <- readRDS("C://Benni/WP1 Pacbio reads neu/physeq.rds")
#Check if phyloseq object was created correctly
physeq
nsamples(physeq)
ntaxa(physeq)
sample_variables(physeq)
rank_names(physeq)
````

```
#Summarize sequencing depths, in general...
sdt = data.table(as(sample_data(physeq), "data.frame"),
                 TotalReads = sample_sums(physeq), keep.rownames = TRUE)

pSeqDepth = ggplot(sdt, aes(TotalReads)) + geom_histogram() + ggtitle("Sequencing Depth")
pSeqDepth

# ...and by category
pSeqDepth + facet_wrap(~Type)

#Filter Taxa
#Taxa total counts histogram
tdt = data.table(tax_table(physeq),
                 TotalCounts = taxa_sums(physeq),
                 OTU = taxa_names(physeq))
ggplot(tdt, aes(TotalCounts)) + geom_histogram() + ggtitle("Histogram of Total Counts")

#Count number of singletons, doubletons, etc.
tdt[(TotalCounts <= 0), .N]
tdt[(TotalCounts <= 1), .N]
tdt[(TotalCounts <= 2), .N]
tdt[(TotalCounts <= 5), .N]

#Calculate the cumulative sum of OTUs that would be filtered at every possible value of such a
threshold, from zero to the most-observed OTU.
taxcumsum = tdt[, .N, by = TotalCounts]
setkey(taxcumsum, TotalCounts)
taxcumsum[, CumSum := cumsum(N)]

#plot
pCumSum = ggplot(taxcumsum, aes(TotalCounts, CumSum)) +
  geom_point() +
  xlab("Filtering Threshold, Minimum Total Counts") +
  ylab("OTUs Filtered") +
  ggtitle("OTUs that would be filtered vs. the minimum count threshold")
pCumSum

# Zoom-in on the region between zero and 100 total counts
pCumSum + xlim(0, 100)

#Taxa prevalence histogram
mdt = fast_melt(physeq)

#We define Prevalence here as the number of times an OTU is observed at least once.
prevdt = mdt[, list(Prevalence = sum(count > 0),
                    TotalCounts = sum(count)),
             by = taxaID]

prevdt[(Prevalence <= 0), .N]
prevdt[(Prevalence <= 1), .N]
prevdt[(Prevalence <= 2), .N]
```

````
#plot
ggplot(prevdt, aes(Prevalence)) + geom_histogram() + ggtitle("Histogram of Taxa Prevalence")

#Taxa cumulative sum
prevcumsum = prevdt[, .N, by = Prevalence]
setkey(prevcumsum, Prevalence)
prevcumsum[, CumSum := cumsum(N)]
pPrevCumSum = ggplot(prevcumsum, aes(Prevalence, CumSum)) +
  geom_point() +
  xlab("Filtering Threshold, Prevalence") +
  ylab("OTUs Filtered") +
  ggtitle("OTUs that would be filtered vs. the minimum count threshold")
pPrevCumSum

#Prevalence vs. Total Count Scatter plot
ggplot(prevdt, aes(Prevalence, TotalCounts)) + geom_point(size = 4, alpha = 0.75) +
scale_y_log10()

#Select and document Filtering Criteria
keepTaxa = prevdt[(Prevalence >= 1 & TotalCounts > 5), taxaID]

# Make new phyloseq object with filtered OTUs
physeq_filtered = prune_taxa(keepTaxa, physeq)
physeq_filtered
```

#Detect Contaminants
```{r}
library(decontam)

#Run decontam algorithm to identify possible contaminant OTUs
sample_data(physeq_filtered)$is.neg <- sample_data(physeq_filtered)$SampleorControl ==
"Control"
contam.prev05 <- isContaminant(physeq_filtered, method="prevalence", neg="is.neg",
threshold=0.5)

#Table with prevalence of contaminant OTUs
table(contam.prev05)

#Number of contaminant OTUs
sum(contam.prev05$contaminant)

#Make a list of contaminants
List_of_contaminants <- subset(contam.prev05,contam.prev05$contaminant == TRUE)
List_of_contaminants$OTUID <- rownames(List_of_contaminants)
taxa <- as.data.frame(taxa)
taxa$OTUID <- rownames(taxa)
List_of_contaminants <- merge(List_of_contaminants, taxa, by="OTUID")

write.table(List_of_contaminants, file = "C://Benni/WP1 Pacbio reads
neu/Analysis/List_of_contaminants_slaughterhouse.txt", sep = ";", dec = ".", quote = FALSE,
````

```
row.names = FALSE)

#Make phyloseq object of presence-absence in negative controls
physeq.neg <- prune_samples(sample_data(physeq_filtered)$SampleorControl == "Control",
physeq_filtered)
physeq.neg.presence <- transform_sample_counts(physeq.neg, function(abund) 1*(abund>0))

#Make phyloseq object of presence-absence in true positive samples
physeq.pos <- prune_samples(sample_data(physeq_filtered)$SampleorControl == "Sample",
physeq_filtered)
physeq.pos.presence <- transform_sample_counts(physeq.pos, function(abund) 1*(abund>0))

#Make data.frame of prevalence in positive and negative samples
df.pres <- data.frame(prevalence.pos=taxa_sums(physeq.pos.presence),
prevalence.neg=taxa_sums(physeq.neg.presence),
                    contam.prev=contam.prev05)
ggplot(data=df.pres, aes(x=prevalence.neg, y=prevalence.pos, color=contam.prev.contaminant)) +
geom_jitter()

#OTUs seem to split into a branch that shows up mostly in positive samples, and another that shows
up mostly in negative controls. The contaminant assignment has done a good job of identifying
those mostly in negative controls. However, five ASVs are present in more than 2 neg. controls, but
were not identified as contaminants by decontam. Those might be contaminants and should be
removed manually.
over2 <- rownames(subset(df.pres, prevalence.neg > 2))

#remove contaminant ASVs
#List all OTUs
all_taxa = taxa_names(physeq_filtered)
#make a list of all contaminant OTUs
my_contaminants <- c(subset(List_of_contaminants, select = OTUID))
#add manually picked contaminants to list
my_contaminants <- unique(unlist(c(my_contaminants, over2)))
#exclude contaminants from all OTUs
my_taxa <- all_taxa[!(all_taxa %in% my_contaminants)]
#create new phyloseq object without contaminants
physeq_no_cont = prune_taxa(my_taxa, physeq_filtered)
physeq_no_cont

#The following ensures that features with ambiguous phylum annotation are also removed. Note the
flexibility in defining strings that should be considered ambiguous annotation.
physeq_no_cont <- subset_taxa(physeq_no_cont, !is.na(Phylum) & !Phylum %in% c("",
"uncharacterized","Vertebrata","Euglenozoa"))

#Filter out samples less than 200 reads
physeq_no_cont
Sequencedepth <- as.matrix(sample_sums(physeq_no_cont))
physeq_no_cont = prune_samples(sample_sums(physeq_no_cont)>=200, physeq_no_cont)
Sequencedepth <- as.matrix(sample_sums(physeq_no_cont))
physeq_no_cont

#Rename ASVs to something more convenient for downstream analysis, while automatically
```

retaining the corresponding unique sequence identifier
sequences <- Biostrings::DNAStringSet(taxa_names(physeq_no_cont))
names(sequences) <- taxa_names(physeq_no_cont)
physeq_no_cont <- merge_phyloseq(physeq_no_cont, sequence)
physeq_no_cont
taxa_names(physeq_no_cont) <- paste0("ASV", seq(ntaxa(physeq_no_cont)))

physeq_no_cont <- subset_samples(physeq_no_cont, Facility =="Slaughterhouse")
physeq_no_cont <- prune_taxa(taxa_sums(physeq_no_cont) > 1, physeq_no_cont)
```

#Community Analysis
```{r}
#Create table, number of features for each phyla
table(tax_table(physeq_no_cont)[, "Phylum"], exclude = NULL)

#calculate rarefaction curve for all samples
rarefaction_curve <- ggrare(physeq_no_cont, step = 100, color = "Position", label =
"SampleNumber", se = FALSE)

#Calculate percentage of ASVs classified at the Species level
taxa_no_cont <- as.data.frame(tax_table(physeq_no_cont))
100*(1-(sum(is.na(taxa_no_cont$Species))/length(taxa_no_cont$Species)))

#Subset phyloseq object to only meat samples
physeq_slaughterhouse_meat <- subset_samples(physeq_no_cont, Type =="Meat")

#Calculate Alpha Diversity for meat samples
pAlpha = plot_richness(physeq_slaughterhouse_meat,
                    color = "Position",
                    measures = c("Observed", "Chao1", "Shannon", "InvSimpson"),
                    title = "Alpha Diversity, Slaughterhouse", x="Position")
pAlpha + geom_point(size = 5)

#Store plot as a new data variable and calculate mean +- standard deviation
alphadt = data.table(pAlpha$data)
alphadt <- dplyr::filter(alphadt, SampleorControl=="Sample")
alphadtmean <- alphadt %>%
              group_by(Position, variable) %>%
              summarise(avg = mean(value), sd = sd(value)) %>%
              arrange(variable)

write.table(alphadtmean, file = "C://Benni/WP1 Pacbio reads neu/Analysis/Alpha_div_pacbio.csv",
sep = ";", dec = ",")

#Define the plot
cols <- c("red","blue","darkgreen","orange")

alpha_div_meat <- ggplot(data = alphadt,mapping =
aes(x=Position,y=value,group=variable,colour=variable,fill=variable))+geom_point()
+geom_smooth(span=0.5)+ggtitle("Microbial diversity on meat along the processing line")
+theme_bw()+scale_fill_manual(values = cols,aesthetics = c("colour", "fill"))

```
+facet_wrap(~variable,scales = "free")+theme(axis.text=element_text(size=15, angle =
90),legend.position = "none")

ggsave("C://Benni/WP1 Pacbio reads neu/Analysis/Alphadiv_meat_plot.pdf",device="pdf", width =
15,height = 10,dpi = 600)
dev.off()

#Test for normal distribution of individual alpha-div indices
alphadt_observed <- dplyr::filter(alphadt, variable=="Observed")
shapiro.test(alphadt_observed$value)
qqnorm(alphadt_observed$value);qqline(alphadt_observed$value, col = 2)
plot(density(alphadt_observed$value,na.rm=TRUE))

alphadt_Chao1 <- dplyr::filter(alphadt, variable=="Chao1")
shapiro.test(alphadt_Chao1$value)
qqnorm(alphadt_Chao1$value);qqline(alphadt_Chao1$value, col = 2)
plot(density(alphadt_Chao1$value,na.rm=TRUE))

alphadt_Shannon <- dplyr::filter(alphadt, variable=="Shannon")
shapiro.test(alphadt_Shannon$value)
qqnorm(alphadt_Shannon$value);qqline(alphadt_Shannon$value, col = 2)
plot(density(alphadt_Shannon$value,na.rm=TRUE))

alphadt_InvSimpson <- dplyr::filter(alphadt, variable=="InvSimpson")
shapiro.test(alphadt_InvSimpson$value)
qqnorm(alphadt_InvSimpson$value);qqline(alphadt_InvSimpson$value, col = 2)
plot(density(alphadt_InvSimpson$value,na.rm=TRUE))

#T-test for normal distributed groups
alphadiv_t.test <- compare_means(value ~ Position, group.by = "variable", data = alphadt, method
= "t.test",p.adjust.method = "BH")

#Wilcox-test for not normal distributed groups
alphadiv_wilcoxres <- compare_means(value ~ Position, group.by = "variable", data = alphadt,
method = "wilcox",p.adjust.method = "BH")

#Beta Diversity

#Define new object with relative abundance
mpra = transform_sample_counts(physeq_no_cont, function(x) x / sum(x))
#Filter this new object to only meat samples
mpraf = subset_samples(mpra, SampleorControl=="Sample")
mpraf = subset_samples(mpraf, Type=="Meat")

#t-SNE plot
library(tsnemicrobiota)
t_SNE <- tsne_phyloseq(mpraf, distance = "bray", perplexity = 25,
  precomputed_distance = NULL, pseudocounts = 1, verbose = 1,
  rng_seed = NULL, philr_options = list(), control = list())

tsne_plot <- plot_tsne_phyloseq(mpraf, t_SNE,
    color = 'Position', title='t-SNE (bray)') +
```

```
  geom_point(size=5)
ggsave("C://Benni/WP1 Pacbio reads neu/Analysis/tSNE.pdf",device="pdf", width = 15,height = 10,dpi = 600)

#Find the number of samples in which a taxa appears at least once.
prevdf = apply(X = otu_table(physeq_no_cont),
               MARGIN = ifelse(taxa_are_rows(physeq_no_cont), yes = 1, no = 2),
               FUN = function(x){sum(x > 0)})
#Add taxonomy and total read counts to this data.frame
prevdf = data.frame(Prevalence = prevdf,
                    TotalAbundance = taxa_sums(physeq_no_cont),
                    tax_table(physeq_no_cont))

#Are there phyla that are comprised of mostly low-prevalence features? Compute the total and average prevalences of the features in each phylum.
Phyla_prevalence <- plyr::ddply(prevdf, "Phylum", function(df1)
{cbind(mean(df1$Prevalence),sum(df1$Prevalence))})

#Subset to the remaining phyla
prevdf1 = subset(prevdf, Phylum %in% get_taxa_unique(physeq_no_cont, "Phylum"))
Phyla_prevalence_plot <- ggplot(prevdf1, aes(TotalAbundance, Prevalence / nsamples(physeq_no_cont),color=Phylum)) +
  #Include a guess for parameter
  geom_point(size = 2, alpha = 0.7) + ggtitle("Prevalence of Phyla at the Slaughterhouse/ Pacbio")+
  scale_x_log10() +  xlab("Total Abundance") + ylab("Prevalence [Frac. Samples]") +
  facet_wrap(~Phylum) + theme(legend.position="none")
ggsave("C://Benni/WP1 Pacbio reads neu/Analysis/Phyla_prevalence.eps",device="eps", width = 15,height = 10,dpi = 600)

#Agglomerate the ASVs corresponding to closely related taxa.
length(get_taxa_unique(physeq_no_cont, taxonomic.rank = "Genus"))

#Agglomerate to genera and phyla
ps_genera = tax_glom(physeq_no_cont, "Genus", NArm = TRUE)
ps_phyla = tax_glom(physeq_no_cont, "Phylum", NArm = TRUE)
ps_phyla_rel <- transform_sample_counts(ps_phyla, function(x){x / sum(x)}*100)

phyla_rel = psmelt(ps_phyla_rel)
phyla_rel_agg = aggregate(Abundance ~ Position + Type + Phylum, data=phyla_rel, mean)

colors_phyla <- length(unique(phyla_rel$Phylum))
getPalette <- colorRampPalette(brewer.pal(8, "Set1"))

plot_phyla <- ggplot(phyla_rel_agg, aes(x=Position, y=Abundance, fill = Phylum))+
geom_bar(stat='identity',position="fill")+ guides(fill = guide_legend(ncol = 1))+labs(y="Relative Abundance (%)", x="Position")+theme_bw() + theme(legend.text = element_text(size=15,face = "italic"),axis.title=element_text(size=20),axis.text=element_text(size=15,angle = 90),strip.text.x = element_text(size=20),strip.text.y = element_text(size=20),panel.border = element_blank(), panel.grid.major = element_blank(), panel.grid.minor = element_blank(), axis.line = element_line(colour = "black"))+facet_grid(~Type,scales = "free")+scale_fill_manual(values = getPalette(colors_phyla))+ggtitle("Relative abundances of Phyla")
ggsave("C://Benni/WP1 Pacbio reads neu/Analysis/Phyla_slaughterhouse.eps",device="eps", width
```

```
= 15,height = 10,dpi = 600)

Phyla_slaughterhouse_data <- data.table(plot_phyla$data)
write.table(Phyla_slaughterhouse_data, file = "C://Benni/WP1 Pacbio reads
neu/Analysis/Phyla_slaughterhouse_data.csv", sep = ";", dec = ",")

#Transform to relative abundances
ps_genera_rel = transform_sample_counts(ps_genera, function(x){x / sum(x)}*100)

#custom function for exploratory plots
plot_abundance = function(physeq,title = "",
                          Facet = "Genus", Color = "Phylum"){
  # Arbitrary subset, based on Phylum, for plotting
  p1f = subset_taxa(physeq) #(physeq, Phylum %in% c("Actinobacteria")) To explore individual
Phyla.
  mphyseq = psmelt(p1f)
  mphyseq <- subset(mphyseq, Abundance > 0)
  ggplot(data = mphyseq, mapping = aes_string(x = "Position",y = "Abundance",
                                 color = Color, fill = Color)) +
    geom_violin(fill = NA) +
    geom_point(size = 3, alpha = 0.3,
               position = position_jitter(width = 0.3)) +
    facet_wrap(facets = Facet) + scale_y_log10()+
    theme(legend.position="bottom")
}

#subset to meat and surface samples only
ps_meat = subset_samples(ps_genera_rel,Type=="Meat")
ps_surface = subset_samples(ps_genera_rel, Type=="Surface")

#plot distribution of all genera on meat samples
all_genera_on_meat <- plot_abundance(ps_meat, Facet = "Genus", Color = "Phylum")
+theme(axis.text.x =element_text(size=15, angle = 90))
all_genera_on_surface <- plot_abundance(ps_surface, Facet = "Genus", Color = "Phylum")
+theme(axis.text.x =element_text(size=15, angle = 90))

#plot facets on individual pages
all_genera_on_meat_page1 <- all_genera_on_meat + facet_wrap_paginate(~Genus, ncol = 5, nrow
= 4, page = 1)
all_genera_on_meat_page2 <- all_genera_on_meat + facet_wrap_paginate(~Genus, ncol = 5, nrow
= 4, page = 2)
all_genera_on_meat_page3 <- all_genera_on_meat + facet_wrap_paginate(~Genus, ncol = 5, nrow
= 4, page = 3)
all_genera_on_meat_page4 <- all_genera_on_meat + facet_wrap_paginate(~Genus, ncol = 5, nrow
= 4, page = 4)
all_genera_on_meat_page5 <- all_genera_on_meat + facet_wrap_paginate(~Genus, ncol = 5, nrow
= 4, page = 5)
all_genera_on_meat_page6 <- all_genera_on_meat + facet_wrap_paginate(~Genus, ncol = 5, nrow
= 4, page = 6)
all_genera_on_meat_page7 <- all_genera_on_meat + facet_wrap_paginate(~Genus, ncol = 5, nrow
= 4, page = 7)
all_genera_on_meat_page8 <- all_genera_on_meat + facet_wrap_paginate(~Genus, ncol = 5, nrow
```

```
= 4, page = 8)
all_genera_on_meat_page9 <- all_genera_on_meat + facet_wrap_paginate(~Genus, ncol = 5, nrow
= 4, page = 9)
all_genera_on_meat_page10 <- all_genera_on_meat + facet_wrap_paginate(~Genus, ncol = 5,
nrow = 4, page = 10)
all_genera_on_meat_page11 <- all_genera_on_meat + facet_wrap_paginate(~Genus, ncol = 5, nrow
= 4, page = 11)
all_genera_on_meat_page12 <- all_genera_on_meat + facet_wrap_paginate(~Genus, ncol = 5,
nrow = 4, page = 12)

all_genera_on_surface_page1 <- all_genera_on_surface + facet_wrap_paginate(~Genus, ncol = 4,
nrow = 4, page = 1)
all_genera_on_surface_page2 <- all_genera_on_surface + facet_wrap_paginate(~Genus, ncol = 4,
nrow = 4, page = 2)
all_genera_on_surface_page3 <- all_genera_on_surface + facet_wrap_paginate(~Genus, ncol = 4,
nrow = 4, page = 3)
all_genera_on_surface_page4 <- all_genera_on_surface + facet_wrap_paginate(~Genus, ncol = 4,
nrow = 4, page = 4)
all_genera_on_surface_page5 <- all_genera_on_surface + facet_wrap_paginate(~Genus, ncol = 4,
nrow = 4, page = 5)
all_genera_on_surface_page6 <- all_genera_on_surface + facet_wrap_paginate(~Genus, ncol = 4,
nrow = 4, page = 6)
all_genera_on_surface_page7 <- all_genera_on_surface + facet_wrap_paginate(~Genus, ncol = 4,
nrow = 4, page = 7)
all_genera_on_surface_page8 <- all_genera_on_surface + facet_wrap_paginate(~Genus, ncol = 4,
nrow = 4, page = 8)
all_genera_on_surface_page9 <- all_genera_on_surface + facet_wrap_paginate(~Genus, ncol = 4,
nrow = 4, page = 9)
all_genera_on_surface_page10 <- all_genera_on_surface + facet_wrap_paginate(~Genus, ncol = 4,
nrow = 4, page = 10)
all_genera_on_surface_page11 <- all_genera_on_surface + facet_wrap_paginate(~Genus, ncol = 4,
nrow = 4, page = 11)
all_genera_on_surface_page12 <- all_genera_on_surface + facet_wrap_paginate(~Genus, ncol = 4,
nrow = 4, page = 12)
all_genera_on_surface_page13 <- all_genera_on_surface + facet_wrap_paginate(~Genus, ncol = 4,
nrow = 4, page = 13)

ggsave(all_genera_on_meat_page1, file="C://Benni/WP1 Pacbio reads
neu/Analysis/All_genera_on_meat_page1.pdf", device = "pdf",width = 15,height = 10,dpi = 600)
ggsave(all_genera_on_meat_page2, file="C://Benni/WP1 Pacbio reads
neu/Analysis/All_genera_on_meat_page2.pdf", device = "pdf",width = 15,height = 10,dpi = 600)
ggsave(all_genera_on_meat_page3, file="C://Benni/WP1 Pacbio reads
neu/Analysis/All_genera_on_meat_page3.pdf", device = "pdf",width = 15,height = 10,dpi = 600)
ggsave(all_genera_on_meat_page4, file="C://Benni/WP1 Pacbio reads
neu/Analysis/All_genera_on_meat_page4.pdf", device = "pdf",width = 15,height = 10,dpi = 600)
ggsave(all_genera_on_meat_page5, file="C://Benni/WP1 Pacbio reads
neu/Analysis/All_genera_on_meat_page5.pdf", device = "pdf",width = 15,height = 10,dpi = 600)
ggsave(all_genera_on_meat_page6, file="C://Benni/WP1 Pacbio reads
neu/Analysis/All_genera_on_meat_page6.pdf", device = "pdf",width = 15,height = 10,dpi = 600)
ggsave(all_genera_on_meat_page7, file="C://Benni/WP1 Pacbio reads
neu/Analysis/All_genera_on_meat_page7.pdf", device = "pdf",width = 15,height = 10,dpi = 600)
ggsave(all_genera_on_meat_page8, file="C://Benni/WP1 Pacbio reads
```

```
neu/Analysis/All_genera_on_meat_page8.pdf", device = "pdf",width = 15,height = 10,dpi = 600)
ggsave(all_genera_on_meat_page9, file="C://Benni/WP1 Pacbio reads
neu/Analysis/All_genera_on_meat_page9.pdf", device = "pdf",width = 15,height = 10,dpi = 600)
ggsave(all_genera_on_meat_page10, file="C://Benni/WP1 Pacbio reads
neu/Analysis/All_genera_on_meat_page10.pdf", device = "pdf",width = 15,height = 10,dpi = 600)
ggsave(all_genera_on_meat_page11, file="C://Benni/WP1 Pacbio reads
neu/Analysis/All_genera_on_meat_page11.pdf", device = "pdf",width = 15,height = 10,dpi = 600)
ggsave(all_genera_on_meat_page12, file="C://Benni/WP1 Pacbio reads
neu/Analysis/All_genera_on_meat_page12.pdf", device = "pdf",width = 15,height = 10,dpi = 600)

ggsave(all_genera_on_surface_page1, file="C://Benni/WP1 Pacbio reads
neu/Analysis/All_genera_on_surface_page1.pdf", device = "pdf",width = 15,height = 10,dpi = 600)
ggsave(all_genera_on_surface_page2, file="C://Benni/WP1 Pacbio reads
neu/Analysis/All_genera_on_surface_page2.pdf", device = "pdf",width = 15,height = 10,dpi = 600)
ggsave(all_genera_on_surface_page3, file="C://Benni/WP1 Pacbio reads
neu/Analysis/All_genera_on_surface_page3.pdf", device = "pdf",width = 15,height = 10,dpi = 600)
ggsave(all_genera_on_surface_page4, file="C://Benni/WP1 Pacbio reads
neu/Analysis/All_genera_on_surface_page4.pdf", device = "pdf",width = 15,height = 10,dpi = 600)
ggsave(all_genera_on_surface_page5, file="C://Benni/WP1 Pacbio reads
neu/Analysis/All_genera_on_surface_page5.pdf", device = "pdf",width = 15,height = 10,dpi = 600)
ggsave(all_genera_on_surface_page6, file="C://Benni/WP1 Pacbio reads
neu/Analysis/All_genera_on_surface_page6.pdf", device = "pdf",width = 15,height = 10,dpi = 600)
ggsave(all_genera_on_surface_page7, file="C://Benni/WP1 Pacbio reads
neu/Analysis/All_genera_on_surface_page7.pdf", device = "pdf",width = 15,height = 10,dpi = 600)
ggsave(all_genera_on_surface_page8, file="C://Benni/WP1 Pacbio reads
neu/Analysis/All_genera_on_surface_page8.pdf", device = "pdf",width = 15,height = 10,dpi = 600)
ggsave(all_genera_on_surface_page9, file="C://Benni/WP1 Pacbio reads
neu/Analysis/All_genera_on_surface_page9.pdf", device = "pdf",width = 15,height = 10,dpi = 600)
ggsave(all_genera_on_surface_page10, file="C://Benni/WP1 Pacbio reads
neu/Analysis/All_genera_on_surface_page10.pdf", device = "pdf",width = 15,height = 10,dpi =
600)
ggsave(all_genera_on_surface_page11, file="C://Benni/WP1 Pacbio reads
neu/Analysis/All_genera_on_surface_page11.pdf", device = "pdf",width = 15,height = 10,dpi =
600)
ggsave(all_genera_on_surface_page12, file="C://Benni/WP1 Pacbio reads
neu/Analysis/All_genera_on_surface_page12.pdf", device = "pdf",width = 15,height = 10,dpi =
600)
ggsave(all_genera_on_surface_page13, file="C://Benni/WP1 Pacbio reads
neu/Analysis/All_genera_on_surface_page13.pdf", device = "pdf",width = 15,height = 10,dpi =
600)

#Bar plot of top 50 ASVs
top50otus = names(sort(taxa_sums(physeq_no_cont), TRUE)[1:50])
taxtab50 <- cbind(tax_table(physeq_no_cont), genus50 = NA)
taxtab50[top50otus, "genus50"] <- as(tax_table(physeq_no_cont)[top50otus, "Genus"],
   "character")
tax_table(physeq_no_cont) <- tax_table(taxtab50)
plot_bar(physeq_no_cont, "Position", fill = "genus50") + coord_flip()

physeq_no_cont_m = merge_samples(physeq_no_cont, "Position")

sample_data(physeq_no_cont_m)$Position <- levels(sample_data(physeq_no_cont)$Position)
```

```
physeq_no_cont_m = transform_sample_counts(physeq_no_cont_m, function(x) 100 * x/sum(x))
physeq_no_cont_mr <- rarefy_even_depth(physeq_no_cont_m,sample.size =
min(sample_sums(physeq_no_cont_m)))

sample_data(physeq_no_cont_m)$Position <- factor(sample_data(physeq_no_cont_m)$Position,
levels= c("Anal Swab", "Sticking", "Singeing", "Polishing", "Evisceration", "Classification",
"Truck", "Lock brush (Clean area)", "Lock liquid (Clean area)", "Evisceration (Knife basin sterile)",
"Polishing tunnel (Whips)", "Polishing tunnel (Nozzles)", "Polishing tunnel (Water)", "Evisceration
(Gloves)", "Evisceration (Knifes)", "Evisceration (Aprons)", "Splitting saw", "Classification
(Railing)", "Classification (Gloves)", "Cooling chamber (Wall)", "Cooling chamber (Wall_A)",
"Truck (Gloves)", "Truck (Wall)", "Lock (Hands_A)", "Lock (Shoes_A)", "Lock (Hands_C)",
"Lock (Shoes_C)"))

sample_data(physeq_no_cont_m)$Type <- factor(sample_data(physeq_no_cont_m)$Type, levels=
c("Meat","Surface"))

physeq_no_cont_m50 = prune_taxa(top50otus, physeq_no_cont_m)



top50ASVs_plot <- plot_bar(physeq_no_cont_m50, "Position", fill = "genus50")+
   ylab("Percentage of Sequences")+facet_grid(~Type,scales = "free")+scale_fill_igv()+theme_bw()
+ theme(legend.text = element_text(size=15,face =
"italic"),axis.title=element_text(size=20),axis.text=element_text(size=15,angle = 90),strip.text.x =
element_text(size=20),strip.text.y = element_text(size=20),panel.border = element_blank(),
panel.grid.major = element_blank(), panel.grid.minor = element_blank(), axis.line =
element_line(colour = "black"))+guides(fill=guide_legend(ncol=1))

ggsave(top50ASVs_plot, file="C://Benni/WP1 Pacbio reads
neu/Analysis/top50ASVs_plot_igv.pdf", device = "pdf",width = 15,height = 10,dpi = 600)

#Tree plot of all ASVs associated to Chryseobacterium
physeq_no_cont_r = transform_sample_counts(physeq_no_cont, function(x) 100 * x/sum(x))

physeq_chryseobacterium <- subset_taxa(physeq_no_cont_r, Genus=="Chryseobacterium")

physeq_chryseobacterium_surface <- subset_samples(physeq_chryseobacterium, Type
=="Surface")
physeq_chryseobacterium_surface <-  prune_taxa(taxa_sums(physeq_chryseobacterium_surface) >
1, physeq_chryseobacterium_surface)

chryseobacterium_asvs_taxonomy <- as.data.frame(tax_table(physeq_chryseobacterium_surface))
write.table(chryseobacterium_asvs_taxonomy, file = "C://Benni/WP1 Pacbio reads
neu/Analysis/chryseobacterium_asvs_taxonomy.csv", sep = ";", dec = ",")

chryseobacterium_asvs <- plot_bar(physeq_chryseobacterium, "Position", fill = "ASVID")
+ylab("Percentage of Sequences")+facet_grid(~Type,scales = "free")+scale_fill_manual(values =
getPalette(colors25))+theme_bw() + theme(legend.text = element_text(size=15,face =
"italic"),axis.title=element_text(size=20),axis.text=element_text(size=15,angle = 90),strip.text.x =
element_text(size=20),strip.text.y = element_text(size=20),panel.border = element_blank(),
panel.grid.major = element_blank(), panel.grid.minor = element_blank(), axis.line =
element_line(colour = "black"))+guides(fill=guide_legend(ncol=1))
```

ggsave(chryseobacterium_asvs, file="C://Benni/WP1 Pacbio reads neu/Analysis/chryseobacterium_ASVs_plot.pdf", device = "pdf",width = 15,height = 10,dpi = 600)

plot_tree(physeq_chryseobacterium_surface, nodelabf=nodeplotboot(), ladderize="left", color="Position", size="abundance",label.tips = "Species", plot.margin=0.45)+scale_colour_manual(values = getPalette(colors25))+guides(col = guide_legend(ncol=1),override.aes = list(size=10),show.guide=F) +theme(legend.text=element_text(size=10))
ggsave("C://Benni/WP1 Pacbio reads neu/Analysis/chryseobacterium_tree.pdf",device="pdf", width = 15,height = 10,dpi = 600)
```
#Top_genera_origin
```{r}
Top_genera_origin <- read.table(file = "Top_genera_origin.csv", sep = ";", header = TRUE)
Top_genera_origin$Origin <- factor(Top_genera_origin$Origin, levels = c("Animal", "Sludge/Manure", "Skin", "Soil", "Water", "Plant", "Air"))

plot_top_genera <- ggplot(Top_genera_origin, aes(x=Provenance, y=Number.of.Genera, fill=Origin))+geom_bar(stat = "identity", position="fill")+ ylab("Proportion of genera") +theme_bw() +theme(legend.text = element_text(size=15),axis.title=element_text(size=20),axis.text.y =element_text(size=15),strip.text.x = element_text(size=20),strip.text.y = element_text(size=20),panel.border = element_blank(), panel.grid.major = element_blank(), panel.grid.minor = element_blank(), axis.line = element_line(colour = "black"))+ggtitle("Supposed Ecological Provenance")+ theme(plot.title = element_text(hjust = 0.5, size =30)) +theme(legend.key.height=unit(2,"line"))+theme(legend.title=element_text(size=20)) +scale_fill_uchicago()+coord_flip()
ggsave("Supposed_ecological_provenance.eps",device="eps", width = 15,height = 5,dpi = 600)
```
#Sourcetracker
```{r}
#Extract abundance matrix from the phyloseq object
filtered_asv_table <- as(otu_table(physeq_no_cont), "matrix")
#Coerce to data.frame
filtered_asv_table <- as.data.frame(filtered_asv_table)

#Extract only those samples in common between filtered asvs and slaughterhouse
common.sample.ids_slaughterhouse <- intersect(rownames(sample_data), rownames(filtered_asv_table))
asvs_slaughterhouse <- filtered_asv_table[common.sample.ids_slaughterhouse,]
metadata_slaughterhouse <- sample_data[common.sample.ids_slaughterhouse,]
# double-check that the mapping file and otu table
# had overlapping samples
if(length(common.sample.ids_slaughterhouse) <= 1) {
    message <- paste(sprintf('Error: there are %d sample ids in common '),
                    'between the metadata file and data table')
    stop(message)
}

#Extract the source environments and source/sink indices
train.ix_slaughterhouse <- which(metadata_slaughterhouse$SourceSink=='Source')
test.ix_slaughterhouse <- which(metadata_slaughterhouse$SourceSink=='Sink')

```
envs_slaughterhouse <- metadata_slaughterhouse$Env
if(is.element('Position',colnames(metadata_slaughterhouse))) desc <-
metadata_slaughterhouse$Position

#Load SourceTracker package
source('C:/Benni/src/SourceTracker.r')

#tune the alpha values using cross-validation (this is slow!)
#tune.results <- tune.st(otus[train.ix,], envs[train.ix])
#alpha1 <- tune.results$best.alpha1
#alpha2 <- tune.results$best.alpha2
#note: to skip tuning, run this instead:
alpha1 <- alpha2 <- 0.001

#Train SourceTracker object on training data
st_slaughterhouse <- sourcetracker(asvs_slaughterhouse[train.ix_slaughterhouse,],
envs_slaughterhouse[train.ix_slaughterhouse])

#Estimate source proportions in data
results_slaughterhouse <- predict(st_slaughterhouse,asvs_slaughterhouse[test.ix_slaughterhouse,],
alpha1=alpha1, alpha2=alpha2,burnin = 100, full.results = TRUE)

# ==================reading data out to plot more clearly=====================

PieNos_slaughterhouse = results_slaughterhouse$proportions
PieNos_slaughterhouse = as.data.frame(PieNos_slaughterhouse)

# in order to group and summarise samples (ie average) we will add metadata back
# and this will allow grouping of samples
  metadata_slaughterhouse$id = row.names(metadata_slaughterhouse)
  PieNos_slaughterhouse$id = row.names(PieNos_slaughterhouse)
  zz_slaughterhouse <- merge(PieNos_slaughterhouse, metadata_slaughterhouse, by = "id")

  require(dplyr)
  # grouping and summarising variables within a df called 'zz'
  # to get mean and sd of each

              PieAvs_slaughterhouse <- zz_slaughterhouse %>% # choose name of new df (here it's
PieAvs) to which new data will be put
                group_by(Position) %>% # group by sample

                # then use summarise to work out mean etc for each diff group, where each of these will
be a new column in new df

                summarise(`Anal Swab`= mean(`Anal Swab`),
                          `Classification (Gloves)` = mean(`Classification (Gloves)`),
                          `Classification (Railing)` = mean(`Classification (Railing)`),
                          `Cooling chamber (Wall)` = mean(`Cooling chamber (Wall)`),
                          `Evisceration (Aprons)` = mean(`Evisceration (Aprons)`),
                          `Evisceration (Gloves)` = mean(`Evisceration (Gloves)`),
                          `Evisceration (Knifes)` = mean(`Evisceration (Knifes)`),
                          `Lock (Hands)` = mean(`Lock (Hands)`),
```

```
                    `Lock (Shoes)` = mean(`Lock (Shoes)`),
                    `Lock brush (Clean area)` = mean(`Lock brush (Clean area)`),
                    `Lock liquid (Clean area)` = mean(`Lock liquid (Clean area)`),
                    `Polishing tunnel (Nozzles)` = mean(`Polishing tunnel (Nozzles)`),
                    `Polishing tunnel (Water)` = mean(`Polishing tunnel (Water)`),
                    `Polishing tunnel (Whips)` = mean(`Polishing tunnel (Whips)`),
                    `Splitting saw` = mean(`Splitting saw`),
                    Sticking = mean(Sticking),
                    `Truck (Gloves)` = mean(`Truck (Gloves)`),
                    `Truck (Wall)` = mean(`Truck (Wall)`),
                    Unknown = mean(Unknown))

        write.csv(PieAvs_slaughterhouse, "C://Benni/WP1 Pacbio reads
neu/Analysis/averagesOutPutPiesSourceTracker_slaughterhouse.csv")

#Sankey diagram
library(networkD3)

#melt sourcetracker output to make a connection dataframe and change column names
links <- melt(PieAvs_slaughterhouse)
links <- links[ , c("variable", "Position", "value")]
colnames(links) <- c("source", "target", "value")

#reorder sources to consecutive stations
links <-
links[c(1:5,76:80,56:60,61:65,66:70,21:25,26:30,31:35,71:75,6:10,11:15,16:20,36:40,41:45,46:50,5
1:55,81:85,86:90,91:95),]

#From these flows we need to create a node data frame: it lists every entities involved in the flow
nodes=data.frame(name=c(as.character(links$source), as.character(links$target)) %>% unique())
links$IDsource=match(links$source, nodes$name)-1
links$IDtarget=match(links$target, nodes$name)-1

#custom colors
# Add a 'group' column to each connection:
links$group=as.factor(c("a","a","a","a","a","b","b","b","b","b","c","c","c","c","c","d","d","d","d","d
","e","e","e","e","e","f","f","f","f","f","g","g","g","g","g","h","h","h","h","h","i","i","i","i","i","j","j"
,"j","j","j","k","k","k","k","k","l","l","l","l","l","m","m","m","m","m","n","n","n","n","n","o","o","o
","o","o","p","p","p","p","p","q","q","q","q","q","r","r","r","r","r","s","s","s","s","s"))

# Add a 'group' column to each node. Here I decide to put all of them in the same group to make
them grey
nodes$group=as.factor(c("my_unique_group"))

my_color <- 'd3.scaleOrdinal() .domain(["a", "b", "c", "d", "e", "f", "g", "h", "i", "j", "k", "l", "m",
"n", "o", "p", "q", "r",
"s","my_unique_group"]) .range(["#D3D3D3","#d44739","#77362c","#c88763","#d18d2e","#a1a3
4e","#48612f","#64b744","#5cb295","#5e90b8","#6477d3","#5938b4","#5b386e","#b14be1","#be7
fcc","#c842ae","#c887a0","#c64376","#D3D3D3"])'

d3test <- sankeyNetwork(Links = links, Nodes = nodes, Source = "IDsource", Target = "IDtarget",
Value = "value", NodeID = "name", colourScale=my_color, LinkGroup="group",
```

```
NodeGroup="group",iterations = 0,fontSize = 14, fontFamily = "Calibri")

saveNetwork(d3test, file =  "C://Benni/WP1 Pacbio reads
neu/Analysis/Sankey_diagram_slaughterhouse.html")

#Contribution of individual taxa

#The "results" object has a four-dimensional "full.results" array that is Num trials x Num envs x
Num OTUs x Num samples. So to see the number of observations of OTU 99 assigned to all
sources in sample 3 for all trials/restarts, you would enter:

results_slaughterhouse$full.results[,,99,3]

#To get a matrix of OTU x Probability(OTU came from source) across all samples
asv.env.probs_slaughterhouse <- t(apply(results_slaughterhouse$full.results,c(2,3),mean))

asv.env.probs_slaughterhouse <-
asv.env.probs_slaughterhouse[rowSums(asv.env.probs_slaughterhouse) > 0,]

asv.env.probs_slaughterhouse <-
sweep(asv.env.probs_slaughterhouse,1,rowSums(asv.env.probs_slaughterhouse),'/')

#Add correct rownames and columnnames and add taxaID
taxatable <- as(tax_table(physeq_no_cont),"matrix")
taxatable <- as.data.frame(taxatable)
row.names(asv.env.probs_slaughterhouse) <- row.names(taxatable)
colnames(asv.env.probs_slaughterhouse) <- c("Anal Swab","Classification
(Gloves)","Classification (Railing)","Cooling chamber (Wall)","Evisceration
(Aprons)","Evisceration (Gloves)","Evisceration (Knifes)","Lock (Hands)","Lock (Shoes)","Lock
brush (Clean area)","Lock liquid (Clean area)","Polishing tunnel (Nozzles)","Polishing tunnel
(Water)","Polishing tunnel (Whips)","Splitting saw","Sticking","Truck (Gloves)","Truck
(Wall)","Unknown")
asv.env.probs_slaughterhouse <- as.data.frame(asv.env.probs_slaughterhouse)

taxa_sourcetracker_slaughterhouse <- cbind(asv.env.probs_slaughterhouse,taxatable)
taxa_sourcetracker_slaughterhouse <- melt(taxa_sourcetracker_slaughterhouse)
taxa_sourcetracker_slaughterhouse <- taxa_sourcetracker_slaughterhouse[!
(is.na(taxa_sourcetracker_slaughterhouse$value) | taxa_sourcetracker_slaughterhouse$value==""), ]

#average of position and genus
Genus_sourcetracker_slaughterhouse <- taxa_sourcetracker_slaughterhouse %>%
  group_by(Genus,variable) %>%
  summarise(mean = mean(value))

Plot_taxa_sourcetracker_slaughterhouse <-
ggplot(Genus_sourcetracker_slaughterhouse, aes(x=Genus,y=mean, fill=variable)) +
geom_bar(stat='identity', position="fill")+ guides(fill = guide_legend(ncol = 1))
+labs(y="Probability", x="Genus") + theme(legend.text = element_text(size=15,face =
"italic"),axis.title=element_text(size=20),axis.text=element_text(size=10,angle = 90),strip.text.x =
element_text(size=20),strip.text.y = element_text(size=20))+scale_fill_manual(values =
colorRampPalette(brewer.pal(9,"Set1"))(20))
ggsave("C://Benni/WP1 Pacbio reads
```

```
neu/Analysis/Genus_sourcetracker_slaughterhouse.pdf",device="pdf", width = 15,height = 10,dpi = 600)
dev.off()

#Genera of interest (Genera that include species associated with meat spoilage)
genus_list <-
c("Acinetobacter","Aeromonas","Antricoccus","Arthrobacter","Bacillus_S","Bacillus_L","Brochothrix","Campylobacter_C","Carnobacterium_A","Chryseobacterium","Chryseobacterium_A","Chryseobacterium_B","Chryseobacterium_D","Citrobacter","Clostridium","Corynebacterium","Escherichia","Flavobacterium","Flavobacterium_A","Fusobacterium","Fusobacterium_C", "Lactobacillus","Lactobacillus_D","Lactobacillus_H","Lactococcus","Leuconostoc","Listeria","Micrococcus","Moraxella","Pediococcus","Porphyromonas","Proteus","Propionibacterium","Pseudomonas_E","Psychrobacter","Salmonella","Serratia","Streptococcus","Staphylococcus","Staphylococcus_A", "Weissella")

Genus_sourcetracker_slaughterhouse$Genus <-
as.character(Genus_sourcetracker_slaughterhouse$Genus)

#Make new column with all genera not on list renamed as "Other"
Genus_sourcetracker_slaughterhouse_2 <- Genus_sourcetracker_slaughterhouse %>%
  rowwise() %>%
  mutate(Genus_n = if_else(Genus %in% genus_list, Genus, "Other")) %>%
  ungroup()

Genus_sourcetracker_slaughterhouse_2$variable <-
factor(Genus_sourcetracker_slaughterhouse_2$variable, levels= c("Anal Swab", "Sticking","Lock brush (Clean area)", "Lock liquid (Clean area)","Lock (Hands)", "Lock (Shoes)","Polishing tunnel (Whips)", "Polishing tunnel (Nozzles)", "Polishing tunnel (Water)",  "Evisceration (Gloves)", "Evisceration (Knifes)", "Evisceration (Aprons)", "Splitting saw", "Classification (Railing)", "Classification (Gloves)", "Cooling chamber (Wall)", "Truck (Gloves)", "Truck (Wall)", "Unknown"))

Relevant_taxa_sourcetracker_plot <- ggplot(Genus_sourcetracker_slaughterhouse_2,
aes(x=variable,y=mean, fill=Genus_n)) + geom_bar(stat='identity', position="fill")+ guides(fill = guide_legend(ncol = 1))+labs(y="Probability of Origin", x="Position") + theme(legend.text = element_text(size=15,face = "italic"),axis.title=element_text(size=20),axis.text=element_text(size=10),strip.text.x = element_text(size=20),strip.text.y = element_text(size=20,angle = 90))+scale_fill_manual(values = relevant_taxa_palette)+scale_x_discrete(limits = rev(levels(Genus_sourcetracker_slaughterhouse_2$variable)))+coord_flip()+ggtitle("Origin of relevant taxa on Truck samples")
ggsave("C://Benni/WP1 Pacbio reads
neu/Analysis/Relevant_taxa_origin_slaughterhouse.jpg",device="jpg", width = 15,height = 10,dpi = 600)
dev.off()

Relevant_taxa_sourcetracker_plot_data <- Relevant_taxa_sourcetracker_plot$data
Relevant_taxa_sourcetracker_plot_data_heatmap <-
dcast(Relevant_taxa_sourcetracker_plot_data,Genus_n~variable, value.var="mean",fun.aggregate = mean)
rownames(Relevant_taxa_sourcetracker_plot_data_heatmap) <-
Relevant_taxa_sourcetracker_plot_data_heatmap[,1]
```

````
Relevant_taxa_sourcetracker_plot_data_heatmap[,1] <- NULL
Relevant_taxa_sourcetracker_plot_data_heatmap <-
as.matrix(Relevant_taxa_sourcetracker_plot_data_heatmap)

#Export heatmap data, add 16S qPCR data and import again
write.table(Relevant_taxa_sourcetracker_plot_data_heatmap, file = "C://Benni/WP1 Pacbio reads
neu/Analysis/heatmapdata.csv", sep = ";",dec = ",")

heatmapdata <- read.table(file = "C://Benni/WP1 Pacbio reads neu/Analysis/heatmapdata.csv", sep
= ";", dec = ",",header = TRUE,row.names = 1)

heatmapdata <- t(heatmapdata)
heatmapdata <- as.data.frame(heatmapdata)

#invert row order
heatmapdata <- heatmapdata[order(nrow(heatmapdata):1),]

#heatmap
setEPS()
postscript("C://Benni/WP1 Pacbio reads neu/Analysis/Relevant_taxa_sourcetracker_heatmap.eps")
superheat(dplyr::select(heatmapdata, -c(qpcr,logqpcr)),bottom.label.text.angle = 90, col.dendrogram
= TRUE,yr=heatmapdata$logqpcr, yr.axis.name = "LOG BCE/cm2",yr.plot.type = "bar")
```
#Statistics for sourcetracker performance at different sequencing depths
```{r}
library(car)
library(dunn.test)
library(pracma)

#-------------"Goodness of fit" analysis--------------
#Import data
Data=read.csv(file.path("F:","1909Zwirzitz","gof.CSV"),sep=";",header=TRUE)

#Exclude "total" data
Data = subset(Data,Data$solution!="total")
Data$solution <- factor(Data$solution)
#Distribution check of the squared differences
res.aov <- aov(diffsq ~ solution, data = Data)
plot(res.aov,2)
aov_residuals <- residuals(object = res.aov )
shapiro.test(x = aov_residuals)
#Check for homoscedasticity
#leveneTest(diffsq ~ solution,data=Data)
#Kruskal Wallis test
kruskal.test(diffsq~solution,data=Data)
#post hoc Dunn Test
dunn.test(Data$diffsq, g=Data$solution,method="BH")

#Mean squared differences "Goodness of fit"
library (rcompanion)
groupwiseMean(diffsq~solution, data=Data,conf=0.95, R=5000, percentile = T, bca=F, digits=3)
````

```
#-------------"Hit ratio" analysis-------------
#Import Data
Data=read.csv(file.path("F:","1909Zwirzitz","hitratio.CSV"),sep=";",header=TRUE)

#Distribution check of the squared differences
res.aov <- aov(trefferquote ~ solution*location, data = Data)
plot(res.aov,2)
aov_residuals <- residuals(object = res.aov )
shapiro.test(x = aov_residuals)
#Check for homoscedasticity
#leveneTest(trefferquote ~ solution,data=Data)
#Kruskal Wallis test
kruskal.test(trefferquote~solution,data=Data)
#post hoc Dunn Test
dunn.test(Data$trefferquote, g=Data$solution,method="BH")

#Mean hit ratio%
groupwiseMean(trefferquote~solution, data=Data,conf=0.95, R=5000, percentile = T, bca=F,
digits=3)


#--------------"classification rate" analysis------------
#Import for unknown classification
Data=read.csv(file.path("F:","1909Zwirzitz","unknownclass.CSV"),sep=";",header=TRUE)

#groupwise Means "Mean unknwon classification rate %"
groupwiseMean(rate~solution, data=Data,conf=0.95, R=5000, percentile = T, bca=F, digits=3)

#Exclude "total" data
Data<-subset(Data, Data$solution!="all")
#Distribution check
res.aov <- aov(diffunknown ~ solution, data = Data)
plot(res.aov,2)
aov_residuals <- residuals(object = res.aov )
shapiro.test(x = aov_residuals)
#Check for homoscedasticity
leveneTest(diffunknown ~ solution,data=Data)
#Kruskal Wallis test
kruskal.test(diffunknown~solution,data=Data)
#post hoc Dunn Test
dunn.test(Data$diffunknown, g=Data$solution,method="BH")

#"Mean difference fo unknown classification rate%"
groupwiseMean(diffunknown~solution, data=Data,conf=0.95, R=5000, percentile = T, bca=F,
digits=3)


#--------------Figure 7A-------------
#Import data Figure 7A
Data=read.csv(file.path("F:","1909Zwirzitz","datafig7a.CSV"),sep=";",header=TRUE)
summary(Data)
```

```
#Figure 7A
plot(Data$all, Data$mean200, col="red", xlab="classification rate of the orignal dataset",
ylab="classification rate of different dataset sizes",pch=1)
points(Data$all, Data$mean500, col = "orange",pch=2)
points(Data$all, Data$mean1000, col = "yellow",pch=3)
points(Data$all, Data$mean5000, col = "lightgreen",pch=4)
points(Data$all, Data$X7712_01, col ="lightblue",pch=5)
lines(c(0,0.4),c(0,0.4),col="black")
legend(0, 0.5, legend=c("200 seq","500 seq","1000 seq","5000 seq","7712 seq","all seq"),
    col=c("red","orange","yellow","lightgreen","lightblue","black"),pch=c(1,2,3,4,5,NA),
lty=c(0,0,0,0,0,1), cex=0.8)



#-------------Figure 7B----------------
#Import Date Figure 7B
Data=read.csv(file.path("F:","1909Zwirzitz","datafig7b.CSV"),sep=";",header=TRUE)
summary(Data)


#Preparations Figure 7B
xq1 = 0:8000
p = pchip(Data$solution,Data$meantq,xq1)
p1 = pchip(Data$solution,Data$X25tq,xq1)
p2 = pchip(Data$solution,Data$mintq,xq1)
p3 = pchip(Data$solution,Data$X75tq,xq1)
p4 = pchip(Data$solution,Data$maxtq,xq1)
p5 = pchip(Data$solution,Data$meanuk,xq1)
p6 = pchip(Data$solution,Data$X25uk,xq1)
p7 = pchip(Data$solution,Data$minuk,xq1)
p8 = pchip(Data$solution,Data$X75uk,xq1)
p9= pchip(Data$solution,Data$maxuk,xq1)

#Figure 7B
par(family="sans")
plot(Data$solution,100*Data$meantq,ylim=c(0,100),lty=1, xlab= "dataset size", ylab="hit ratio vs.
unknown classification rate [in %]", col="white")
polygon(c(xq1,rev(xq1)),c(100*p7,rev(100*p9)),col="#f0f0f0",lty=0)
polygon(c(xq1,rev(xq1)),c(100*p6,rev(100*p8)),col="#bdbdbd",lty=0)
lines(xq1,100*p7,lty=3,lwd=0.5,col="#636363")
lines(xq1,100*p9,lty=3,lwd=0.5,col="#636363")
lines(xq1,100*p6,lty=2,lwd=0.5,col="#636363")
lines(xq1,100*p8,lty=2,lwd=0.5,col="#636363")
library(ggplot2)
polygon(c(xq1,rev(xq1)),c(100*p2,rev(100*p4)),col=alpha("#f0f0f0",0.5),lty=0)
polygon(c(xq1,rev(xq1)),c(100*p1,rev(100*p3)),col=alpha("#bdbdbd",0.5),lty=0)
lines(xq1,100*p5,lty=1,lwd=2, col = "#31a354")
points(Data$solution,100*Data$meanuk,pch=20, col="#31a354")
lines(xq1,100*p, lty=1,lwd=2, col ="#e34a33" )
lines(xq1,100*p2,lty=3,lwd=0.5,col="#636363")
lines(xq1,100*p4,lty=3,lwd=0.5,col="#636363")
```

```
lines(xq1,100*p1,lty=2,lwd=0.5,col="#636363")
lines(xq1,100*p3,lty=2,lwd=0.5,col="#636363")
points(Data$solution,100*Data$meantq,pch=20, col="#e34a33")
```
